# Exploratory analysis of the proteomic profile in plasma in adults with Down syndrome in the context of Alzheimer's disease

**DOI:** 10.1002/alz.70040

**Published:** 2025-03-20

**Authors:** Olivia Wagemann, Georg Nübling, Francisco Jesús Martínez‐Murcia, Elisabeth Wlasich, Sandra V. Loosli, Katja Sandkühler, Anna Stockbauer, Catharina Prix, Sabrina Katzdobler, Agnese Petrera, Stefanie M. Hauck, Juan Fortea, Rocío Romero‐Zaliz, Carmen Jiménez‐Mesa, Juan M. Górriz Sáez, Günter Höglinger, Johannes Levin

**Affiliations:** ^1^ Department of Neurology University Hospital, Ludwig‐Maximilians‐University (LMU) Munich Munich Germany; ^2^ German Center for Neurodegenerative Disease (DZNE) Munich Germany; ^3^ Department of Signal Theory Telematics and Communications Andalusian Institute in Data Science and Computational Intelligence (DaSCI) at University of Granada Granada Spain; ^4^ Department of Neurology University Hospital Zurich Zurich Switzerland; ^5^ Metabolomics and Proteomics Core Helmholtz Zentrum München, German Research Center for Environmental Health (GmbH) Neuherberg Germany; ^6^ Sant Pau Memory Unit Hospital de la Santa Creu i Sant Pau ‐ Biomedical Research Institute Sant Pau Barcelona Spain; ^7^ Centro de Investigación Biomédica en Red de Enfermedades Neurodegenerativas, CIBERNED Madrid Spain; ^8^ Barcelona Down Medical Center, Fundació Catalana Síndrome de Down Barcelona Spain; ^9^ Information and Communication Technologies Research Centre (CITIC‐UGR) University of Calle Periodista Rafael Gómez Montero Granada Spain; ^10^ Munich Cluster for Systems Neurology (SyNergy) Munich Germany

**Keywords:** Alzheimer's disease, biomarker, Down syndrome, neuroinflammation, plasma

## Abstract

**INTRODUCTION:**

Adults with Down syndrome (DS) show increased risk for Alzheimer's disease (AD) due to the triplication of chromosome 21 encoding the *amyloid precursor protein* gene. Further, this triplication possibly contributes to dysregulation of the immune system, furthering AD pathophysiology.

**METHODS:**

Using Olink Explore 3072, we measured ∼3000 proteins in plasma from 73 adults with DS and 15 euploid, healthy controls (HC). Analyses for differentially expressed proteins (DEP) were carried out, and pathway and protein network enrichment using Gene Ontology, Kyoto Encyclopedia of Genes and Genomes (KEGG), and STRING database was investigated. Within DS, the LASSO (least absolute shrinkage and selection operator) feature selection was applied.

**RESULTS:**

We identified 253 DEP between DS and HC and 142 DEP between symptomatic and asymptomatic DS. Several pathways regarding inflammatory and neurodevelopmental processes were dysregulated in both analyses. LASSO feature selection within DS returned 15 proteins as potential blood markers.

**DISCUSSION:**

This exploratory proteomic analysis found potential new blood biomarkers for diagnosing DS‐AD in need of further investigation.

**Highlights:**

- Inflammatory pathways are dysregulated in symptomatic versus asymptomatic DS.
- NFL and GFAP are confirmed as powerful biomarkers in DS with clinical and/or cognitive decline.
- Further circulating proteins were identified as potential blood biomarkers for symptomatic DS.

## 1 BACKGROUND

In Alzheimer's disease (AD), amyloid‐plaques and neurofibrillary tau tangles (NFT) make up the pathophysiological hallmarks causing neurodegeneration,^1^, ^2^ known to cause decline in cognitive capacity and everyday functioning. However, other processes like vascular dysfunction, oxidative stress, endosomal and lysosomal dysfunction, and neuroinflammation are likely to play a pivotal role as well.^3^, ^4^

Adults with Down syndrome (DS) present with a triplication of the *amyloid precursor protein* (*APP*) *gene*, due to the presence of three copies of chromosome 21 (chr21),^5^ which results in the early accumulation of intracellular amyloid already in childhood,^6^ eventually leading to extracellular accumulation of amyloid, the formation of solid amyloid‐plaques, and subsequent intracellular tau phosphorylation, ultimately resulting in progressive cognitive impairment.^7^

Even though DS‐AD largely parallels the pathophysiological and clinical presentation of AD in the carriers of the autosomal dominant gene mutations in *presenilin 1* (*PSEN1)*, *presenilin 2* (*PSEN2)*, or *APP*, the diagnosis of dementia due to DS‐AD remains challenging, predominantly due to the heterogeneous presentation of clinical symptoms, widely varying level of baseline cognitive capacity and the lack of standardized tests to assess and evaluate these.^8^, ^9^

Therefore, diagnosis often relies on external anamnesis since those affected may not recognize or prioritize memory decline as a significant issue. Behavioral changes or loss of daily living skills are more frequently reported, but these symptoms can be highly variable.^10^, ^11^ The lack of awareness among families, caregivers, and clinicians exacerbates these diagnostic challenges,^12^ which can lead to a significant delay of the diagnosis.

A lot of effort has been put into researching biomarkers in DS‐AD by assessing AD pathology in brain imaging, cerebral spinal fluid (CSF), and blood. Considering cost‐effectiveness, accessibility in a clinical setting, and the minimally invasive nature of sample obtainment, blood‐based biomarkers are of high interest, potentially mirroring AD‐related changes of the brain in DS within in the peripheral bloodstream.^13^

Levels of Aβ42 in plasma have been reported to be elevated in DS, yet reports on the diagnostic and prognostic performance of both Aβ42 and Aβ42/40 ratio remain inconsistent.^14^, ^15^, ^16^, ^17^, ^18^ Markers of tau pathology, however, namely pTau‐181 and pTau‐217, reliably differentiate between symptomatic and asymptomatic DS and have been reported to correlate well with tau and amyloid pathology as assessed by positron emission tomography (PET).^14^, ^19^, ^20^, ^21^ Further, plasma levels of neurofilament light chain (NFL), a marker of axonal degeneration, rise early and correlate well with cognitive decline^22^ as well as amyloid load and neurodegeneration in brain imaging, offering strong diagnostic utility.^15^, ^23^ Finally, glial fibrillary acidic protein (GFAP), relating to astrocytic activity, rises as early as in the third decade, correlates well with cognitive status as well as tau and amyloid burden in PET, and shows good performance for predicting disease progression, which interestingly could not be replicated in CSF.^21^, ^24^ Recently, we have also shown that the presynaptic marker beta‐synuclein provides good discriminatory power in DS‐AD, rising even before pTau‐181.^25^

Despite the genetic basis of DS‐AD, resulting in a predictable sequence of pathophysiological events,^5^ significant variability in symptom onset and presentation remains, warranting the investigation of additional pathophysiological processes that might be directly or indirectly caused by increased levels of proteins originating from chr21.^26^, ^27^, ^28^, ^29^ A neuroinflammatory phenotype in DS‐AD, that is, distinct from sporadic AD (sAD) has been suggested^27^ and neuropathological studies reported a neuroinflammatory protein profile evolving across the DS‐AD continuum.^26^ In blood, differences in circulating inflammatory proteins compared to euploids have been identified, in line with chronic autoinflammation possibly contributing to DS‐AD^29^ while another study, targeting 20 inflammation‐related proteins, further found differences in DS‐AD and mildly impaired DS compared to asymptomatic DS with excellent accuracy,^30^ underscoring the need to further investigate interconnected processes through exploratory proteomic approaches.

We aimed to explore the proteomic profile of individuals with DS using OLINK technology to identify proteins and pathways influencing AD progression thereby leveraging a broader, exploratory approach. The findings may uncover biomarkers and therapeutic targets, providing a foundation for future hypothesis‐driven research into DS‐AD pathophysiology.

## 2 METHODS

### 2.1 Participants and clinical workup

All participants included in this investigation are either part of our AD21 cohort study or our study for biological samples in neurodegeneration (Biobank für translationale Neurodegeneration), and were recruited from the outpatient clinic at LMU University Hospital Munich in Germany. The former, AD21, investigates AD in adults with DS, using clinical characterization, neuropsychology, neuroimaging, and biofluid analysis with annual follow‐up visits. Further information on the study design as well as inclusion and exclusion criteria can be found in the supplements (Table S1). The latter study provided the euploid healthy controls (HC) included in this analysis and aims at collecting biofluids and basic demographic information within the framework of a biobank. Included in this analysis were HC from this study where clinically as well as by patient account there was no sign of cognitive decline and no hint at cognitive symptoms impacting daily living activity and further no diagnosis of cognitive impairment of any kind, known structural brain lesions, or the diagnosis of a neurodegenerative disease.

Each individual or their respective legal proxy provided informed written consent prior to inclusion. Both studies are approved by the LMU ethics committee (DS: #17‐126, HC: #20‐0997) and conducted in accordance with the Declaration of Helsinki.

Our study sample reflects the demographics of adults with DS in Germany, where participants were predominantly of European descent, specifically White/Caucasian. While ethnic diversity within this cohort is limited, we sought to ensure inclusivity by recruiting participants from various age groups as well as intellectual disability.

**RESEARCH IN CONTEXT**

1. **Systematic review**: The authors conducted a comprehensive review of the existing literature leveraging established databases (e.g., PubMed, Google Scholar). There were very few publications using proteomics to explore blood samples in Down syndrome (DS), especially in the context of Alzheimer's disease (AD) or neuroinflammation, those uncovered are cited accordingly in the manuscript. We identified what we consider critical gaps in proteomic analyses in Down syndrome, setting the stage for this study's proteomic approach using the Olink Explore 3072 platform.
2. **Interpretation**: Our findings contribute new insights into DS‐AD by identifying key pathways associated with immune and neurodegenerative processes as well as providing potential blood biomarkers for AD in the DS population which may serve as early indicators or therapeutic targets.
3. **Future directions**: Longitudinal analyses are needed to verify the current results and to further investigate the mechanistic pathways linking immune dysregulation with neurodegeneration in Down syndrome.

Chromosome analysis in DS was conducted to assess the accurate type of trisomy 21 (full, translocation, mosaicism) where possible. Intellectual disability (ID) was stratified according to Diagnostic and Statistical Manual of Mental Disorders, 5th edition (DSM‐V) criteria^31^ into mild, moderate, severe, or profound, based on the individuals’ best‐ever level of functioning, obtained from interviews with caregivers, neuropsychological assessment, behavioral observations, and review of medical records.

Symptomatic diagnosis was reached independently by two neurologists referencing a predefined diagnostic algorithm^9^: changes in cognition, behavior, and activities of daily living were assessed based on patient and caregiver information. Subsequently, differential diagnoses were excluded via neurological, laboratory blood test, and individual cognitive performance assessments performed by trained neuropsychologists using the validated German version of the Cambridge Cognitive Examination for Older Adults with Down Syndrome (CAMCOG‐DS).^8^, ^32^ The CAMCOG‐DS consists of 45 items and 7 subscales (orientation, language, memory, attention, praxis, abstract thinking, and visual perception). A more detailed description of the specific tasks can be found elsewhere.^8^ This eventually led to the diagnosis of either asymptomatic DS (aDS) or symptomatic DS (sDS), the latter including dementia as well as mild cognitive impairment in the context of DS‐AD.

Alzheimer's pathology was further verified referencing the A/T/N criteria,^1^ where possible, using validated AD biomarkers such as cerebral spinal fluid (CSF) for assessment of phospho‐tau‐181 (pTau181, cutoff 61 pg/mL) and Aβ1‐42/1‐40 ratio (Aβ‐ratio, cutoff 5.5%) with the respective Innotest assays. Further, if possible, cerebral imaging was acquired as part of the clinical workup. For this, magnetic resonance imaging (MRI) including T1‐ (repetition time [TR] 2560 ms, TI: 1100 ms, flip angle: 7°, CAIPIRINHA = 2, field of view [FOV] = 256 mm × 256 mm, slice thickness 0.8 mm, 224 slices per slab) and T2‐weighted sequences (TR 2800 ms, echo time [TE] 405 ms, GRAPPA = 2, FOV = 256 mm × 256 mm, slice thickness 0.8 mm, 224 slices per slab) as well as a fluid‐attenuated inversion recovery sequence (TR 5000 ms, TE 393 ms, GRAPPA = 2, FOV = 256 mm × 256 mm, slice thickness 1 mm, 176 slices per slab) were obtained using a 3 Tesla Siemens Scanner (Siemens, Erlangen, Germany). Positron emission tomography (PET) imaging was performed with a Siemens Biograph‐64 system. 18F‐florbetaben PET (295 MBq) images were acquired 90–110 min after injection. 18F‐PI‐2620‐PET (185 MBq) was performed using dynamic emission recording (0–60 min after injection, 30–60 min window for visual interpretation). For intensity scaling, a cerebellar grey matter reference was used for all tracers.

### 2.2 Collection and measurement of plasma samples

Blood samples for both study cohorts were collected into ethylenediaminetetraacetic acid (EDTA) tubes from non‐fasting participants, centrifuged at 2000 × *g* for 10 min to obtain plasma, aliquoted into polypropylene tubes, and subsequently stored at −80°C within 30–45 min after initial blood drawl.

For protein analysis, we leveraged the OLINK Explore 3072 Panel (Uppsala, Sweden; Lot #B31341; consisting of the following individual Explore 384 kits: Inflammation Lot #B24609, Inflammation II Lot #B23708, Oncology Lot #B23705, Oncology II Lot #B24546, Cardiometabolic Lot #B23706, Cardiometabolic II Lot #B23710, Neurology Lot #B23707, Neurology II Lot #B23711), a multiplex immunoassay platform designed for human protein biomarker discovery, measuring the concentration of approximately 3000 proteins in one bio sample.^33^ For this, blood samples had to be thawed, pipetted on the assay plate, and refrozen before being sent to OLINK for measurement. This proximity extension assay uses highly specific antibodies that bind to their respective protein and hybridize their attached nucleotide single strands with neighboring antibodies on the protein's surface. The resulting double strand is then amplified and quantified via next‐generation‐sequencing read‐out. After quality control, the final data set is obtained as an arbitrary unit of normalized protein expression values (NPX, log2‐transformed). For this run, the intra‐assay coefficient of variation (%CV) for the proteins measured in each kit ranged from 6%CV to 13%CV, with a median of 9%CV. The limit of detection (LOD), calculated for each kit individually, ranged from −11.21 to 2.11.

### 2.3 Statistical analysis

All statistical analyses were run on R (version 2023.06.1+524). Exploratory proteomics analysis was conducted using the R packages Olink Analyze, clusterProfiler, glmnet as well as the STRING database website.^34^

Baseline demographics were summarized as mean ± standard deviation for continuous variables and count percentage for categorical variables. Group comparisons were conducted via Mann–Whitney *U*‐tests for continuous and Fisher's exact tests for categorical variables.

*t*‐Tests investigating differentially expressed proteins (DEP) between DS and HC as well as symptomatic and asymptomatic DS were carried out two‐sided. An uncorrected *p*‐value of less than 0.05 was considered statistically significant; in the case of multiple comparisons, the Benjamin–Hochberg method for false discovery rate was applied, with a *p*‐value below 0.05 set as a significance threshold.

All obtained DEP were analyzed for enrichment of biological processes, molecular functions, and specific cellular component using the Gene Ontology (GO, accessed 03/24024) database^35^ and investigated for enrichment of biological signaling pathways using the Kyoto Encyclopedia of Genes and Genomes (KEGG, version 109).^36^ Protein–protein interactions (PPI) between DEP were assessed with the STRING database (version 12.0).^34^ All proteins measured on the panel were set as background reference.

Feature selection was performed using absolute shrinkage and selection operator (LASSO) regression with an l1 penalty and a regulation parameter lambda set to 0.107 as chosen by cross‐validation. For further analysis, a train and test subset were generated using 80% and 20% of the whole data set, respectively. Weights, accounting for the imbalance in diagnosis outcome, were applied. The resulting selection of proteins relevant to predicting the outcome variable was then further used to assess their diagnostic performance via receiver operating characteristics (ROC) analysis.

## 3 RESULTS

### 3.1 Demographics

In this study, we included 73 adults with DS and 15 euploid HC. There were no significant differences in age or sex distribution (Table 1, both *p* > 0.05). As expected, there was a significant difference between the age of sDS and aDS, with the latter being around 20 years younger (*p* < 0.001). Further, we found a significantly worse performance on the CAMCOG‐DS in the symptomatic DS group (*p* < 0.001).

**TABLE 1:** Demographics of all participants included in this study.

Parameter	DS, *N* = 73	aDS, *N* = 49	sDS, *N* = 24	HC, *N* = 15
Age at visit (years)	38 ± 12	32 ± 8	51 ± 8	44 ± 17
Sex				
Female	34 (47%)	24 (49%)	10 (42%)	9 (60%)
Male	39 (53%)	25 (51%)	14 (58%)	6 (40%)
Leucocyte blood level (µL^−1^)		5.7 ± 1.4	5.8 ± 1.4	
*NA*		*6*	*3*	
Diagnosis of AID	49 (67%)	36 (74%)	13 (60%)	
CAMCOG‐DS (%)		63 ± 17	46 ± 18	
*NA*		*2*	*1*	
Amyloid (A) status				
Positive		0	12	
Negative		3	0	
*NA*		*46*	*12*	
Tau (T) status				
Positive		0	5	
Negative		1	3	
*NA*		*48*	*16*	
Neurodegeneration (N) status				
Positive		*0*	*10*	
Negative		*9*	*1*	
*NA*		*40*	*13*	

Abbreviations: aDS, asymptomatic Down syndrome; AID, autoimmune disease; CAMCOG‐DS, Cambridge Cognitive Examination for Older Adults with Down Syndrome; DS, Down syndrome; HC, healthy control; NA, not available; sDS, symptomatic Down syndrome.

### 3.2 DS versus HC

**FIGURE 1 alz70040-fig-0001:**
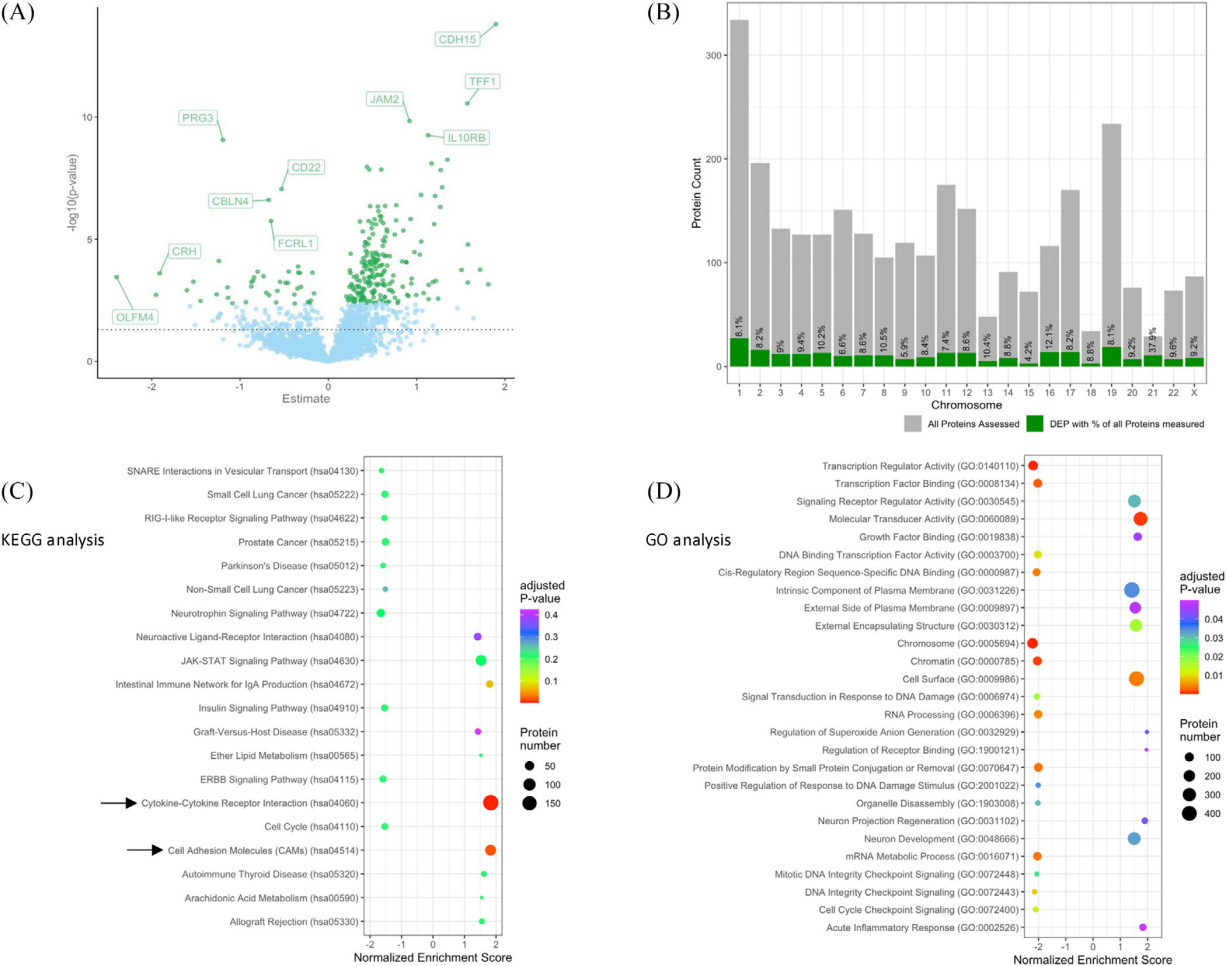
(A) Volcano plot displaying the differences of DEP in adults with DS compared to HC. Accordingly, 211 DEP were found to be increased (e.g., CDH15, TFF1, and IL10RB) and 42 DEP showed reduced circulating protein levels in DS versus HC (e.g., OLFM4, PRG3, and CBLN4). (B) Distribution of all DEP (green) in relation to all proteins assessed (grey) and their respective chromosome revealed 11 DEP (37.9%) originating from chr21. (C) Top 20 enriched pathways from KEGG analysis in DS versus HC with the 10 most positive and negative normalized enrichment score (NES), respectively, and where the size of the dot refers to the number of proteins that have been put in for analysis and are annotated in the respective term. Here, cytokine–cytokine receptor interaction and CAMs turned up significantly enriched (arrows). (D) Significantly enriched terms from GO analysis for increased and suppressed pathways in DS compared to HC. DEP, differentially expressed proteins; DS, Down syndrome; GO, Gene Ontology; HC, healthy control; KEGG, Kyoto Encyclopedia of Genes and Genomes.

Between DS and HC, we found 253 proteins exhibiting different plasma levels after false discovery rate (FDR) correction. Out of these, 211 were increased (Figure 1A) in DS, while the remaining 42 showed decreased levels in DS compared to HC. Mapping each of these DEP to their originating chromosome, we found 11 proteins to be encoded on chr21, all of them with higher levels in DS (Figure 1B).

To further gain understanding of the relevance of the proteomic differences between both groups for biological and molecular function as well as known signaling pathways in humans, we conducted protein enrichment analysis. Utilizing the KEGG database, we found two significantly enriched processes, namely cytokine–cytokine receptor interaction (hsa04060) and cell adhesion molecules (hsa04514) connected with increased protein levels in DS. When assessing the proteomes with the GO database, we found enriched processes of acute inflammatory response, cell binding and regulatory signaling, and neuronal growth connected to significantly increased protein levels, while significantly reduced protein levels in DS were matched to several pathways relating to the cell cycle, RNA and DNA processing as well as chromatin and DNA integrity, cellular stress, and macromolecular biosynthesis (Figure 1C,D). A comprehensive list of all significant pathway findings between the groups can be found in the supplements (Table S2).

For a better understanding of the functional protein networks affected by the discovered DEP, we assessed PPI with the STRING database^34^ to visualize and further categorize them into functional clusters. Enrichment within each cluster was further analyzed leveraging the GO, KEGG, and Reactome^37^ database (Table S3, Figure S1).

DEP between DS and HC were clustered into nine subnetworks with the largest one consisting of 162 DEP showing functional enrichment, among others, for cytokine–cytokine receptor interaction (hsa04060), immune system processes (GO:0002376), transmembrane signaling receptor activity (GO:0004888), and cytokine signaling in the immune system (HSA‐1280215). The second largest cluster, made up of 16 DEP, was associated with synaptic function such as PPI at synapses (HSA‐6794362) as well as voltage‐gated potassium channel complexes (GO:0008076) and gama‐aminobutyric acid (GABA)‐ergic synapses (GO:0098982). For another cluster with eight corresponding DEP, we found protein interactions relating to the lipid metabolic process (GO:0006629) and phospholipid metabolism (HSA‐1483257). Some for the remaining clusters reported associations of protein interactions included azurophile granules (GO:0042582) and lysosomes (GO:0043202, 4 DEP) as well as carbonate dehydratase activity (GO:0004089) and nitrogen metabolism (hsa00910, 2 DEP).[Fig alz70040-fig-0001]

### 3.3 sDS versus aDS

**FIGURE 2:**
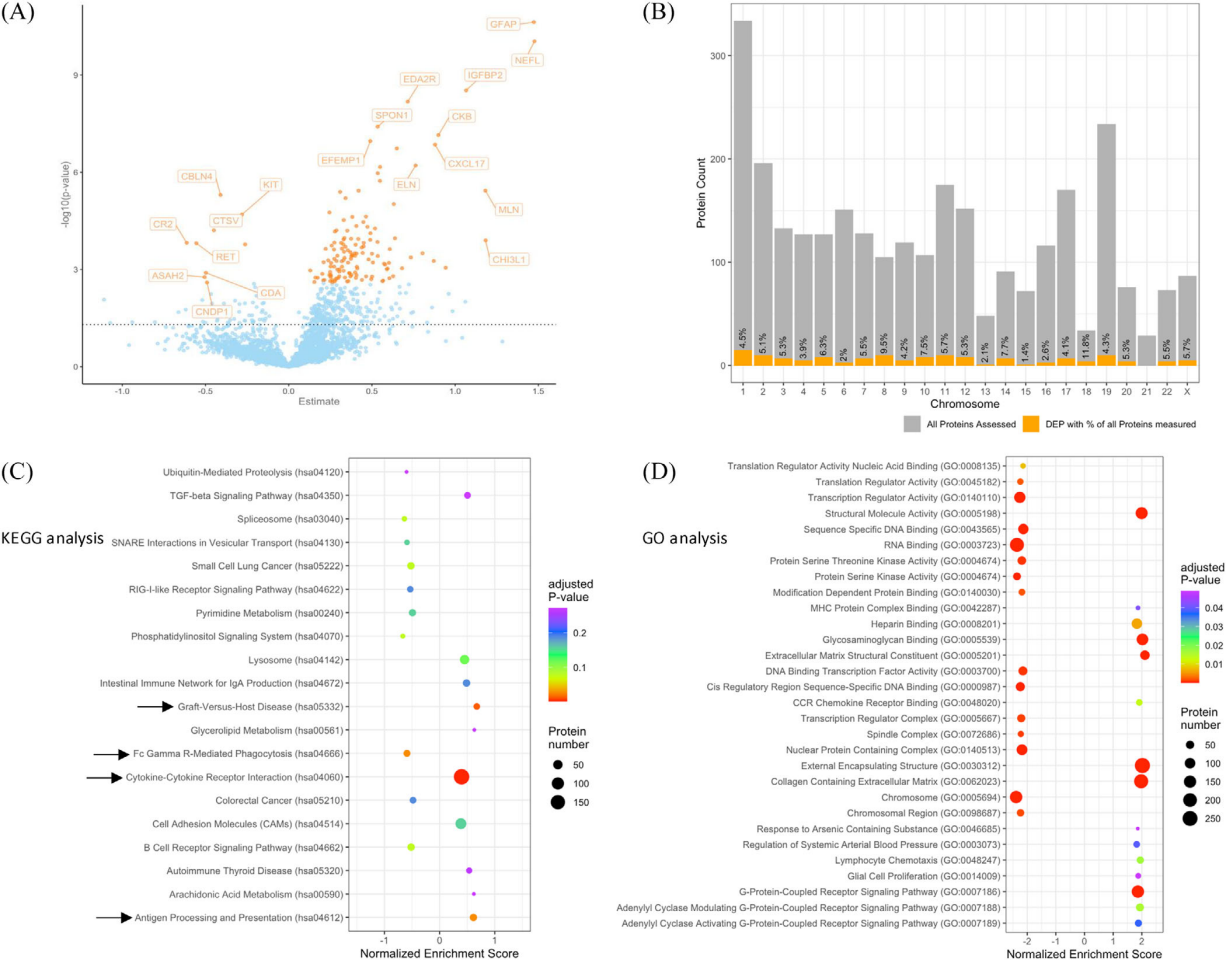
(A) Volcano plot displaying the differences of DEP in symptomatic versus asymptomatic adults with DS. Here, 133 DEP were found to with increased levels (e.g., GFAP, NEF, and IGFBP2) and 9 DEP showed decreased protein levels (e.g., CBLN4, CR2, and RET). (B) Distribution of all DEP (orange) in respect to all proteins assessed (grey) between symptomatic and asymptomatic DS regarding their respective chromosome with none of them originating from chr21. (C) Top 20 terms from KEGG Analysis, with 3 of them showing significant positive enrichment for symptomatic DS, and 1 negatively enriched pathway (arrows). (D) Significant terms from GO analysis for top enhanced or diminished pathways, respectively, in symptomatic versus asymptomatic DS. DEF, differentially expressed proteins; DS, Down syndrome; GFAP, glial fibrillary acidic protein; IGFBP2, insulin‐like growth factor binding protein‐2; KEGG, Kyoto Encyclopedia of Genes and Genomes; NEF, negative regulatory factor.

In the interest of discovering potential plasma biomarkers for the diagnosis and/or progression of DS‐AD, we repeated this analysis in the subgroup of adults with DS, dichotomizing them by their clinical diagnosis of being either symptomatic or asymptomatic. Here, we found 142 DEP after FDR correction. Specifically, 133 of DEP were increased in symptomatic DS, while the remaining nine DEP exhibited decreased levels in this group. Interestingly, none of the DEP originated from chr21 (Figure 2A,B).

When assessing significantly enriched signaling pathways with the KEGG database, we found cytokine–cytokine receptor interaction as significantly enriched in symptomatic DS, in addition to antigen processing and presentation and graft‐versus‐host‐disease. One pathway seemed to be suppressed in the symptomatic phenotype which was the Fc‐γ‐R‐mediated phagocytosis. Within the GO database, symptomatic adults with DS showed significant enrichment in 139 GO terms. Of them, 33 were associated with pathway activation, including immune‐related processes such as lymphocyte chemotaxis, major histocompatibility complex (MHC)‐protein complex binding, and glial cell proliferation, further signaling processes such as chemokine receptor binding, cytokine activity, glycosaminoglycan binding, and G‐protein–coupled receptor binding, as well as growth plasma levels and metabolic activities. Pathways significantly suppressed by means of reduced expression of the corresponding proteins in sDS included cellular structure and organization, cell proliferation, cell signaling and communication, and transcription and translation (Figure 2C,D, Table S4).

**FIGURE 3:**
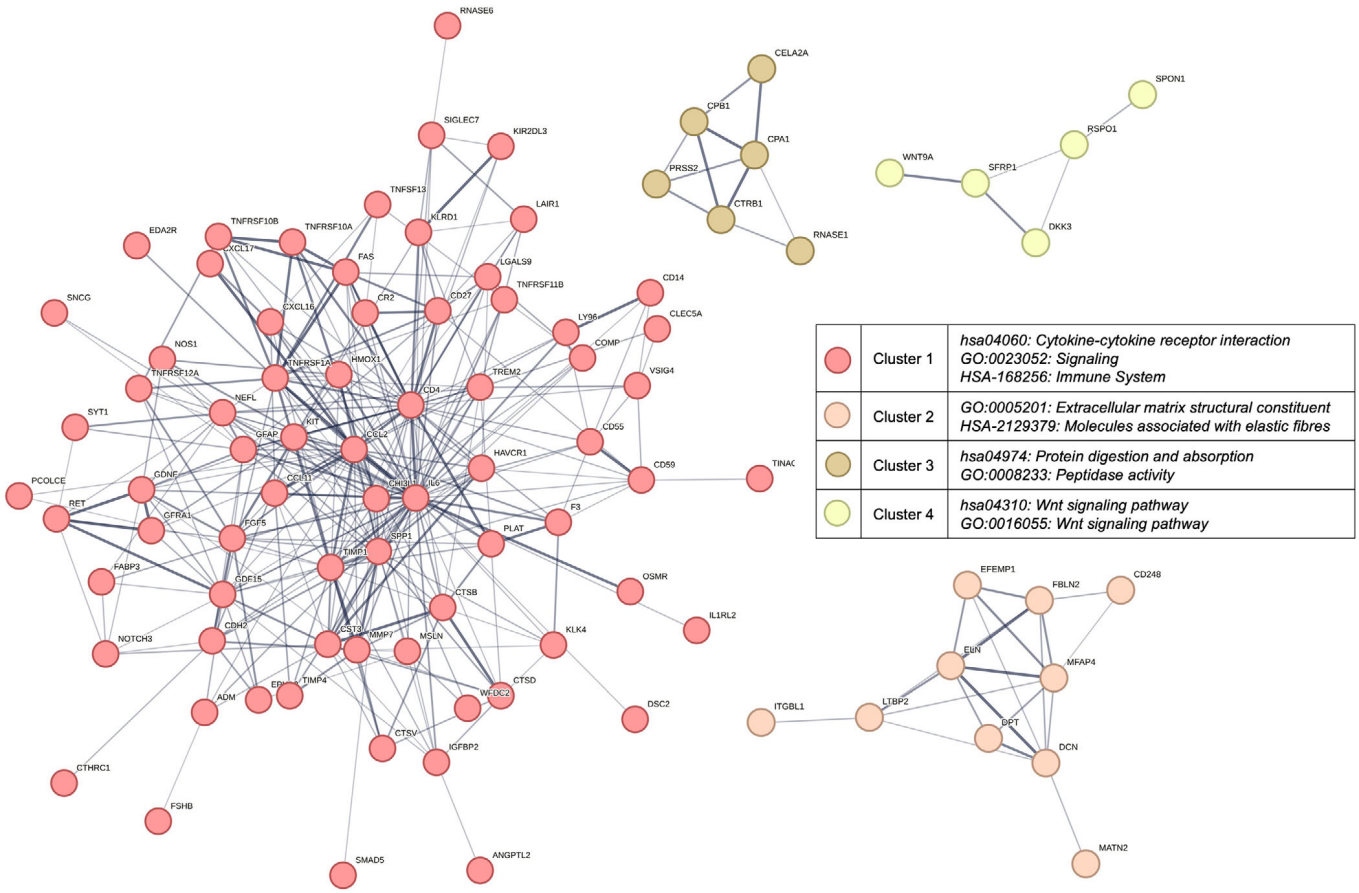
Top four clusters using the Markov–Cluster algorithm from PPI STRING network analysis of DEP between symptomatic and asymptomatic adults with DS with most significant enriched terms annotated in the legend suggesting differences in immune system signaling (cluster 1), the makeup of the extracellular matrix (cluster 2), enzymatic protein digestion (cluster 3), and Wnt signaling (cluster 4). Each node represents one DEP while each edge represents functional interaction between two DEP with the thickness of the edge increasing with the confidence of the proposed shared function. DEP, differentially expressed proteins; DS, Down syndrome; PPI, protein–protein interactions.

Looking at PPI networks within DS, DEP between symptomatic and asymptomatic adults were clustered into 13 functional subnetworks (Figure 3) with the largest consisting of 72 proteins showing enrichment for terms including cytokine–cytokine receptor interaction (hsa04060), signaling (GO:0023052), death receptor activity (GO:0005035), and immune system (HSA‐168256). The second largest cluster made up of 10 DEP was associated with extracellular matrix structural constituents (GO:0005201, GO:0031012) and molecules associated with elastic fibers (HSA‐2129379). Another cluster counting six DEP exhibited functional association of PPI with enzymatic activity such as protein digestion and absorption (hsa04974) and peptidase (GO:0008233) as well as hydrolase (GO:0016787) activity. Finally, the fourth largest cluster consisted of five DEP associated with the Wnt signaling pathway (hsa04310, GO:0016055). All remaining clusters can be found in the supplements (Table S5).

**FIGURE 4:**
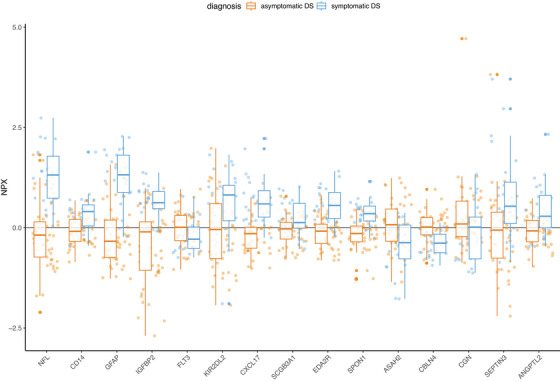
Box plots of individual NPX levels from symptomatic and asymptomatic adults with Down syndrome for all 15 proteins received from LASSO feature selection. LASSO, least absolute shrinkage and selection operator; NPX, normalized protein expression.

LASSO feature selection (balanced accuracy: 0.9, sensitivity: 0.8, specificity: 1, positive predictive value: 1, negative predictive value: 0.7143) investigated all proteins measured regarding their potential for predicting the outcome of sDS versus aDS and selected 15 non‐zero coefficients, with, apart from NFL and GFAP, 13 further potential markers spanning areas of immune response, neurotransmission, and receptor signaling (Figure 4, Table S6). Eleven proteins contributed positively toward the probability of sDS, while the remaining four exhibited a relevant negative relationship with the outcome of sDS versus aDS (Figure S2).

**FIGURE 5:**
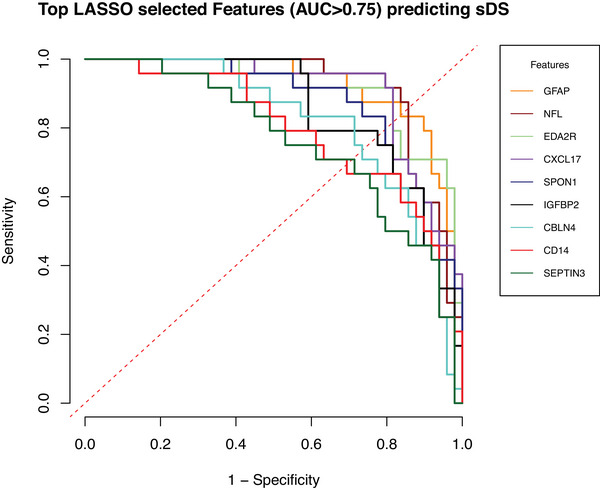
All nine proteins received from feature selection analysis with their corresponding AUC for diagnostic performance of differentiating between symptomatic and asymptomatic DS with corresponding ROC curves and an AUC over 0.75. AUC, area under the curve; DS, Down syndrome; ROC, receiver operating characteristics.

We assessed the diagnostic performance of all 15 proteins with ROC analysis which revealed an AUC above 0.75 for nine of the LASSO‐selected features (Table 2, Figure 5), specifically NFL, GFAP, ectodysplasin A2 receptor (EDA2R), C‐X‐C motif chemokine 17 (CXCL17) and cluster of differentiation 14 (CD14), insulin‐like growth factor binding protein‐2 (IGFBP2), spondin‐1 (SPON1), cerebellin 4 (CBLN4), and neuronal‐specific septin‐3 (SEPTIN3).

**TABLE 2:** All 15 proteins received from feature selection analysis with their corresponding AUC for diagnostic performance of differentiating between symptomatic and asymptomatic DS.

Feature	AUC
GFAP	0.925
NFL	0.916
EDA2R	0.91
CXCL17	0.904
SPON1	0.872
IGFBP2	0.855
CBLN4	0.815
CD14	0.792
SEPTIN3	0.759
ANGPTL2	0.732
KIR2DL2	0.72
ASAH2	0.711
SCGB3A1	0.71
FLT3	0.654
CGN	0.617

Abbreviation: AUC, area under the curve; DS, Down syndrome.

## 4 DISCUSSION

This cross‐sectional study investigated proteomic profiles in adults with and without DS leveraging the Olink Explore 3072.

For 253 DEP between DS and HC, enrichment analysis reported coordinated up‐regulation in protein levels for immune activity, cell and cytokine signaling, and neuronal growth while proteins for DNA processing, cell cycle, and biosynthesis were significantly down‐regulated in DS. Eleven DEP originated from chr21, with reported involvement in immune response regulation, cellular adhesion, and tissue remodeling.^29^, ^38^, ^39^ Analyzing PPI, large clusters related to enhanced processes such as regulation of leucocyte proliferation, cytokine production, synaptic function, and lipid metabolism in DS.

This is in line with observations of a dysregulated and highly active immune system throughout the lifespan of DS,^40^, ^41^ resulting in higher susceptibility for autoimmune diseases,^42^, ^43^ further corroborated by reports of increased cytokine blood levels in children and adults with DS^40^, ^42^, ^44^ and a higher chemokine and cytokine response from monocyte‐derived dendritic cells of DS patients.^44^ Also, a study with SomaScan in blood reported similar pathway enrichments of inflammatory response, immune control, and regulation of neurogenesis between DS and HC.^29^

DS is considered a primary interferonopathy given that increased cytokine signaling is likely due to the fact that four of six interferon (IFN) receptors are encoded on chr21.^43^, ^45^ We, too, found correlates of enhanced signaling in DS with increased levels of IFNAR1, IL10RB, originating chr21, as well as IFNGR1, ILR1, IL4R, and IL20RB. Since chemokines and cytokines play a vital role by regulating cell constitution, migration, and balancing pro‐ and anti‐inflammatory processes,^29^, ^46^ dysregulations could lead to progressive neuroinflammation contributing to DS‐AD.^26^, ^27^ Further, dysregulated cell adhesion molecules pathways associated with chr21 in DS versus HC could affect cellular interactions and tissue remodeling. Junctional adhesion molecule 2 (JAM2) is involved in endothelial tight junction formation,^38^ has been associated with AD,^47^ and could potentially affect the blood–brain barrier permeability in DS‐AD. Elevated levels of neural cell adhesion molecule 2 (NCAM2) and Collagen Type XVIII Alpha 1 Chain (COL18A1) arguably contribute to the intellectual disability phenotype^39^ and decreased risk for solid tumors^48^ in DS, respectively. However, their impact within DS‐AD remains unclear. Increased levels of cystatin B (CSTB) though have been found in DS‐AD brains, potentially affecting amyloid metabolism and neuroinflammatory processes via modulation of cysteine cathepsins.^49^

Pathways of neuronal development and regeneration were also significantly enriched in DS. Studies in induced pluripotent DS stem cells suggest abnormal neuronal differentiation affecting neuronal architecture and density as well as a number and length of neurites, and differentiation into GFAP‐positive cells, thereby promoting a shift from the neuronal to the astroglial and oligodendroglial phenotype,^50^, ^51^ which could facilitate later developments of neuroinflammation and AD pathology.

Pathway analyses in blood from euploid sAD versus HC leveraging OLINK reported changes in signal transduction, apoptosis and inflammatory pathways, cell proliferation, and monocyte chemotaxis,^52^ as well as positive regulation of protein kinase B signaling, growth factor binding, and cytokine–cytokine receptor interaction.^53^ While the latter indicates some shared proteomic alterations with our analysis, most of the reported pathways suggest distinct differences in the proteomic dynamics between sAD and DS‐AD, underlining the value of DS as a unique model for AD.

Subsequently, we identified 142 DEP between sDS and aDS, none originating from chr21, suggesting proteomic differences not directly resulting from increased gene dosage of chr21, but rather of downstream effects.

The major findings in enrichment analysis within DS concerned dysregulation of immune function and cell signaling, tissue differentiation, extracellular constituents, and metabolic activity, which was mirrored in PPI analysis.

Dysregulated cytokine signaling, antigen presentation, and signaling involving G‐protein coupled receptors suggest imbalances in immune cell communication and glial cell proliferation in sDS, in line with an overactive immune environment by further amplifying inflammatory responses, possibly resulting from AD pathology.^54^ Studies report dynamic cytokine profiles along the DS‐AD spectrum, resembling an early and a late neuroinflammatory period, with early peaks of upregulated pro‐inflammatory markers like interleukin (IL)‐6, IL‐1β, IL‐15, and macrophage‐derived chemokine (MDC) around age 20.^26^ Likewise, elevated plasma levels of sTREM2 have been observed in young DS.^55^ Late inflammatory processes, however, have been characterized as persistent activation of increasingly dystrophic microglia and decreased immune markers like IL‐10, IL‐12, tumor necrosis factor‐alpha (TNFα), and IFNγ, while levels of IL‐6, IL‐8, IL‐15, and monocyte chemoattractant protein‐1 (MCP‐1) increased.^26^ A phenotype of microglial activation characterized by elevation of CD86, IL‐10, TNFα, IL‐1β, IL‐6, and decrease in IL‐12, already in asymptomatic patients below age 40, has been reported in DS brain tissue and shows further exacerbation with age^27^ while investigations in blood in DS with or without AD showed elevated levels of proinflammatory markers with a combined measure of amyloid‐β and inflammatory agents predicting future cognitive decline the following 24 months.^56^

The downregulation of several proteins in sDS relates to considerable cell cycle dysregulation suggestive of altered cellular growth, repair mechanisms, cell stability, and chromosomal segregation. Alterations in cell cycle and proliferation potency have been reported in trisomic cells,^57^, ^58^ and a DS mouse model showed decelerated cell proliferation with increasing age^59^ while ribosomal biogenesis exhibited dysregulation in older compared to younger DS individuals.^60^ Interestingly, a study reported that increases in APP expression could inhibit cell proliferation by regulating global gene expression.^61^ Since the onset of symptomatic DS‐AD is inevitably intertwined with accumulating AD pathology and therefore increasing age, our findings support potential molecular changes during the life span of DS possibly drive aging processes and benefit AD pathophysiology.

Applying LASSO, we identified 15 proteins for the distinction between sDS and aDS, 9 of which were identified as DEP and exhibited an AUC above 0.75.

NfL blood levels increase early in DS‐AD, with baseline concentrations predicting dementia status and preceding amyloid PET changes by up to 10 years^14^, ^15^, ^62^, ^63^ while GFAP, rises significantly in prodromal and symptomatic DS‐AD, correlating well with imaging measures of AD (‐related) pathology.^24^ We identified both as relevant features for sDS, confirming strong diagnostic performance (AUC > 0.9).^15^, ^24^

EDA2R is enriched in reactive astrocytes^64^, ^65^ and higher levels have been associated with smaller grey matter volume and impaired fluid cognitive ability in euploids.^66^ In our sDS cohort, EDA2R overexpression could therefore contribute to cognitive decline via chronic reactive astrogliosis.

The astrocytic signaling protein IGFBP2 is increased in AD,^67^ associated with clinical diagnosis of mild cognitive impairment or dementia, AD‐like brain atrophy, and CSF tau levels,^68^, ^69^ supporting our finding of significantly increased blood levels in sDS.

Soluble CD14 has been shown to enhance immune response to lipopolysaccharides in human euploid cells^70^ and is increased in sDS, suggesting a further exacerbation of the augmented reaction to lipopolysaccharides reported in DS by CD14‐positive‐monocytes‐derived cells.^44^ We, too, found it significantly increased in sDS, potentially contributing to dysregulated immune‐related pathways, exhibiting a moderate association with disease status.

While CXCL17 is mostly associated with chemotaxis in mucosal tissue,^71^ a mouse model study found increased cerebellar expression of CXCL17 upon stimulation by peripheral inflammatory agents,^72^ which could explain the elevated levels and good discriminative power within our analysis.

SPON1, significantly increased in sDS, is reported to prohibit enzymatic release of amyloid by binding to APP,^73^ thereby possibly mediating cognitive decline and structural brain changes in AD,^74^, ^75^ and increase in CSF in autosomal‐dominant AD, as early as 30 years prior symptom onset.^76^

Finally, synaptic proteins SEPTIN3 and CBLN4 were increased and decreased, respectively, in sDS, hinting at compensatory processes ameliorating AD‐related synaptic dysfunction. Indeed, disorganization and accumulation of SEPTIN3 has been associated with complement‐dependent synapse loss in AD^77^ and its gene polymorphisms are considered relevant for AD pathology susceptibility.^78^ Conversely, its elevated levels in sDS could also hint at increased release from perishing synapses rather than overexpression, as proposed with beta‐synuclein.^25^ Meanwhile, CBLN4, pivotal for synaptic formation and maintenance, has been suggested as a treatment target of AD due to its amelioration of amyloid‐related synaptic dysfunction.^79^ Therefore, reduced expression levels in sDS might be contributing to synaptic dysfunction.

Our study has certain limitations. Our sample size of 73 adults with DS and 15 HC is rather small, warranting further analyses with larger cohorts to validate present findings. Secondly, our cross‐sectional sample allows only limited interpretation of the potential biomarkers uncovered; however, DS‐AD arguably allows for a pseudo‐longitudinal interpretation similar to autosomal‐dominant AD^80^ due to the genetically determined course of pathology.^81^ Further, lack of comprehensive data for amyloid, tau, and markers of neurodegeneration for the majority of the cohort prohibited us from further differentiating potential biomarkers according to neuropathological status. Finally, LASSO analysis was not adjusted for age due to its high correlation with diagnosis (Pearson *r* = 0.74) and strong potential for solely predicting diagnosis, indicating a highly significant association (logistic regression estimate = 0.225, *p* < 2e‐16, Akaike information criterion [AIC] = 124,231). While this approach avoids conflating age‐related effects with the outcome of diagnosis, it limits our ability to fully disentangle their independent contributions, which we plan to address in future analyses.

In conclusion, this work confirmed NFL and GFAP as powerful blood markers for DS‐AD and further identified several novel potential biomarkers with considerable power for diagnosing DS‐AD. Motivated by the need for the implementation of an easily accessible and well‐tolerated alternative to lumbar punctures and imaging protocols, the identified candidates warrant further investigation.

## CONFLICT OF INTEREST STATEMENT

J.F. reports receiving personal fees for service on the advisory boards, adjudication committees or speaker honoraria from AC Immune, Adamed, Alzheon, Biogen, Eisai, Esteve, Fujirebio, Ionis, Laboratorios Carnot, Life Molecular Imaging, Lilly, Lundbeck, Perha, Roche and outside the submitted work as well as holding a patent for markers of synaptopathy in neurodegenerative disease (licensed to Adx, EPI8382175.0). G.H. serves as a consultant for Abbvie, Alzprotect, Amylyx, Aprinoia, Asceneuron, Bayer, Bial, Biogen, Biohaven, Epidarex, Ferrer, Kyowa Kirin, Lundbeck, Novartis, Retrotope, Roche, Sanofi, Servier, Takeda, Teva, UCB; received honoraria for scientific presentations from Abbvie, Bayer, Bial, Biogen, Bristol Myers Squibb, Esteve, Kyowa Kirin, Pfizer, Roche, Teva, UCB, Zambon; holds patents (US 10,918,628 B2; EP 17 787 904.6‐1109/3 525 788); received publication royalties from Academic Press, Kohlhammer, and Thieme. J.L. reports speaker fees from Bayer Vital, Biogen, EISAI, TEVA, Zambon, Esteve, Merck and Roche, consulting fees from Axon Neuroscience, EISAI, and Biogen, author fees from Thieme medical publishers and W. Kohlhammer GmbH medical publishers and is inventor in a patent “Oral Phenylbutyrate for Treatment of Human 4‐Repeat Tauopathies” (PCT/EP2024/053388) filed by LMU Munich. In addition, he reports compensation for serving as chief medical officer for MODAG GmbH, is beneficiary of the phantom share program of MODAG GmbH and is inventor in a patent “Pharmaceutical Composition and Methods of Use” (EP 22 159 408.8) filed by MODAG GmbH, all activities outside the submitted work. J.M.G., S.M.H., S.K., C.J.M., G.N., S.V.L., F.J.M.M., A.P., C.P., R.R.Z., A.S., K.S., E.W., and O.W. report no conflict of interest. Author disclosures are available in the supporting information.

## CONSENT STATEMENT

All DS patients included in this analysis were recruited through our AD21 cohort study investigating AD in adults with DS; each individual or their respective legal proxy provided informed written consent prior to inclusion. Blood samples of healthy controls without the diagnosis of DS from our biobank were also obtained after providing written consent. Both studies are supervised and approved by the LMU ethics committee (DS: #17‐126, HC: #20‐0997) and conducted in accordance with the Declaration of Helsinki.

## Supporting information



ICMJE Disclosure Form

Supporting Information
