# Supplementary material for: Exploratory analysis of the proteomic profile in plasma in adults with Down syndrome in the context of Alzheimer's disease
Source: Alzheimers Dement. 2025 Mar 20;21(3):e70040. doi: 10.1002/alz.70040 (PMC11923571; doi:10.1002/alz.70040)

SUPPLEMENTARY INFORMATION

Table S 1: Specifics regarding AD21 study design.

Table S 2: Results from KEGG & GO pathway enrichment analysis in Down Syndrome (DS) vs. healthy euploid controls (HC).

Table S 3: Results from STRING protein-protein-interaction analysis in DS vs. HC.

Table S 4: Results from KEGG & GO pathway enrichment analysis in symptomatic vs. asymptomatic DS.

Table S 5: Results from STRING protein-protein-interaction analysis in symptomatic vs. asymptomatic DS.

Table S 6: Results from LASSO feature selection.

Figure S 1: PPI STRING network of DEP between DS and HC colored according to MCL clustering.

Figure S 2: Visualization of LASSO Feature Selection in symptomatic vs. asymptomatic DS.

Table S 1: *Specifics regarding AD21 study design. The comprehensive study protocol can be requested through the corresponding author upon reasonable request.*

Study design	Monocentric prospective cohort study
Intended Follow-Up duration	5 years
Study population	Adults with Down Syndrome (DS)
Main Objective	To identify valid clinical, biological and neuroimaging biomarkers to monitor the development of Alzheimer's disease in patients with DS
Investigation modalities	*Annually:*<br>Neurological examinations including appropriate neurocognitive testing<br><br>*Additionally, if possible and feasible:*<br>Blood-, cerebral fluid- and fibroblast samples as well as MRI- & PET-scans
Study criteria considerations	*Inclusion criteria:*<br>Individuals of age and with diagnosis of DS with the ability to consent themselves or by proxy and with absence of any other structural diseases of the central nervous system<br><br>*Exclusion criteria:*<br>Children with DS, or individuals with DS with an existing diagnosis of partial trisomy 21 lacking an APP triplication or a known familial AD / other types of familial neurodegenerative disease as well as failure to consent themselves / by proxy

Table S 2: *Results from KEGG & GO pathway enrichment analysis* in *Down Syndrome (DS) vs. healthy euploid controls (HC).*

Pathway	ES	NES	P Value	FDR P Value	Enriched Proteins
**KEGG**					
KEGG_CELL_ADHESION_MOLECULES_CAMS	0.4598	1.8251	0.0003	0.0161	CDH15/NLGN2/JAM2/SELE/CNTNAP2/CD276/HLA-E/NCAM2/PDCD1LG2/CDH1/ITGAL/CDH2/VCAN/PDCD1/ICOSLG/SDC1/ICAM3/ITGB7/SIGLEC1/ICAM2/CTLA4/PTPRF/CDH3/HLA-DRA/NECTIN2/PTPRM/NCAM1/NLGN1/NRXN3/L1CAM/CNTN2/ITGB2/VCAM1
KEGG_CYTOKINE_CYTOKINE_RECEPTOR_INTERACTION	0.4038	1.8326	0.0000	0.0004	IL19/IL22/IL10RB/PRL/IL4/CXCL10/CCL17/GH1/TNFRSF19/CD27/IL10/CXCL9/FLT4/IL17A/IL1R1/INHBB/IL11/CSF3/EDA2R/CCL15/TGFBR1/LEP/EPO/TNFRSF8/TNFRSF17/PDGFRA/TNFRSF13B/IL4R/CSF1/ACVRL1/TSLP/VEGFA/TNFRSF1A/TNFRSF9/IFNGR2/CSF2/TNFRSF1B/TNFRSF11A/TGFBR2/LIFR/CXCL13/CCL28/TNFRSF12A/TNF/VEGFB/OSM/FAS/TNFRSF6B/TNFRSF4/CCL20/RELT/CCL25/CCL13/IL17RB/CSF1R/IL20RA/CSF2RB/CD70/XCL1/CXCL11/IL20RB/CXCL1/IL5/MET/TNFRSF11B/CCL21/IFNGR1/IFNAR1
**GO**					
GOBP_REGULATION_OF_SUPEROXIDE_ANION_GENERATION	0.7731	1.9772	0.0008	0.0414	MAPT/CLEC7A/CD177/SOD1
GOBP_REGULATION_OF_RECEPTOR_BINDING	0.7819	1.9579	0.0011	0.0488	ANXA2/IL10/NRP1/B2M/PTPRF
GOBP_NEURON_PROJECTION_REGENERATION	0.5634	1.9002	0.0009	0.0429	OMG/FKBP1B/NEFL/KLK8/MTR/PTN/THY1/KLF4/APOD/FOLR1/TNR/GFAP/KIAA0319/PTPRF/SPP1/EPHA4
GOBP_ACUTE_INFLAMMATORY_RESPONSE	0.4967	1.8293	0.0012	0.0500	IL22/IL4/ADRA2A/MBL2/HLA-E/REG3A/APOA2/EPO/LBP/TNFRSF11A/NPY/TNF/OSM/IL20RB/ASS1/ZP3/B4GALT1/ADAM8/CD163/ITIH4/APCS
GOMF_MOLECULAR_TRANSDUCER_ACTIVITY	0.3599	1.7407	0.0000	0.0009	GFRAL/NLGN2/CLEC7A/IL10RB/FOLR3/KIR3DL1/SELE/CD300LG/ADRA2A/FCGR2A/TNFRSF19/CD27/FGFR4/FLT4/CLEC4D/CD300A/NRP1/CD300C/CD300LF/ROR1/HTR1A/ADGRV1/IL1R1/REG3A/FCRL6/ADGRG1/TGFBR3/FCAMR/ADGRD1/CRHR1/EDA2R/RTBDN/ITGAL/PTPRN2/SLAMF1/FCRL5/CD74/TGFBR1/TNFRSF8/TNFRSF17/LAG3/FOLR1/RGMB/PDGFRA/TNFRSF13B/IL4R/GFRA3/ACVRL1/TNFRSF1A/TNFRSF9/IFNGR2/TNFRSF1B/TNFRSF11A/TGFBR2/CD160/PTPRR/EFEMP1/EPHA2/EPHB4/LIFR/CD300E/ITGB7/PLAUR/FOLR2/NPY/NOTCH3/CD33/FAS/TNFRSF6B/KIR2DL3/BCAM/PTPRF/REG4/TNFRSF4/AXL/LY96/UNC5D/IL17RB/MILR1/CSF1R/CSPG4/TIE1/SIGLEC8/GFRA1/IL20RA/FCRLB/REG1A/EPHA4/CSF2RB/EPHA1/THBD/IL20RB/GRIN2B/CD14/MET/HLA-DRA/MARCO/TNFRSF11B/CD244/SLAMF8/IFNGR1/IFNAR1/COLEC12/NECTIN2/ADGRF5/PTPRM/IL5RA/CD79B/PKD1/NLGN1/CD5/FGFR2/IFNLR1/EFNA4/RTN4R/ESR1/CELSR2/NRXN3/L1CAM/REG1B/CRIM1/ITGB2/TNFRSF10A/IL2RA/KLRK1/IL1RAP/IL7R/FCER1A/ADGRE5/IL15RA/EPHB6/MERTK/SORCS2/CLEC1A/PTPRZ1/DCC
GOMF_GROWTH_FACTOR_BINDING	0.4139	1.6377	0.0010	0.0458	IGFBP1/CCN2/FGFR4/SPINT3/FLT4/CCN5/NRP1/IGFBP4/IGFBP6/IL1R1/WFIKKN2/TGFBR3/TGFBR1/PDGFRA/S100A13/LTBP2/ACVRL1/IGFBP7/TGFBR2/COL3A1/EPHA2/LIFR/CXCL13/CCN3
GOCC_CELL_SURFACE	0.3213	1.5924	0.0000	0.0039	GFRAL/TDGF1/TNN/NLGN2/CLEC7A/ANXA2/PRND/FOLR3/JAM2/SELE/CXCL10/CD27/LY6D/MBL2/CNTNAP2/LAMP3/CXCL9/CD276/CLEC4D/IL17A/HLA-E/ROR1/THY1/ADGRV1/LAYN/IL1R1/FCRL6/PDCD1LG2/BST2/TGFBR3/RTBDN/ITGAL/ULBP2/SLAMF1/IGLC2/MSR1/FCRL5/CD74/TGFBR1/EPO/B2M/SCARA5/CDH2/LAG3/FOLR1/SLAMF7/PDGFRA/IL4R/ACE2/ASGR2/CDH17/FOLH1/GFRA3/ACVRL1/VEGFA/TNFRSF1A/LBP/TNFRSF9/TNFRSF11A/TGFBR2/PDCD1/ICOSLG/HAVCR2/ITGBL1/CA4/EPHA2/SFRP4/LIFR/SDC1/ITGB7/PLAUR/FOLR2/TNR/NOTCH3/ADA/LRFN2/TNF/APOH/CTLA4/CD33/FAS/BCAM/TNFRSF4/AXL/LRP2/UNC5D/SLITRK6/SHH/CSF1R/CSPG4/ERMAP/ISLR2/GFRA1/EPHA4/CSF2RB/ANGPTL3/THBD/CD99L2/GRIN2B/MSLN/CD38/CD14/B4GALT1/CD59/CD109/MET/ADAM8/HLA-DRA/CD163/BTN3A2/MXRA8/CD244/SLAMF8/TFPI/CLU/LY9/NECTIN2/ADGRF5/NCAM1/IL5RA/CD79B/PKD1/NLGN1/APOA4/PAM/CD5/WIF1/FGFR2/BMP10/CLEC4C/RTN4R/ACHE/CEACAM6/L1CAM/PDGFC/CNTN2/BTNL10/MFGE8/ITGB2/BTN2A1/VCAM1/FURIN/TNFRSF10A/ACE/ASGR1/IL2RA/KLRK1/IL7R/CD200/FCER1A/TGFA/CD209/CD55/IL15RA
GOCC_EXTERNAL_ENCAPSULATING_STRUCTURE	0.3350	1.5745	0.0002	0.0168	TNN/ANXA2/COCH/APLP1/GH1/CCN2/MBL2/CSTB/MMP1/MMP3/MYOC/CCN5/PZP/THBS2/PI3/FBLN2/LGALS1/COL6A3/SPON2/VWC2/TGFBR3/ADAMTS1/RTBDN/MFAP5/OGN/CDH2/CHI3L1/TGM2/VIT/VCAN/LTBP2/VEGFA/ENTPD2/MMP12/IGFBP7/FGL1/COL3A1/TNC/SSC5D/CRELD1/EFEMP1/HMCN2/HSP90B1/NID1/OMD/TNR/GDF15/COL18A1/CCN3/APOH/MATN2/OPTC/MST1/BCAM/ADAMTSL2/TIMP2/PCOLCE/CTHRC1/SHH/APOE/CSPG4/NCAN/ANGPTL2/ANGPTL3/BCAN/COL28A1/CHAD/ZP3/DPT/PTX3/ADAMTS4/HSPG2/AGRN/COL5A1/ASPN/TNFRSF11B/COLEC12/CLU/ANGPTL1/ITIH4/APCS/DMP1/THBS4/CTSZ/NCAM1/APOA4/SMOC2/SMOC1/RARRES2/FGFR2/IMPG1/LTBP3/ACHE/MMP10/ANGPTL4/FGFBP3/L1CAM/MATN3/MFGE8/TIMP1/ORM1/SOST/MEPE/FLRT2/ANGPT2/ADAMTSL4/TNXB/LGALS9/COL15A1/PTPRZ1/VASN/GPC1/COL1A1/CTSC/COMP/SEMA7A/DCN/PRELP/LRIG3/CTSB/LEFTY2/CD248/PHOSPHO1/LRRN1/LGALS3BP/TGFBI/MMP8/LRIG1/MDK/SPARCL1/CBLN1/SERPINH1/FGA/LGALS4/VWA1/PSAP
GOCC_EXTERNAL_SIDE_OF_PLASMA_MEMBRANE	0.3366	1.5478	0.0010	0.0479	GFRAL/CLEC7A/PRND/FOLR3/SELE/CXCL10/CD27/CXCL9/CD276/CLEC4D/IL17A/HLA-E/THY1/IL1R1/FCRL6/PDCD1LG2/TGFBR3/RTBDN/ITGAL/ULBP2/SLAMF1/IGLC2/MSR1/CD74/B2M/SCARA5/LAG3/FOLR1/SLAMF7/PDGFRA/IL4R/ASGR2/GFRA3/TNFRSF9/TNFRSF11A/TGFBR2/PDCD1/ICOSLG/CA4/LIFR/SDC1/FOLR2/ADA/TNF/CTLA4/CD33/FAS/BCAM/TNFRSF4/LRP2/ERMAP/GFRA1/CSF2RB/THBD/CD14/B4GALT1/CD59/CD163/BTN3A2/CD244/LY9/NCAM1/IL5RA/CD79B/NLGN1/CD5/CLEC4C/RTN4R/BTNL10/MFGE8/ITGB2/BTN2A1/VCAM1/ACE/ASGR1/IL2RA/KLRK1/IL7R/CD209
GOMF_SIGNALING_RECEPTOR_REGULATOR_ACTIVITY	0.3218	1.5175	0.0004	0.0309	TFF1/IL19/IL22/TDGF1/PRL/NENF/GAST/DKK4/IL4/PSPN/CXCL10/CCL17/GH1/CCN2/IL10/CXCL9/FAM3D/SPINT3/MIA/IL17A/IL17C/PTN/FAM3B/NBL1/INHBB/IL11/WFIKKN2/CSF3/DAND5/RETN/SLURP1/CCL15/LEP/EPO/OGN/C1QTNF9/PPY/PGF/DKK3/CSF1/NRTN/TSLP/TAFA5/VEGFA/CHGB/CSF2/EFEMP1/GCG/NPY/CXCL13/CCL28/GDF15/TNF/CCN3/VEGFB/FST/OSM/FGF19/FSHB/IL36G/ADA2/FAM3C/CCL20/CCL25/CCL13/SHH/GPNMB/AGRP/IL18BP/SPP1/REG1A/CD70/XCL1/CXCL11/ANGPTL3/STC1/ZP3/CXCL1/IL5/PENK/NPPC/CLEC11A/SEMA4C/TNFRSF11B/CCL21/NTF3
GOBP_NEURON_DEVELOPMENT	0.3193	1.5073	0.0005	0.0328	MAPT/OMG/TNN/NLGN2/WASL/BLOC1S2/DNM3/APLP1/FKBP1B/NEFL/UGDH/KLK8/RAP1A/GNGT1/CNTNAP2/MTR/MYOC/NRP1/ROR1/PTN/NCAM2/THY1/ADGRV1/CDKL5/INPP5J/NBL1/SLITRK1/SEZ6/CDH1/KLF4/STX1B/LYPLA2/ELAVL4/LEP/PLA2G10/EPO/B2M/C

					DH2/APOD/FOLR1/CHL1/SOD1/NRTN/GFRA3/DSCAM/VEGFA/SYT1/EPHA2/EPHB4/TNR/NPY/NOTCH3/CDH23/GFAP/KIAA0319/PTPRF/CTHRC1/LRP2/AMIGO1/SLC9A3R1/UNC5D/SLITRK6/SHH/APOE/CSF1R/ISLR2/SPP1/GFRA1/EPHA4/EPHA1
GOCC_INTRINSIC_COMPONENT_OF_PLASMA_MEMBRANE	0.2828	1.4229	0.0006	0.0342	NLGN2/IL10RB/PRND/CD177/FOLR3/JAM2/CXADR/KIR3DL1/SELE/ADRA2A/FCGR2A/CD27/CDHR2/CNTNAP2/FGFR4/FLT4/KCNIP4/TSPAN7/NRP1/CD300C/ROR1/HTR1A/THY1/KIRREL1/IL1R1/SLITRK1/FCRL6/VWC2/ADGRG1/TGFBR3/ADGRD1/CRHR1/EDA2R/PCDHB15/RTBDN/ITGAL/PTPRN2/ULBP2/MSR1/FCRL5/TGFBR1/DLL1/PODXL2/SCARA5/CDH2/TGM2/VSIG2/FOLR1/RGMB/ATP2B4/CD82/PDGFRA/TNFRSF13B/IL4R/FOLH1/ACVRL1/DSCAM/DPP10/TNFRSF1A/LRRC38/TNFRSF9/IFNGR2/CSF2/PILRB/TGFBR2/C9/CD160/ITGBL1/CA4/EPHA2/EPHB4/TMPRSS11D/LIFR/SDC1/ICAM3/ITGB7/PLAUR/FOLR2/ICAM2/SLC39A5/KIR2DS4/LRFN2/TNF/CTLA4/NCR1/CD33/KIR2DL3/BCAM/TREH/PTPRF/TNFRSF4/AXL/RNF43/LY96/XG/SLITRK6/GPNMB/IL17RB/MILR1/CSF1R/SCN4B/CSPG4/TIE1/IL20RA/FCRLB/EPHA4/CSF2RB/EPHA1/CD70/ADAM22/THBD/IL20RB/ART3/GRIN2B/CD14/CD59/MET/ADAM8/C1QTNF1/HLA-DRA/MARCO/CD163/SEMA4C
GOBP_POSITIVE_REGULATION_OF_CELLULAR_BIOSYNTHETIC_PROCESS	-0.3003	-1.4501	0.0011	0.0488	ANXA1/EIF4G1/CX3CL1/STK11/DDX58/EDN1/NPM1/RFC4/LIF/AP3B1/NFIC/TNFSF11/AXIN1/TRIM21/PAGR1/CREB3/CREBZF/DAB2/ITGA6/EREG/PDGFRB/EDF1/RGCC/CDK1/TET2/CRX/TRIM5/CEBPB/FUS/TRIAP1/PITHD1/NFYA/CHEK2/NFE2/FMR1/TP53/DDAH1/ERCC1/MYDGF/HDGF/CCL19/CCAR2/PRKCQ/HIF1A/TF/ENO1/TRIM24/TP73/PPP1R12A/IFNL1/ABL1/YY1/IKBKG/PF4/NGRN/REST/TLR3/F2R/ATF2/TEF/FOSB/TARBP2/SIRT1/HNRNPK/GLYR1/PARP1/EGF/CDK5RAP3/ZBTB16/SKAP1/PTGES2/SMARCA2/FOXJ3/WWP2/CCN1/EPCAM/CAPN3/YES1/FOXO3/NAMPT/RET/ARID4B/LARP1/LYAR/GFER/ING1/TP53BP1/TNIP1/NFATC1/CCL5/ZPR1/FOXO1/TRIM25/VIM/WAS/PDGFB/NEK7/RUVBL1/SMAD1/SRC/CENPJ/SMAD3/PKD2/GAL/MAP3K5/CALCOCO1/MAVS/EIF2AK3/NFAT5
GOBP_POSITIVE_REGULATION_OF_RNA_METABOLIC_PROCESS	-0.3176	-1.4996	0.0007	0.0395	DDX58/EDN1/NPM1/LIF/AP3B1/NFIC/ERN1/TNFSF11/AXIN1/TRIM21/PAGR1/CREB3/CREBZF/DAB2/ITGA6/EDF1/RGCC/TET2/CRX/TRIM5/CEBPB/FUS/TRIAP1/PITHD1/NFYA/CHEK2/NFE2/TP53/ERCC1/MYDGF/HDGF/CCAR2/HIF1A/TF/TRIM24/TP73/PPP1R12A/IFNL1/ABL1/YY1/IKBKG/PF4/REST/TLR3/F2R/ATF2/TEF/FOSB/SIRT1/HNRNPK/GLYR1/PARP1/EGF/CDK5RAP3/ZBTB16/GIGYF2/DXO/SKAP1/PTGES2/SMARCA2/FOXJ3/WWP2/CCN1/EPCAM/CAPN3/YES1/FOXO3/NAMPT/RET/ARID4B/LYAR/ING1/TP53BP1/TNIP1/NFATC1/ZPR1/FOXO1/TRIM25/WAS/PDGFB/RUVBL1/SMAD1/SRC/CENPJ/SMAD3/PKD2/GAL/MAP3K5/CALCOCO1/MAVS/EIF2AK3/NFAT5
GOMF_KINASE_BINDING	-0.3406	-1.5510	0.0008	0.0418	CD3E/LATS1/SLC12A2/XIAP/NPM1/DOK2/AXIN1/DUSP3/CCND2/TRIM21/DNM1/PDGFRB/WARS1/RGCC/ITGAX/TRIM5/TFRC/CEBPB/PFKFB2/CHEK2/TOP2B/TP53/CD6/BANK1/CRKL/SH2B3/HIF1A/TPR/ACTA2/TPRKB/TP73/GP6/RELB/CDKN1A/PPP1R12A/PRKAR1A/ABL1/CD226/TAX1BP1/RNF41/ATF2/SDC4/SIRT1/DLG4/PPP1R12B/PARP1/CD8A/CDK5RAP3/SKAP1/FOXO3/MAP2K6/CEP152/BLNK/PPP1CC/IRAK1/TNIP1/CASP9/NFATC1/IL12RB2/CASP1/FGR/ZPR1/PRKAB1/TRAF2/WAS/ANGPT1/PTPN1/ANK2/SMAD1/SRC/CENPJ/SMAD3/SV2A/MAP3K5/MAVS/GRK5/TCL1A
GOMF_PROTEIN_HOMODIMERIZATION_ACTIVITY	-0.3406	-1.5536	0.0006	0.0359	AXIN1/RRM2/MME/TRIM21/ERP29/GGCT/CEACAM5/IMPA1/LRRFIP1/TERF1/WARS1/TRIM5/TFRC/DUSP29/CEBPB/JAM3/NECTIN1/NUDT16/CDA/GBP1/CHEK2/FMR1/GID8/GRHPR/ATP5IF1/LHPP/PRKRA/ENO1/TPR/COMMD1/CACYBP/CASQ2/SETMAR/BAX/F11R/NAGA/GPD1/IKBKG/DPY30/MYH9/GIMAP7/RAB11FIP3/RABEP1/ATF2/TARBP2/PDGFA/CORO1A/ZHX2/ZBTB16/VPS4B/SHMT1/APPL2/BNIP3L/BCL2/MYOM3/TAP1/IRAK1/ZNF174/HMOX1/RBPMS2/CCL5/PDGFB/MYO9B/GBP2/PADI2/PRTFDC1/SMAD3/PKD2/MAP3K5
GOBP_REGULATION_OF_CELL_CYCLE	-0.3456	-1.5677	0.0008	0.0400	EIF4E/LATS1/ANXA1/EIF4G1/STK11/XIAP/RRM2B/EDN1/NPM1/LIF/PTTG1/RRM2/DUSP3/CCND2/TRIM21/PAGR1/EREG/PDGFRB/ESPL1/TERF1/USP28/RGCC/CDK1/NUDT16/SRPK2/TRIAP1/MNAT1/CASP3/CHEK2/CHMP6/DYNLT3/TP53/ASAH2/MAD1L1/CCAR2/MAEA/TPR/CEP85/TP73/SETMAR/CDKN1A/CLSPN/ABL1/BAX/YY1/RAB11FIP3/ENTR1/RCC1/ATF2/SIRT1/FEN1/UHRF2/EGF/CDK5RAP3/ZNF830/GIGYF2/VPS4B/HTRA2/APPL2/IFNW1/CETN2/BCL2/MAP2K6/NBN/CASP2/TP53BP1/MORF4L1/EVI2B/TSC1/PDGFB/NEK7/RUVBL1/SRC/CENPJ/PKD2/GRK5
GOBP_POSITIVE_REGULATION_OF_NUCLEOBASE_CONTAINING_COMPOUND_METABOLIC_PROCESS	-0.3299	-1.5818	0.0001	0.0111	DDX58/EDN1/NPM1/RFC4/LIF/AP3B1/NFIC/ERN1/TNFSF11/AXIN1/TRIM21/PAGR1/CREB3/CREBZF/DAB2/ITGA6/PDGFRB/EDF1/RGCC/TET2/CRX/TRIM5/MGMT/TFRC/CEBPB/FUS/TRIAP1/PITHD1/NFYA/CHEK2/NFE2/TP53/ERCC1/MYDGF/HDGF/CCAR2/PRKCQ/HIF1A/TF/ENO1/TRIM24/TP73/SETMAR/PPP1R12A/IFNL1/ABL1/BAX/GPD1/YY1/IKBKG/PF4/REST/TLR3/F2R/ATF2/TEF/FOSB/SIRT1/RNF168/HNRNPK/GLYR1/PARP1/EGF/CDK5RAP3/ZBTB16/GIGYF2/DXO/SKAP1/PTGES2/SMARCA2/FOXJ3/HDGFL2/WWP2/CCN1/EPCAM/CAPN3/YES1/FOXO3/NAMPT/RET/ARID4B/NBN/LYAR/GFER/ING1/TP53BP1/TNIP1/MORF4L1/NFATC1/ZPR1/FOXO1/TRIM25/WAS/PDGFB/NEK7/RUVBL1/SMAD1/SRC/CENPJ/SMAD3/PKD2/GAL/MAP3K5/CALCOCO1/MAVS/EIF2AK3/NFAT5
GOMF_PROTEIN_DIMERIZATION_ACTIVITY	-0.3396	-1.5838	0.0005	0.0311	ERBB3/NPM1/GPHA2/ERN1/AXIN1/RRM2/MME/TRIM21/ERP29/GGCT/CEACAM5/IMPA1/LRRFIP1/TERF1/WARS1/TRIM5/TFRC/DUSP29/CEBPB/JAM3/NECTIN1/NUDT16/CDA/GBP1/CHEK2/FMR1/TOP2B/TP53/GID8/GRHPR/ATP5IF1/LHPP/PRKRA/HIF1A/ENO1/TPR/COMMD1/CACYBP/IL12B/CASQ2/SETMAR/AIFM1/BAX/F11R/RILPL2/NAGA/GPD1/IKBKG/DPY30/MYH9/GIMAP7/RAB11FIP3/RABEP1/RCC1/ATF2/TARBP2/TCOF1/PDGFA/CORO1A/ZHX2/ZBTB16/VPS4B/CEACAM8/SHMT1/APPL2/BNIP3L/BCL2/MYOM3/TAP1/IRAK1/ZNF174/HMOX1/RBPMS2/CCL5/PDGFB/MYO9B/GBP2/PADI2/PRTFDC1/SMAD3/PKD2/MAP3K5/THAP12
GOBP_CELLULAR_AMIDE_METABOLIC_PROCESS	-0.3473	-1.5857	0.0007	0.0395	CTSH/NCK2/GCLM/CIRBP/ATP6AP2/ST3GAL1/GNPDA1/METAP1/ENPEP/LSM1/CEBPA/DARS1/ASAH1/PAIP2B/GNE/PCSK7/ATXN2/ERBB2/EIF2S2/ITGA2/BTD/GSR/PMVK/EIF4E/EIF4G1/NPM1/CPXM1/MME/RPS10/TMED10/RWDD1/BCHE/DRG2/WARS1/NAAA/DDX1/NAGK/CASP3/FMR1/TP53/PGLYRP2/DMD/ASAH2/ACSL1/BANK1/EIF5/IGF2BP3/PPT1/TPR/CLIC5/NAGA/THOP1/NGRN/DENR/LTO1/CHAC2/PM20D1/TARBP2/TCOF1/SMPDL3B/GIGYF2/PTGES2/ARG2/HTRA2/SHMT1/CCN1/NT5C/YARS1/SPTLC1/MTIF3/FOXO3/EEF1D/LARP1/TNIP1/EIF2AK2/CCL5/MVK/TSC1/GTPBP2/VIM/GSTT2B/PRG3/EIF2AK3/EIF4G3
GOCC_MICROTUBULE_CYTOSKELETON	-0.3484	-1.5930	0.0003	0.0281	MAP1LC3B2/ESPL1/TERF1/RGCC/CDK1/SLC16A1/SNAP29/TBCB/TPT1/CHMP6/DYNLT3/TP53/MAD1L1/CEP112/CEP350/CCAR2/MAEA/CEP164/PRKCQ/TXNDC9/DDHD2/TPR/ITGB6/CLIP2/ACTA2/CEP85/CLIC5/RELB/PPP1R12A/PRKAR1A/RILPL2/CC2D1A/IKBKG/NGRN/MYH9/PIBF1/SDCCAG8/PXN/RAB11FIP3/ENTR1/EVI5/CDK5RAP3/VPS4B/PSMA1/CETN2/MYCBP2/USO1/YES1/CEP152/MZT1/TAP1/SNAPIN/CEP170/HK2/DAAM1/CAMSAP1/PDE4D/VIM/NEK7/UMOD/DBH/RUVBL1/SLMAP/CENPJ/SMTN/PKD2/CRACR2A/PDZD2/IRAG2
GOBP_PROTEIN_LOCALIZATION_TO_ORGANELLE	-0.3719	-1.6166	0.0011	0.0479	DAG1/LATS1/STK11/M6PR/LIF/AP3B1/SRP14/TMED10/LAMTOR5/TERF1/BAG4/FGF9/CDK1/DDX1/TFRC/GBP1/TP53/HDGF/CEP350/DNAJB6/PIKFYVE/ATP5IF1/MICALL2/TPR/RAB6A/CDKN1A/AIFM1/TRIM40/BAX/PIBF1/OTUD7B/RAB11FIP3/RABEP1/ENTR1/ATF2/FIS1/DLG4/PARP1/EGF/CDK5RAP3/ZBTB16/TIMM8A/HTRA2/APPL2/BNIP3L/CAMLG/ING1/RAB10/HK2/ZPR1/ANGPT1/SSB/GBP2/ANK2/SRC/SMAD3/STAM/LACRT/MAVS/EIF2AK3
GOBP_CELLULAR_MACROMOLECULE_BIOSYNTHETIC_PROCESS	-0.3674	-1.6904	0.0001	0.0111	GYS1/PAIP2B/FGF2/GALNT5/CCNE1/ATXN2/CENPF/ERBB2/EIF2S2/ITGA2/PMM2/EIF4E/EIF4G1/RRM2B/NPM1/FUT1/RFC4/NFIC/GALNT3/RRM2/RPS10/RWDD1/EREG/PDGFRB/TERF1/DRG2/WARS1/RGCC/CDK1/TET2/DDX1/SRPK2/NDST1/CDAN1/FMR1/TP53/HS6ST1/BANK1/EIF5/IGF2BP3/CCL19/PRKCQ/HIF1A/TPR/SETMAR/CDKN1A/EXOSC10/DTD1/YY1/NGRN/DENR/LTO1/OGA/TOP1/TARBP2/SIRT1/TCOF1/PDGFA/FEN1/TOP1MT/EGF/DUT/ZNF830/GIGYF2/TMEM106A/SHMT1/YARS1/MTIF3/FOXO3/BCL2/EEF1D/NBN/LARP1/GFER/TNIP1/EIF2AK2/CCL5/ZPR1/TSC1/GTPBP2/VIM/PDGFB/NEK7/RUVBL1/SRC/HBEGF/PRG3/EIF2AK3/EIF4G3

GOCC_MITOCHONDRION	-0.3625	-1.7002	0.0000	0.0047	ACOT13/ALDH5A1/NDUFS6/ACP6/ARAF/BTD/PPIF/IFIT3/GSR/NUDT2/STK11/RRM2B/ECI2/AP3B1/ERN1/FDX1/COX5B/CREBZF/LONP1/IVD/TXNRD1/ECHS1/CDK1/DDX1/TFRC/TRIAP1/CYB5A/LACTB2/MAPK9/TP53/ASAH2/ACSL1/WASF1/LRRC59/CCAR2/ATP5IF1/PDP1/BCAT1/AIFM1/ATP5PO/CIAPIN1/ABL1/BAX/THOP1/C2orf69/NGRN/PLSCR3/PFDN2/RAB11FIP3/ATF2/COX6B1/NRGN/SIRT1/FEN1/HMGCL/FIS1/MSRA/SNAP23/PARP1/TOP1MT/DUT/GIMAP8/TIMM8A/PTGES2/NFU1/OXCT1/ARG2/HTRA2/NDUFB7/BNIP3L/IMMT/RTN4IP1/RNF5/UNG/MTIF3/FOXO3/BCL2/DIABLO/SPARC/ARID4B/PHYKPL/GFER/CASP2/DECR1/PPP1CC/ARSB/CASP9/HK2/SPART/HMOX1/FGR/FOXO1/PTPN1/ANK2/CMC1/SRC/MAVS/OLFM4
GOBP_PROCESS_UTILIZING_AUTOPHAGIC_MECHANISM	-0.4082	-1.7020	0.0007	0.0388	ATP6V1G1/EIF4E/EIF4G1/STK11/ERN1/TRIM21/LGALS8/MAP1LC3B2/VTI1A/TRIM5/SNAP29/CASP3/CHMP6/TP53/TBC1D5/PIKFYVE/ATP5IF1/HIF1A/ABL1/IKBKG/RNF41/SIRT1/FIS1/CD84/VAMP8/VPS4B/HTRA2/BNIP3L/RNF5/SPTLC1/FOXO3/BCL2/LARP1/SNAPIN/ARSB/TAB2/HK2/HMOX1/DAPK2/FOXO1/TSC1/SRC/STAM/LACRT/EIF4G3
GOBP_CHROMOSOME_ORGANIZATION	-0.4229	-1.7609	0.0005	0.0328	LATS1/ANXA1/NPM1/RFC4/PTTG1/AXIN1/CREBZF/ESPL1/TERF1/CDK1/TET2/DDX1/MNAT1/BRD3/CDAN1/CBX2/CHMP6/NFE2/TOP2B/TP53/ERCC1/MAD1L1/PRKCQ/MPHOSPH8/TSPYL1/TPR/JMJD1C/TPRKB/SETMAR/AIFM1/ABL1/EXOSC10/YY1/PIBF1/LTO1/TOP1/SIRT1/RNF168/FEN1/PARP1/TOP1MT/ZNF830/PADI4/VPS4B/SMARCA2/MLLT1/ARID4B/NBN/SATB1/NEK7/FAM172A/PADI2/RUVBL1/SRC
GOBP_REGULATION_OF_CELLULAR_AMIDE_METABOLIC_PROCESS	-0.4472	-1.7626	0.0009	0.0445	METAP1/LSM1/PAIP2B/ATXN2/ERBB2/ITGA2/EIF4E/EIF4G1/NPM1/TMED10/DDX1/CASP3/FMR1/TP53/BANK1/EIF5/IGF2BP3/TPR/NGRN/TARBP2/TCOF1/GIGYF2/SHMT1/CCN1/FOXO3/LARP1/EIF2AK2/CCL5/TSC1/VIM/PRG3/EIF2AK3/EIF4G3
GOCC_MICROTUBULE_ORGANIZING_CENTER	-0.4075	-1.7630	0.0002	0.0179	TP53/MAD1L1/CEP112/CEP350/CEP164/PRKCQ/TXNDC9/DDHD2/ITGB6/CEP85/CLIC5/RELB/PPP1R12A/PRKAR1A/RILPL2/CC2D1A/PIBF1/SDCCAG8/RAB11FIP3/ENTR1/EVI5/CDK5RAP3/VPS4B/PSMA1/CETN2/USO1/YES1/CEP152/MZT1/TAP1/CEP170/HK2/DAAM1/PDE4D/VIM/NEK7/DBH/RUVBL1/SLMAP/CENPJ/SMTN/PKD2/CRACR2A/PDZD2/IRAG2
GOBP_ORGANIC_CYCLIC_COMPOUND_CATABOLIC_PROCESS	-0.4275	-1.7716	0.0005	0.0311	TET2/TRDMT1/PDE5A/NUDT16/FUS/CDA/FMR1/DFFA/HMOX2/SETMAR/AIFM1/EXOSC10/BAX/SKIV2L/ERI1/NUDT15/SCARF1/FEN1/DUT/ZHX2/GIGYF2/DXO/NT5C/UNG/LARP1/HMOX1/ZPR1/PDE4D/TRAF2/VIM/SSB/PRTFDC1/DBH/RNASE3
GOCC_ENVELOPE	-0.4029	-1.7784	0.0000	0.0047	TRIAP1/CYB5A/DNAJB14/CHMP6/ERBIN/MAD1L1/ACSL1/WASF1/LRRC59/ENO1/TPR/CACYBP/LBR/AIFM1/ATP5PO/CIAPIN1/ABL1/BAX/THOP1/NGRN/PLSCR3/RCC1/ATF2/COX6B1/NRGN/SIRT1/FIS1/PARP1/TIMM8A/GHRHR/HTRA2/NDUFB7/BNIP3L/IMMT/RTN4IP1/RNF5/CETN2/FOXO3/BCL2/DIABLO/GFER/PPP1CC/HK2/SPART/HMOX1/FGR/PTPN1/SMAD1/SRC/SMAD3/DTX2/MAVS/GRK5/IRAG2
GOBP_DNA_METABOLIC_PROCESS	-0.4140	-1.7874	0.0001	0.0109	CD40LG/RRM2B/NPM1/RFC4/PTTG1/PAGR1/LONP1/PDGFRB/TERF1/USP28/RGCC/CDK1/TET2/DDX1/MGMT/TFRC/FUS/SRPK2/MNAT1/CHEK2/TOP2B/TP53/ERCC1/DFFA/CEP164/PRKCQ/MPHOSPH8/COMMD1/TPRKB/TP73/SETMAR/CDKN1A/AIFM1/CLSPN/SWAP70/ABL1/EXOSC10/BAX/YY1/NUDT15/LTO1/TOP1/SIRT1/RNF168/PDGFA/FEN1/UHRF2/PARP1/TOP1MT/ZNF830/HDGFL2/CETN2/UNG/PMS1/NBN/GFER/TP53BP1/MORF4L1/WAS/PDGFB/NEK7/RUVBL1/SRC
GOCC_MITOCHONDRIAL_ENVELOPE	-0.4365	-1.7881	0.0003	0.0271	AIFM1/ATP5PO/CIAPIN1/BAX/THOP1/NGRN/PLSCR3/ATF2/COX6B1/NRGN/FIS1/TIMM8A/HTRA2/NDUFB7/BNIP3L/IMMT/RTN4IP1/RNF5/FOXO3/BCL2/DIABLO/GFER/PPP1CC/HK2/SPART/HMOX1/FGR/PTPN1/SRC/MAVS
GOBP_CELLULAR_NITROGEN_COMPOUND_CATABOLIC_PROCESS	-0.4414	-1.8025	0.0006	0.0342	TET2/TRDMT1/PDE5A/NUDT16/FUS/CDA/FMR1/DFFA/HMOX2/SETMAR/AIFM1/EXOSC10/BAX/SKIV2L/ERI1/NUDT15/PM20D1/FEN1/DUT/ZHX2/GIGYF2/DXO/NT5C/UNG/LARP1/HMOX1/ZPR1/PDE4D/TRAF2/VIM/SSB/PRTFDC1/RNASE3
GOBP_POSTTRANSCRIPTIONAL_REGULATION_OF_GENE_EXPRESSION	-0.4463	-1.8110	0.0004	0.0309	DDX1/TRDMT1/FUS/FMR1/TP53/BANK1/EIF5/IGF2BP3/PRKRA/TPR/NGRN/TARBP2/TCOF1/GIGYF2/DXO/SHMT1/FOXO3/LARP1/EIF2AK2/CCL5/TSC1/TRAF2/VIM/SMAD1/SMAD3/PRG3/EIF2AK3/EIF4G3
GOBP_RESPONSE_TO_ENDOPLASMIC_RETICULUM_STRESS	-0.4784	-1.8189	0.0005	0.0333	EIF4G1/ERN1/PDIA4/ERP29/CREB3/AMFR/CEBPB/DNAJB14/TP53/AIFM1/ABL1/BAX/SIRT1/CDK5RAP3/MANF/RNF5/BCL2/FCGR2B/EIF2AK2/TRIM25/TRAF2/PTPN1/FGF21/MAP3K5/EIF2AK3
GOBP_PEPTIDE_BIOSYNTHETIC_PROCESS	-0.4448	-1.8322	0.0002	0.0208	METAP1/LSM1/DARS1/PAIP2B/ATXN2/ERBB2/EIF2S2/ITGA2/EIF4E/EIF4G1/NPM1/RPS10/RWDD1/DRG2/WARS1/DDX1/FMR1/DMD/BANK1/EIF5/IGF2BP3/TPR/NGRN/DENR/LTO1/CHAC2/TARBP2/TCOF1/GIGYF2/SHMT1/YARS1/MTIF3/FOXO3/EEF1D/LARP1/TNIP1/EIF2AK2/CCL5/TSC1/GTPBP2/VIM/PRG3/EIF2AK3/EIF4G3
GOBP_MACROAUTOPHAGY	-0.4828	-1.8355	0.0004	0.0309	ERN1/LGALS8/MAP1LC3B2/VTI1A/SNAP29/CASP3/CHMP6/TP53/TBC1D5/PIKFYVE/HIF1A/IKBKG/RNF41/SIRT1/VAMP8/VPS4B/HTRA2/BNIP3L/RNF5/SPTLC1/LARP1/SNAPIN/HMOX1/TSC1/SRC/STAM/LACRT
GOMF_TRANSCRIPTION_COREGULATOR_ACTIVITY	-0.4892	-1.8506	0.0004	0.0309	NMI/NPM1/TRIM21/EDF1/DDX1/TRIM5/FUS/SAP18/HDGF/CCAR2/ENO1/TRIM24/JMJD1C/ABL1/PXN/RALY/SIRT1/PSIP1/MED18/SMARCA2/HDGFL2/TP53BP1/TRIM25/RUVBL1/CENPJ/CALCOCO1
GOCC_NUCLEAR_BODY	-0.4480	-1.8567	0.0001	0.0141	INPPL1/TERF1/USP28/DDX1/SRPK2/SAP18/CHEK2/NFE2/FMR1/TP53/ERBIN/LHPP/HIF1A/CDKN1A/ABL1/SAFB2/NGRN/TARBP2/SART1/SIRT1/RBM19/TREML1/SMNDC1/PARP1/ZBTB16/ZNF830/KAZN/SNRPB2/RBM25/BNIP3L/YARS1/NAMPT/NBN/NMNAT1/PPP1CC/SATB1/TP53BP1/MORF4L1/NFATC1/ZPR1/TRIM25/MINDY1/GRK5
GOBP_AMIDE_BIOSYNTHETIC_PROCESS	-0.4447	-1.8623	0.0001	0.0107	NCK2/GCLM/CIRBP/ST3GAL1/METAP1/LSM1/CEBPA/DARS1/ASAH1/PAIP2B/ATXN2/ERBB2/EIF2S2/ITGA2/EIF4E/EIF4G1/NPM1/RPS10/RWDD1/DRG2/WARS1/DDX1/FMR1/DMD/ASAH2/ACSL1/BANK1/EIF5/IGF2BP3/PPT1/TPR/NGRN/DENR/LTO1/CHAC2/PM20D1/TARBP2/TCOF1/GIGYF2/ARG2/SHMT1/CCN1/YARS1/SPTLC1/MTIF3/FOXO3/EEF1D/LARP1/TNIP1/EIF2AK2/CCL5/TSC1/GTPBP2/VIM/PRG3/EIF2AK3/EIF4G3
GOMF_CHROMATIN_BINDING	-0.4930	-1.8706	0.0004	0.0309	CDK1/DDX1/CEBPB/MPO/FUS/BRD3/CBX2/FMR1/TOP2B/TP53/ERCC1/TRIM24/MPHOSPH8/TSPYL1/TPR/JMJD1C/YY1/REST/RCC1/ATF2/TOP1/SIRT1/RNF168/GLYR1/SMARCA2/FOXO3/MORF4L1/FOXO1/SMAD3/CALCOCO1
GOBP_DNA_REPAIR	-0.4934	-1.8721	0.0004	0.0309	RRM2B/NPM1/RFC4/PTTG1/PAGR1/USP28/CDK1/DDX1/MGMT/FUS/MNAT1/CHEK2/TP53/ERCC1/CEP164/COMMD1/TP73/SETMAR/CLSPN/ABL1/YY1/SIRT1/RNF168/FEN1/PARP1/HDGFL2/CETN2/UNG/PMS1/NBN/TP53BP1/MORF4L1/WAS/RUVBL1
GOMF_DNA_BINDING_TRANSCRIPTION_FACTOR_BINDING	-0.4752	-1.8904	0.0001	0.0141	CENPF/PRAME/EIF4E/LATS1/NPM1/FLT3/PAGR1/NAAA/CRX/CEBPB/TPT1/TP53/CRKL/FBP1/HIF1A/TRIM24/JMJD1C/TP73/YY1/REST/ATF2/SIRT1/UHRF2/PARP1/CDK5RAP3/WWP2/BCL2/NBN/LYAR/TP53BP1/NFATC1/PADI2/SRC/SMAD3
GOBP_CELLULAR_RESPONSE_TO_DNA_DAMAGE_STIMULUS	-0.4519	-1.9040	0.0000	0.0046	STK11/XIAP/RRM2B/NPM1/RFC4/MIF/PTTG1/VAV3/PAGR1/USP28/CDK1/DDX1/MGMT/FUS/TPT1/TRIAP1/MNAT1/CASP3/CHEK2/FMR1/TP53/ERCC1/YJU2/CCAR2/CEP164/COMMD1/TP73/SETMAR/CDKN1A/MNDA/CLSPN/ABL1/BAX/YY1/IKBKG/ATF2/SIRT1/RNF168/FEN1/HNRNPK/PARP1/CDK5RAP3/ZNF830/GIGYF2/HTRA2/HDGFL2/CETN2/UNG/FOXO3/BCL2/MAP2K6/PMS1/NBN/CASP2/TP53BP1/CASP9/MORF4L1/HMOX1/FOXO1/WAS/RUVBL1
GOMF_PROTEIN_SERINE_KINASE_ACTIVITY	-0.5519	-1.9211	0.0005	0.0311	ARAF/LATS1/STK11/ERN1/CDK1/SRPK2/CHEK2/MAPK9/PIKFYVE/PRKCQ/AKT3/STK24/MAP2K6/IRAK1/EIF2AK2/DAPK2/CDC42BPB/NEK7/MAP3K5/EIF2AK3

GOBP_NCRNA_PROCESSING	-0.6374	-1.9240	0.0011	0.0493	DDX1/TRDMT1/NUDT16/PRKRA/TPRKB/EXOSC10/ERI1/TARBP2/SART1/WDR46/LYAR/SSB/SMAD1/SMAD3
GOBP_PROTEIN_MODIFICATION_BY_SMALL_PROTEIN_CONJUGATION	-0.4823	-1.9333	0.0001	0.0126	XIAP/NMI/AXIN1/BRAP/TRIM21/AMFR/TRIM5/MAPK9/BLMH/MAEA/HIF1A/TRIM24/COMMD1/TRIM40/ABL1/TRIM26/THOP1/NUB1/RNF41/SIRT1/RNF168/UHRF2/CDK5RAP3/ZBTB16/WWP2/RNF5/MYCBP2/CAPN3/BCL2/CAMLG/DDA1/TRIM25/TRAF2/ANGPT1/DTX2
GOMF_SEQUENCE_SPECIFIC_DNA_BINDING	-0.4671	-1.9337	0.0000	0.0068	CREB3/CREBZF/LONP1/LRRFIP1/TERF1/CRX/CEBPB/NFYA/NFE2/TP53/HDGF/HIF1A/ENO1/TRIM24/TP73/RELB/ABL1/SAFB2/CC2D1A/YY1/REST/ATF2/TEF/FOSB/TOP1/SIRT1/ZBTB16/SMARCA2/FOXJ3/NACC1/FOXO3/BCL2/ARID4B/SATB1/TP53BP1/ZNF174/NFATC1/FOXO1/SMAD1/SMAD3/CALCOCO1/NFAT5
GOBP_NEGATIVE_REGULATION_OF_CELL_CYCLE_PHASE_TRANSITION	-0.5760	-1.9351	0.0006	0.0357	USP28/RGCC/CDK1/TRIAP1/CHEK2/TP53/MAD1L1/CCAR2/TPR/SETMAR/CDKN1A/CLSPN/ATF2/CDK5RAP3/ZNF830/GIGYF2/BCL2/NBN/CASP2/TP53BP1/PKD2
GOCC_TRANSCRIPTION_REGULATOR_COMPLEX	-0.5351	-1.9390	0.0003	0.0277	CREB3/CREBZF/CRX/CEBPB/MNAT1/NFYA/NFE2/TP53/HDGF/HIF1A/RELB/YY1/REST/ATF2/MED18/PARP1/ZBTB16/FOXO3/NFATC1/SMAD1/SMAD3/NFAT5
GOMF_RNA_BINDING	-0.4225	-1.9439	0.0000	0.0001	PDIA4/RPS10/TRIM21/DNM1/SRP14/HNRNPUL1/LONP1/EDF1/LRRFIP1/DRG2/BAG4/DDX1/TFRC/PPIB/TRDMT1/NUDT16/FUS/TPT1/SRPK2/METAP2/LACTB2/SAP18/FMR1/TP53/AHNAK/HDGF/CRKL/LRRC59/EIF5/IGF2BP3/CCAR2/PRKRA/ENO1/TPR/LBR/EXOSC10/SAFB2/DTD1/YY1/GPKOW/SKIV2L/ENDOU/DNAJC21/NGRN/ERI1/MYH9/DENR/TLR3/RALY/TOP1/TARBP2/SART1/TCOF1/PSIP1/HNRNPK/SUGP1/CORO1A/RBM19/WDR46/SMNDC1/PARP1/DUT/C7orf50/GIGYF2/DXO/SNRPB2/PSMA1/SHMT1/RBM25/IMMT/MANF/YARS1/USO1/EPPK1/MTIF3/EEF1D/LARP1/RBM17/LYAR/PPP1CC/EIF2AK2/RBPMS2/GTPBP2/TRIM25/VIM/PTPN1/SSB/FAM172A/SMAD1/EIF4G3
GOBP_REGULATION_OF_CELLULAR_MACROMOLECULE_BIOSYNTHETIC_PROCESS	-0.4642	-1.9556	0.0000	0.0031	EREG/PDGFRB/TERF1/RGCC/CDK1/DDX1/CDAN1/FMR1/TP53/BANK1/EIF5/IGF2BP3/CCL19/PRKCQ/TPR/CDKN1A/EXOSC10/YY1/NGRN/TARBP2/TCOF1/PDGFA/EGF/ZNF830/GIGYF2/SHMT1/FOXO3/BCL2/NBN/LARP1/GFER/EIF2AK2/CCL5/TSC1/VIM/PDGFB/NEK7/RUVBL1/SRC/HBEGF/PRG3/EIF2AK3/EIF4G3
GOBP_DOUBLE_STRAND_BREAK_REPAIR	-0.6116	-1.9579	0.0006	0.0362	DDX1/MGMT/FUS/CHEK2/TP53/ERCC1/SETMAR/YY1/RNF168/FEN1/PARP1/HDGFL2/NBN/TP53BP1/MORF4L1/WAS/RUVBL1
GOBP_RECOMBINATIONAL_REPAIR	-0.7229	-1.9581	0.0012	0.0500	YY1/FEN1/PARP1/HDGFL2/NBN/TP53BP1/MORF4L1/WAS/RUVBL1
GOCC_NUCLEOLUS	-0.4703	-1.9585	0.0000	0.0042	NPM1/NFIC/GOLGA3/RPS10/CCND2/DAB2/TXNRD1/EDF1/TERF1/RGCC/NUDT16/SRPK2/FMR1/TOP2B/TP53/MPHOSPH8/TSPYL1/NFKBIE/CEP85/SETMAR/CDKN1A/PPP1R12A/MNDA/CIAPIN1/ABL1/EXOSC10/CC2D1A/NUB1/DNAJC21/ERI1/TOP1/SIRT1/TCOF1/FEN1/RBM19/WDR46/STK24/PARP1/CDK5RAP3/MLLT1/SNRPB2/USO1/CAPN3/EEF1D/NBN/LYAR/PPP1CC/HMOX1/ZPR1
GOCC_NUCLEAR_PROTEIN_CONTAINING_COMPLEX	-0.4721	-1.9643	0.0000	0.0030	PAGR1/CREB3/CREBZF/TTF2/TERF1/CRX/CEBPB/MNAT1/NFYA/SAP18/CBX2/CHMP6/NFE2/ERCC1/MAD1L1/YJU2/HIF1A/TPR/JMJD1C/CDKN1A/EXOSC10/YY1/GPKOW/DPY30/ERI1/RALY/ATF2/SART1/SIRT1/HNRNPK/SUGP1/ELOA/SMNDC1/MED18/ZNF830/SMARCA2/MLLT1/SNRPB2/CETN2/FOXO3/NBN/RBM17/MORF4L1/RUVBL1/SMAD1/SMAD3
GOBP_MITOTIC_CELL_CYCLE_CHECKPOINT_SIGNALING	-0.6592	-1.9756	0.0008	0.0395	CDK1/TRIAP1/CHEK2/TP53/MAD1L1/TPR/SETMAR/CDKN1A/CLSPN/ATF2/CDK5RAP3/ZNF830/GIGYF2/NBN/CASP2
GOBP_POSITIVE_REGULATION_OF_DNA_REPAIR	-0.7011	-1.9789	0.0008	0.0395	MGMT/FUS/SETMAR/SIRT1/RNF168/PARP1/HDGFL2/MORF4L1/WAS/RUVBL1
GOBP_NEGATIVE_REGULATION_OF_CELLULAR_MACROMOLECULE_BIOSYNTHETIC_PROCESS	-0.5794	-1.9967	0.0001	0.0141	CDAN1/FMR1/TP53/BANK1/IGF2BP3/TPR/CDKN1A/EXOSC10/ZNF830/GIGYF2/SHMT1/LARP1/EIF2AK2/TSC1/SRC/HBEGF/PRG3/EIF2AK3
GOBP_RIBONUCLEOPROTEIN_COMPLEX_BIOGENESIS	-0.6168	-1.9981	0.0004	0.0311	DDX1/NUDT16/SRPK2/YJU2/EIF5/PRKRA/EXOSC10/NGRN/ERI1/DENR/LTO1/TARBP2/SART1/PSIP1/WDR46/LYAR/TSC1/RUVBL1
GOBP_PROTEIN_MODIFICATION_BY_SMALL_PROTEIN_CONJUGATION_OR_REMOVAL	-0.4839	-2.0030	0.0000	0.0034	PRAME/OTUD6B/XIAP/NMI/AXIN1/BRAP/TRIM21/AMFR/USP28/CDK1/TRIM5/USP8/MAPK9/BLMH/MAEA/HIF1A/TRIM24/COMMD1/TRIM40/ABL1/TRIM26/THOP1/NUB1/OTUD7B/RNF41/SIRT1/RNF168/UHRF2/CDK5RAP3/ZBTB16/WWP2/RNF5/MYCBP2/CAPN3/BCL2/CAMLG/DDA1/TNIP1/TRIM25/TRAF2/ANGPT1/MINDY1/DTX2
GOBP_POSITIVE_REGULATION_OF_RESPONSE_TO_DNA_DAMAGE_STIMULUS	-0.6905	-2.0131	0.0006	0.0342	MGMT/FUS/FMR1/CCAR2/SETMAR/SIRT1/RNF168/PARP1/HDGFL2/MORF4L1/WAS/RUVBL1
GOBP_RNA_PROCESSING	-0.4962	-2.0137	0.0000	0.0046	HNRNPUL1/TTF2/DDX1/TRDMT1/NUDT16/FUS/SRPK2/SAP18/FMR1/TP53/AHNAK/YJU2/CCAR2/PRKRA/TPRKB/EXOSC10/SAFB2/GPKOW/ERI1/REST/RALY/TARBP2/SART1/PSIP1/HNRNPK/SUGP1/RBM19/WDR46/SMNDC1/ZNF830/SNRPB2/RBM25/RBM17/LYAR/ZPR1/SSB/FAM172A/SMAD1/SMAD3
GOBP_ORGANELLE_DISASSEMBLY	-0.6749	-2.0226	0.0004	0.0309	MAP1LC3B2/CDK1/TP53/ATP5IF1/HIF1A/DENR/RNF41/FIS1/HTRA2/BNIP3L/MTIF3/HK2
GOMF_DNA_BINDING_TRANSCRIPTION_FACTOR_ACTIVITY	-0.5300	-2.0227	0.0001	0.0107	CREB3/CREBZF/LRRFIP1/CRX/CEBPB/NFYA/NFE2/TP53/HDGF/HIF1A/ENO1/TP73/RELB/CC2D1A/YY1/REST/ATF2/TEF/FOSB/ZHX2/ZBTB16/FOXJ3/NACC1/FOXO3/SATB1/ZNF174/NFATC1/FOXO1/SMAD1/SMAD3/NFAT5
GOMF_TRANSCRIPTION_FACTOR_BINDING	-0.4894	-2.0279	0.0000	0.0022	FOS/CEBPA/BAIAP2/CENPF/PRAME/EIF4E/LATS1/NPM1/FLT3/PAGR1/EDF1/NAAA/CRX/CEBPB/TPT1/SAP18/TP53/ERCC1/HDGF/CRKL/FBP1/HIF1A/ENO1/TRIM24/JMJD1C/TP73/YY1/REST/ATF2/FOSB/SIRT1/UHRF2/PARP1/CDK5RAP3/ZBTB16/WWP2/FOXO3/BCL2/NBN/LYAR/TP53BP1/NFATC1/PADI2/RUVBL1/SRC/SMAD3/NFAT5
GOCC_CHROMATIN	-0.4923	-2.0418	0.0000	0.0012	CCND2/CREB3/CREBZF/CRX/CEBPB/SRPK2/NFYA/BRD3/CBX2/NFE2/TOP2B/TP53/DFFA/CCAR2/HIF1A/TRIM24/MPHOSPH8/TSPYL1/JMJD1C/TP73/RELB/EXOSC10/YY1/RCC1/ATF2/TEF/FOSB/SIRT1/PSIP1/HNRNPK/GLYR1/UHRF2/ZHX2/SMARCA2/HTRA2/FOXJ3/FOXO3/SATB1/ZNF174/MORF4L1/NFATC1/FOXO1/PADI2/RUVBL1/SMAD1/SMAD3/CALCOCO1/NFAT5
GOBP_MRNA_METABOLIC_PROCESS	-0.5058	-2.0433	0.0000	0.0035	DDX39A/NPM1/AP3B1/ERN1/EREG/TTF2/DDX1/NUDT16/FUS/SRPK2/SAP18/FMR1/TP53/YJU2/CCAR2/EXOSC10/SAFB2/GPKOW/SKIV2L/ERI1/REST/RALY/ATF2/SART1/PSIP1/HNRNPK/SUGP1/RBM19/SMNDC1/ZHX2/ZNF830/GIGYF2/DXO/SNRPB2/RBM25/LARP1/RBM17/ZPR1/TRAF2/VIM/SSB/FAM172A

GOBP_SIGNAL_TRANSDUCTION_IN_RESPONSE_TO_DNA_DAMAGE	-0.6258	-2.0554	0.0002	0.0168	USP28/CDK1/TRIAP1/CHEK2/TP53/YJU2/CCAR2/CDKN1A/CLSPN/ABL1/ATF2/SIRT1/CDK5RAP3/ZNF830/GIGYF2/FOXO3/NBN/CASP2/TP53BP1/CASP9
GOBP_MITOTIC_DNA_INTEGRITY_CHECKPOINT_SIGNALING	-0.7184	-2.0628	0.0003	0.0271	CDK1/TRIAP1/CHEK2/TP53/SETMAR/CDKN1A/CLSPN/ATF2/CDK5RAP3/ZNF830/GIGYF2/NBN/CASP2
GOMF_CIS_REGULATORY_REGION_SEQUENCE_SPECIFIC_DNA_BINDING	-0.5381	-2.0708	0.0000	0.0040	CREB3/LRRFIP1/CRX/CEBPB/NFYA/NFE2/TP53/HDGF/HIF1A/TRIM24/TP73/RELB/CC2D1A/YY1/REST/ATF2/TEF/FOSB/TOP1/SIRT1/ZBTB16/FOXJ3/NACC1/FOXO3/SATB1/ZNF174/NFATC1/FOXO1/SMAD1/SMAD3/CALCOCO1/NFAT5
GOBP_CELL_CYCLE_CHECKPOINT_SIGNALING	-0.6764	-2.1012	0.0001	0.0111	USP28/CDK1/TRIAP1/CHEK2/TP53/MAD1L1/CCAR2/TPR/SETMAR/CDKN1A/CLSPN/ATF2/CDK5RAP3/ZNF830/GIGYF2/NBN/CASP2/TP53BP1
GOBP_DNA_INTEGRITY_CHECKPOINT_SIGNALING	-0.7286	-2.1417	0.0001	0.0090	USP28/CDK1/TRIAP1/CHEK2/TP53/CCAR2/SETMAR/CDKN1A/CLSPN/ATF2/CDK5RAP3/ZNF830/GIGYF2/NBN/CASP2/TP53BP1
GOMF_TRANSCRIPTION_REGULATOR_ACTIVITY	-0.5137	-2.1986	0.0000	0.0000	NMI/NPM1/NFIC/TRIM21/CREB3/CREBZF/EDF1/LRRFIP1/DDX1/CRX/TRIM5/CEBPB/FUS/NFYA/SAP18/NFE2/TP53/HDGF/CCAR2/HIF1A/ENO1/TRIM24/JMJD1C/TP73/RELB/ABL1/CC2D1A/YY1/PXN/REST/RALY/ATF2/TEF/FOSB/SIRT1/PSIP1/MED18/ZHX2/ZBTB16/SMARCA2/FOXJ3/NACC1/HDGFL2/FOXO3/SATB1/TP53BP1/ZNF174/NFATC1/FOXO1/TRIM25/RUVBL1/SMAD1/CENPJ/SMAD3/CALCOCO1/NFAT5
GOCC_CHROMOSOME	-0.5057	-2.2201	0.0000	0.0000	CDK1/CRX/CEBPB/SRPK2/NFYA/BRD3/CBX2/CHEK2/CHMP6/NFE2/DYNLT3/FMR1/TOP2B/TP53/ERCC1/MAD1L1/DFFA/CCAR2/HIF1A/TRIM24/MPHOSPH8/TSPYL1/TPR/JMJD1C/TP73/SETMAR/RELB/PPP1R12A/EXOSC10/YY1/DPY30/RCC1/ATF2/TEF/FOSB/TOP1/SIRT1/RNF168/PSIP1/FEN1/HNRNPK/GLYR1/UHRF2/RBM19/ELOA/PARP1/TOP1MT/ZHX2/ZNF830/SMARCA2/HTRA2/FOXJ3/FOXO3/NBN/PPP1CC/SATB1/TP53BP1/ZNF174/MORF4L1/RASSF2/NFATC1/FOXO1/WAS/SSB/PADI2/RUVBL1/SMAD1/SMAD3/CALCOCO1/NFAT5/IRAG2

Table S 3*: Results from STRING protein-protein-interaction analysis in DS vs. HC.*

Term ID	***Term***	***Strength***	***FDR P value***	***matching proteins in the network***
Cluster 1				
GO:0002376	*Immune system process*	*0.24*	*0.0023*	*CD74,TNFRSF17,CD22,LAG3,TGFBR3,HMOX1,PI3,CFP,BST2,PDGFRA,TNFRSF13B,FLT4,LY9,IL11,LAMP3,CD27,IFNAR1,THY1,CXADR,CSF1R,IL10RB,AXL,LY6D,CXCL10,HAVCR2,CLU,CD276,CD300LG,CSF1,IL20RB,CD300C,SELE,PDCD1,ICOSLG,SIGLEC10,RET,EPHA2,TGFBR2,MFAP5,CD300A,CXCL9,DLL1,ULBP2,IFNGR1,FCRL1,PTGDS,WFDC2,WFDC12,TNFRSF1B,HLA-E,TNFRSF4,IL4R,UMOD,PDCD1LG2,JAM2,TF,IL1R1,FGFR2,IL10,IL18BP,SLAMF1,TNFRSF11A,CFD,SPON2,CCL15,B2M,FAS,IL19*
GO:0006952	*Defense response*	*0.29*	*0.0023*	*CD74,TNFRSF1A,LAG3,HMOX1,PI3,IL17C,CFP,BST2,LY9,GAL,IGFBP4,IFNAR1,CRH,CXADR,CSF1R,IL10RB,INHBB,FOLR2,AXL,CXCL10,HAVCR2,CLU,CSF1,IL20RB,CD300C,SELE,ICOSLG,SIGLEC10,EPHA2,CXCL9,ULBP2,IFNGR1,WFDC2,WFDC12,HSPG2,TNFRSF1B,HLA-E,TNFRSF4,SDC1,SPP1,IL4R,UMOD,CST3,TF,IL1R1,PENK,IL10,SLAMF1,IL22,CFD,SPON2,CCL15,B2M*
GO:0007155	*Cell adhesion*	*0.33*	*0.0023*	*CD22,ICAM3,OLFM4,TNN,SLURP1,OMG,PDGFRA,CCN3,CDH1,LY9,NID1,VCAN,NRP1,CDH2,EPHA4,THY1,CXADR,CDH15,COL6A3,IGFBP7,FOLR2,AXL,LY6D,COL3A1,RGMB,TNFRSF12A,SELE,SIGLEC10,RET,EPHB4,EPHA2,TGFBR2,COL18A1,CD300A,THBS2,CCN2,CCN5,ITGBL1,FOLR1,SPP1,UMOD,JAM2,PDZD2,ICAM2,SLAMF1,SPON2*
GO:0007166	*Cell surface receptor signaling pathway*	*0.25*	*0.0023*	*CD74,MYOC,TNFRSF17,TNFRSF1A,LAG3,TGFBR3,DKK4,IL17C,SLURP1,PDGFRA,TNFRSF13B,FLT4,NRP1,CD27,ERBB3,IFNAR1,GFRA3,EPHA4,THY1,CSF1R,IL10RB,TFF2,FGFR4,INHBB,AXL,PRL,COL3A1,CXCL10,RGMB,CD276,CSF1,IL20RB,PLAUR,ICOSLG,GFRAL,GFRA1,RET,EPHB4,EPHA2,TGFBR2,CXCL9,DLL1,IFNGR1,CCN2,FCRL1,ROR1,BAMBI,ITGBL1,TNFRSF1B,TNFRSF4,SDC1,TNFRSF19,ACVRL1,EFEMP1,IL4R,UMOD,PDCD1LG2,TF,IL1R1,FGFR2,DKK3,PGF,TNFRSF11A,CFD,CCL15,FAS,CD59*
GO:0042127	*Regulation of cell population proliferation*	*0.29*	*0.0023*	*CD74,CD22,TGFBR3,HMOX1,TNN,SLURP1,BST2,PDGFRA,CCN3,TNFRSF13B,FLT4,TNFRSF8,IL11,NRP1,GAL,ERBB3,CDH2,EPHA4,CXADR,CSF1R,TFF1,FGFR4,IGFBP7,FOLR2,IGFBP6,PRL,MZB1,CXCL10,HAVCR2,CLU,CD276,CSF1,IL20RB,ICOSLG,APOD,TGFBR2,COL18A1,CD300A,CXCL9,DLL1,CCN2,PTGDS,BAMBI,TNFRSF1B,HLA-E,TNFRSF4,ACVRL1,IL4R,UMOD,PDCD1LG2,FGFR2,IL10,SERPINE2,SLAMF1,PGF,TCL1A,TNFRSF11A,TNFRSF9,B2M*
GO:0048519	*Negative regulation of biological process*	*0.17*	*0.0023*	*CD74,MYOC,CD22,TNFRSF1A,LAG3,TGFBR3,HMOX1,DKK4,TFPI2,PROC,TNN,PI3,SLURP1,BST2,PDGFRA,CCN3,TNFRSF13B,CDH1,FLT4,TIMP2,TNFRSF8,IL11,NRP1,LAMP3,GAL,CD27,ERBB3,CDH2,IGFBP4,CRH,EPHA4,THY1,CXADR,CSF1R,NBL1,TFF2,TFF1,CSTB,INHBB,COL6A3,IGFBP7,AXL,IGFBP6,PRL,COL3A1,CXCL10,HAVCR2,CLU,CSF1,IL20RB,PDCD1,PLAUR,ICOSLG,DLK1,SERPINA12,GFRAL,APOD,SIGLEC10,DNM3,EPHA2,TGFBR2,COL18A1,CD300A,DLL1,THBS2,IFNGR1,CCN2,PTGDS,WFDC2,WFDC12,CCN5,HSPG2,BAMBI,TNFRSF1B,HLA-E,TNFRSF4,ACVRL1,EFEMP1,SPP1,IL4R,UMOD,PDCD1LG2,CST3,JAM2,MSR1,FGFR2,IL10,SERPINE2,DKK3,SLAMF1,TCL1A,TNFRSF9,B2M,FAS,CD59,IL19*
GO:0051241	*Negative regulation of multicellular organismal process*	*0.35*	*0.0023*	*CD74,TNFRSF1A,LAG3,HMOX1,DKK4,PROC,BST2,PDGFRA,CCN3,NRP1,CRH,EPHA4,THY1,CXADR,TFF2,INHBB,AXL,PRL,CXCL10,HAVCR2,IL20RB,PDCD1,APOD,EPHA2,TGFBR2,DLL1,THBS2,IFNGR1,PTGDS,HSPG2,TNFRSF1B,ACVRL1,EFEMP1,SPP1,IL4R,PDCD1LG2,CST3,JAM2,IL10,SERPINE2,SLAMF1,B2M,IL19*
GO:0010647	*Positive regulation of cell communication*	*0.27*	*0.0053*	*CD74,MYOC,TNFRSF1A,TGFBR3,HMOX1,BST2,PDGFRA,CCN3,FLT4,NID1,IL11,NRP1,GAL,CD27,ERBB3,CDH2,IGFBP4,CRH,EPHA4,CSF1R,IL10RB,FGFR4,INHBB,AXL,IGFBP6,PRL,COL3A1,HAVCR2,CLU,TNFRSF12A,CSF1,PLAUR,SERPINA12,GFRAL,RET,TGFBR2,DLL1,CCN2,ROR1,BAMBI,GPC5,TNFRSF19,ACVRL1,SPP1,IL1R1,FGFR2,IL10,SERPINE2,SLAMF1,TNFRSF11A,CCL15,FAS,IL19*
GO:0023056	*Positive regulation of signaling*	*0.27*	*0.0055*	*CD74,MYOC,TNFRSF1A,TGFBR3,HMOX1,BST2,PDGFRA,CCN3,FLT4,NID1,IL11,NRP1,GAL,CD27,ERBB3,CDH2,IGFBP4,CRH,EPHA4,CSF1R,IL10RB,FGFR4,INHBB,AXL,IGFBP6,PRL,COL3A1,HAVCR2,CLU,TNFRSF12A,CSF1,PLAUR,SERPINA12,GFRAL,RET,TGFBR2,DLL1,CCN2,ROR1,BAMBI,GPC5,TNFRSF19,ACVRL1,SPP1,IL1R1,FGFR2,IL10,SERPINE2,SLAMF1,TNFRSF11A,CCL15,FAS,IL19*
GO:0006954	*Inflammatory response*	*0.38*	*0.0083*	*TNFRSF1A,HMOX1,IL17C,GAL,IGFBP4,CRH,CSF1R,IL10RB,FOLR2,AXL,CXCL10,HAVCR2,CLU,CSF1,IL20RB,SELE,EPHA2,CXCL9,IFNGR1,HSPG2,TNFRSF1B,TNFRSF4,SDC1,SPP1,IL4R,UMOD,IL1R1,IL10,SLAMF1,IL22,CCL15*
GO:0006950	*Response to stress*	*0.18*	*0.0091*	*CD74,TNFRSF1A,LAG3,TGFBR3,HMOX1,TFPI2,PROC,PI3,IL17C,CFP,OMG,BST2,PDGFRA,LY9,NRP1,GAL,ERBB3,IGFBP4,IFNAR1,CRH,CXADR,CSF1R,IL10RB,TFF1,INHBB,IGFBP7,FOLR2,AXL,COL3A1,CXCL10,HAVCR2,CLU,CSF1,IL20RB,CD300C,SELE,PLAUR,ICOSLG,GFRAL,APOD,SIGLEC10,RET,EPHA2,TGFBR2,CXCL9,ULBP2,IFNGR1,CCN2,WFDC2,WFDC12,HSPG2,TNFRSF1B,HLA-E,TNFRSF4,SDC1,TNFRSF19,ACVRL1,FOLR1,SPP1,IL4R,UMOD,CST3,TF,IL1R1,PENK,FGFR2,IL10,SERPINE2,IL18BP,SLAMF1,IL22,PGF,CFD,SPON2,CCL15,B2M,FAS,CD59*
GO:0007165	*Signal transduction*	*0.16*	*0.0091*	*CD74,MYOC,TNFRSF17,TNFRSF1A,LAG3,TGFBR3,HMOX1,OLFM4,DKK4,IL17C,SLURP1,PDGFRA,CCN3,TNFRSF13B,FLT4,TNFRSF8,IL11,NRP1,GAL,CD27,ERBB3,IGFBP4,IFNAR1,GFRA3,CRH,EPHA4,THY1,CSF1R,NBL1,IL10RB,TFF2,TFF1,FGFR4,INHBB,AXL,IGFBP6,PRL,COL3A1,CXCL10,RGMB,HAVCR2,CLU,CD276,CSF1,IL20RB,SELE,PLAUR,ICOSLG,SERPINA12,GFRAL,GFRA1,RET,EPHB4,EPHA2,TGFBR2,CXCL9,DLL1,IFNGR1,CCN2,FCRL1,ROR1,CCN5,BAMBI,ITGBL1,TNFRSF1B,TNFRSF4,SDC1,TNFRSF19,ACVRL1,EFEMP1,SPP1,IL4R,UMOD,PDCD1LG2,TF,IL1R1,PENK,FGFR2,IL10,DKK3,IL22,PGF,TNFRSF11A,CFD,MIA,CCL15,FAS,CD59,IL19*
GO:0009967	*Positive regulation of signal transduction*	*0.27*	*0.0091*	*CD74,MYOC,TNFRSF1A,TGFBR3,HMOX1,BST2,PDGFRA,CCN3,FLT4,NID1,IL11,NRP1,CD27,ERBB3,CDH2,IGFBP4,EPHA4,CSF1R,IL10RB,FGFR4,INHBB,AXL,IGFBP6,PRL,COL3A1,HAVCR2,CLU,TNFRSF12A,CSF1,PLAUR,SERPINA12,GFRAL,RET,TGFBR2,DLL1,CCN2,ROR1,BAMBI,GPC5,TNFRSF19,ACVRL1,IL1R1,FGFR2,IL10,SLAMF1,TNFRSF11A,CCL15,FAS,IL19*
GO:0010033	*Response to organic substance*	*0.2*	*0.0091*	*CD74,TNFRSF17,TNFRSF1A,TGFBR3,HMOX1,BST2,PDGFRA,CDH1,FLT4,TIMP2,NRP1,LAMP3,GAL,IFNAR1,GFRA3,EPHA4,CSF1R,IL10RB,TFF2,TFF1,FGFR4,INHBB,IGFBP7,FOLR2,AXL,LY6D,COL3A1,CXCL10,RGMB,HAVCR2,CLU,CSF1,IL20RB,SELE,ICOSLG,GFRAL,GFRA1,RET,EPHA2,TGFBR2,CXCL9,IFNGR1,CCN2,PTGDS,BAMBI,TNFRSF1B,TNFRSF4,SDC1,TNFRSF19,ACVRL1,FOLR1,SPP1,IL4R,UMOD,PDCD1LG2,IL1R1,PENK,MSR1,FGFR2,IL10,IL18BP,IL22,PGF,TCL1A,TNFRSF11A,SPON2,CCL15,B2M,FAS*
GO:0048583	*Regulation of response to stimulus*	*0.17*	*0.0091*	*CD74,MYOC,CD22,TNFRSF1A,LAG3,TGFBR3,HMOX1,DKK4,PROC,TNN,CFP,BST2,PDGFRA,CCN3,FLT4,FCER2,NID1,IL11,NRP1,CD27,ERBB3,CDH2,IGFBP4,CRH,EPHA4,THY1,CSF1R,NBL1,IL10RB,FGFR4,INHBB,IGFBP7,AXL,IGFBP6,PRL,MZB1,COL3A1,CXCL10,HAVCR2,CLU,CD276,TNFRSF12A,CSF1,IL20RB,SELE,PDCD1,PLAUR,ICOSLG,DLK1,SERPINA12,GFRAL,APOD,SIGLEC10,RET,EPHA2,TGFBR2,CD300A,DLL1,CCN2,ROR1,BAMBI,TNFRSF1B,HLA-E,GPC5,TNFRSF19,ACVRL1,FOLR1,SPP1,IL4R,IL1R1,PENK,FGFR2,IL10,SERPINE2,DKK3,IL18BP,SLAMF1,PGF,TNFRSF11A,CFD,CCL15,B2M,FAS,CD59,IL19*

GO:0050896	*Response to stimulus*	*0.1*	*0.0091*	*CD74,MYOC,TNFRSF17,TNFRSF1A,LAG3,TGFBR3,HMOX1,OLFM4,DKK4,TFPI2,PROC,PI3,IL17C,SLURP1,CFP,OMG,BST2,PDGFRA,CCN3,TNFRSF13B,CDH1,FLT4,TIMP2,LY9,TNFRSF8,IL11,NRP1,LAMP3,GAL,CD27,ERBB3,CDH2,IGFBP4,IFNAR1,GFRA3,CRH,EPHA4,THY1,CXADR,CSF1R,NBL1,IL10RB,TFF2,TFF1,FGFR4,INHBB,IGFBP7,FOLR2,AXL,LY6D,IGFBP6,PRL,COL3A1,CXCL10,RGMB,HAVCR2,CLU,CD276,CSF1,IL20RB,CD300C,SELE,PDCD1,PLAUR,ICOSLG,SERPINA12,GFRAL,APOD,SIGLEC10,GFRA1,RET,EPHB4,EPHA2,TGFBR2,CXCL9,DLL1,ULBP2,IFNGR1,CCN2,FCRL1,ROR1,PTGDS,WFDC2,WFDC12,CCN5,HSPG2,BAMBI,ITGBL1,TNFRSF1B,HLA-E,TNFRSF4,SDC1,TNFRSF19,ACVRL1,FOLR1,EFEMP1,SPP1,IL4R,UMOD,PDCD1LG2,CST3,TF,IL1R1,PENK,MSR1,FGFR2,IL10,SERPINE2,DKK3,IL18BP,SLAMF1,IL22,PGF,TCL1A,TNFRSF11A,CFD,MIA,SPON2,CCL15,B2M,FAS,CD59,CRYBB2,IL19*
GO:0008285	*Negative regulation of cell population proliferation*	*0.36*	*0.0092*	*TGFBR3,HMOX1,TNN,SLURP1,CCN3,TNFRSF13B,TNFRSF8,GAL,CXADR,CSF1R,TFF1,IGFBP7,IGFBP6,PRL,HAVCR2,IL20RB,ICOSLG,APOD,TGFBR2,COL18A1,CD300A,DLL1,PTGDS,ACVRL1,UMOD,PDCD1LG2,FGFR2,IL10,SERPINE2,TNFRSF9,B2M*
GO:0048523	*Negative regulation of cellular process*	*0.17*	*0.0092*	*CD74,MYOC,CD22,TNFRSF1A,LAG3,TGFBR3,HMOX1,DKK4,PROC,TNN,SLURP1,BST2,PDGFRA,CCN3,TNFRSF13B,CDH1,FLT4,TIMP2,TNFRSF8,IL11,NRP1,LAMP3,GAL,CD27,ERBB3,CDH2,IGFBP4,CRH,EPHA4,THY1,CXADR,CSF1R,NBL1,TFF1,INHBB,IGFBP7,AXL,IGFBP6,PRL,COL3A1,CXCL10,HAVCR2,CLU,CSF1,IL20RB,PDCD1,PLAUR,ICOSLG,DLK1,SERPINA12,GFRAL,APOD,DNM3,EPHA2,TGFBR2,COL18A1,CD300A,DLL1,CCN2,PTGDS,CCN5,HSPG2,BAMBI,TNFRSF1B,HLA-E,TNFRSF4,ACVRL1,EFEMP1,SPP1,IL4R,UMOD,PDCD1LG2,CST3,JAM2,FGFR2,IL10,SERPINE2,DKK3,SLAMF1,TCL1A,TNFRSF9,B2M,FAS,IL19*
GO:0048584	*Positive regulation of response to stimulus*	*0.22*	*0.0092*	*CD74,MYOC,TNFRSF1A,LAG3,TGFBR3,HMOX1,CFP,BST2,PDGFRA,CCN3,FLT4,FCER2,NID1,IL11,NRP1,CD27,ERBB3,CDH2,IGFBP4,EPHA4,THY1,CSF1R,IL10RB,FGFR4,INHBB,AXL,IGFBP6,PRL,COL3A1,CXCL10,HAVCR2,CLU,CD276,TNFRSF12A,CSF1,PLAUR,ICOSLG,SERPINA12,GFRAL,RET,TGFBR2,DLL1,CCN2,ROR1,BAMBI,HLA-E,GPC5,TNFRSF19,ACVRL1,IL4R,IL1R1,PENK,FGFR2,IL10,SLAMF1,PGF,TNFRSF11A,CFD,CCL15,B2M,FAS,IL19*
GO:0002683	*Negative regulation of immune system process*	*0.41*	*0.0140*	*CD74,CD22,LAG3,HMOX1,BST2,CCN3,TNFRSF13B,GAL,THY1,NBL1,AXL,COL3A1,HAVCR2,IL20RB,PDCD1,ICOSLG,APOD,CD300A,DLL1,HLA-E,IL4R,PDCD1LG2,IL10,SLAMF1,CD59*
GO:0007167	*Enzyme-linked receptor protein signaling pathway*	*0.36*	*0.0173*	*MYOC,TGFBR3,PDGFRA,FLT4,NRP1,ERBB3,GFRA3,EPHA4,CSF1R,FGFR4,INHBB,AXL,COL3A1,RGMB,CSF1,GFRAL,GFRA1,RET,EPHB4,EPHA2,TGFBR2,CCN2,ROR1,BAMBI,ACVRL1,EFEMP1,TF,FGFR2,PGF*
GO:0006955	*Immune response*	*0.25*	*0.0178*	*CD74,TNFRSF17,LAG3,TGFBR3,PI3,CFP,BST2,TNFRSF13B,LY9,LAMP3,CD27,IFNAR1,CSF1R,IL10RB,AXL,CXCL10,HAVCR2,CLU,CSF1,IL20RB,PDCD1,ICOSLG,SIGLEC10,CXCL9,DLL1,ULBP2,IFNGR1,PTGDS,WFDC2,WFDC12,TNFRSF1B,HLA-E,TNFRSF4,IL4R,UMOD,PDCD1LG2,TF,IL1R1,IL10,IL18BP,SLAMF1,TNFRSF11A,CFD,SPON2,CCL15,B2M,FAS,IL19*
GO:0070663	*Regulation of leukocyte proliferation*	*0.44*	*0.0198*	*CD74,CD22,BST2,TNFRSF13B,GAL,CSF1R,MZB1,HAVCR2,CD276,CSF1,IL20RB,ICOSLG,TGFBR2,CD300A,TNFRSF1B,HLA-E,TNFRSF4,PDCD1LG2,IL10,SLAMF1,TNFRSF9*
GO:0001775	*Cell activation*	*0.32*	*0.0214*	*CD74,CD22,LAG3,SLURP1,BST2,PDGFRA,LY9,IL11,CXADR,AXL,LY6D,COL3A1,CXCL10,HAVCR2,CLU,CD276,CSF1,IL20RB,ICOSLG,TGFBR2,DLL1,ULBP2,IFNGR1,FCRL1,PTGDS,HLA-E,TNFRSF4,IL4R,IL10,SERPINE2,SLAMF1,B2M*
GO:0002700	*Regulation of production of molecular mediator of immune response*	*0.54*	*0.0214*	*CD74,CD22,HMOX1,BST2,AXL,MZB1,TNFRSF1B,HLA-E,TNFRSF4,IL4R,IL1R1,IL10,SLAMF1,SPON2,B2M*
GO:0007154	*Cell communication*	*0.14*	*0.0214*	*CD74,MYOC,TNFRSF17,TNFRSF1A,LAG3,TGFBR3,HMOX1,OLFM4,DKK4,IL17C,SLURP1,PDGFRA,CCN3,TNFRSF13B,FLT4,TNFRSF8,IL11,NRP1,GAL,CD27,ERBB3,IGFBP4,IFNAR1,GFRA3,CRH,EPHA4,THY1,CXADR,CSF1R,NBL1,IL10RB,TFF2,TFF1,FGFR4,INHBB,FOLR2,AXL,IGFBP6,PRL,COL3A1,CXCL10,RGMB,HAVCR2,CLU,CD276,CSF1,IL20RB,SELE,PLAUR,ICOSLG,SERPINA12,GFRAL,GFRA1,RET,EPHB4,EPHA2,TGFBR2,CXCL9,DLL1,IFNGR1,CCN2,FCRL1,ROR1,CCN5,BAMBI,ITGBL1,TNFRSF1B,TNFRSF4,SDC1,TNFRSF19,ACVRL1,FOLR1,EFEMP1,SPP1,IL4R,UMOD,PDCD1LG2,TF,IL1R1,PENK,FGFR2,IL10,DKK3,IL22,PGF,TNFRSF11A,CFD,MIA,CCL15,FAS,CD59,IL19*
GO:0009966	*Regulation of signal transduction*	*0.19*	*0.0214*	*CD74,MYOC,CD22,TNFRSF1A,TGFBR3,HMOX1,DKK4,TNN,BST2,PDGFRA,CCN3,FLT4,NID1,IL11,NRP1,CD27,ERBB3,CDH2,IGFBP4,CRH,EPHA4,THY1,CSF1R,NBL1,IL10RB,FGFR4,INHBB,IGFBP7,AXL,IGFBP6,PRL,MZB1,COL3A1,HAVCR2,CLU,TNFRSF12A,CSF1,PLAUR,DLK1,SERPINA12,GFRAL,APOD,RET,EPHA2,TGFBR2,CD300A,DLL1,CCN2,ROR1,BAMBI,GPC5,TNFRSF19,ACVRL1,FOLR1,IL1R1,FGFR2,IL10,SERPINE2,DKK3,IL18BP,SLAMF1,TNFRSF11A,CCL15,FAS,IL19*
GO:0010646	*Regulation of cell communication*	*0.18*	*0.0214*	*CD74,MYOC,CD22,TNFRSF1A,TGFBR3,HMOX1,DKK4,TNN,BST2,PDGFRA,CCN3,CDH1,FLT4,NID1,IL11,NRP1,GAL,CD27,ERBB3,CDH2,IGFBP4,CRH,EPHA4,THY1,CXADR,CSF1R,NBL1,IL10RB,FGFR4,INHBB,IGFBP7,AXL,IGFBP6,PRL,MZB1,COL3A1,HAVCR2,CLU,TNFRSF12A,CSF1,PLAUR,DLK1,SERPINA12,GFRAL,APOD,RET,EPHA2,TGFBR2,CD300A,DLL1,CCN2,ROR1,BAMBI,GPC5,TNFRSF19,ACVRL1,FOLR1,SPP1,IL1R1,PENK,FGFR2,IL10,SERPINE2,DKK3,IL18BP,SLAMF1,TNFRSF11A,CCL15,FAS,IL19*
GO:0016477	*Cell migration*	*0.3*	*0.0214*	*TGFBR3,PDGFRA,CCN3,VCAN,NRP1,CDH2,GFRA3,EPHA4,CXADR,FGFR4,AXL,IGFBP6,COL3A1,CXCL10,CSF1,SELE,GFRA1,RET,EPHB4,EPHA2,CXCL9,CCN2,BAMBI,ITGBL1,GPC5,SDC1,ACVRL1,FOLR1,UMOD,JAM2,IL10,SLAMF1,TNFRSF11A,CCL15*
GO:0019221	*Cytokine-mediated signaling pathway*	*0.39*	*0.0214*	*CD74,TNFRSF17,TNFRSF1A,IFNAR1,GFRA3,CSF1R,IL10RB,TFF2,CXCL10,CSF1,IL20RB,GFRAL,GFRA1,CXCL9,IFNGR1,TNFRSF1B,TNFRSF4,TNFRSF19,IL4R,UMOD,IL1R1,TNFRSF11A,CCL15,FAS*
GO:0023052	*Signaling*	*0.14*	*0.0214*	*CD74,MYOC,TNFRSF17,TNFRSF1A,LAG3,TGFBR3,HMOX1,OLFM4,DKK4,IL17C,SLURP1,PDGFRA,CCN3,TNFRSF13B,FLT4,TNFRSF8,IL11,NRP1,GAL,CD27,ERBB3,IGFBP4,IFNAR1,GFRA3,CRH,EPHA4,THY1,CXADR,CSF1R,NBL1,IL10RB,TFF2,TFF1,FGFR4,INHBB,AXL,IGFBP6,PRL,COL3A1,CXCL10,RGMB,HAVCR2,CLU,CD276,CSF1,IL20RB,SELE,PLAUR,ICOSLG,SERPINA12,GFRAL,GFRA1,RET,EPHB4,EPHA2,TGFBR2,CXCL9,DLL1,IFNGR1,CCN2,FCRL1,ROR1,CCN5,BAMBI,ITGBL1,TNFRSF1B,TNFRSF4,SDC1,TNFRSF19,ACVRL1,EFEMP1,SPP1,IL4R,UMOD,PDCD1LG2,TF,IL1R1,PENK,FGFR2,IL10,DKK3,IL22,PGF,TNFRSF11A,CFD,MIA,CCL15,FAS,CD59,IL19*
GO:0035295	*Tube development*	*0.32*	*0.0214*	*TGFBR3,HMOX1,PDGFRA,HS6ST1,CCN3,FLT4,NRP1,CDH2,CRH,EPHA4,THY1,CSF1R,INHBB,COL3A1,TNFRSF12A,CSF1,APOD,RET,EPHB4,EPHA2,TGFBR2,COL18A1,DLL1,CCN2,HSPG2,SDC1,ACVRL1,FOLR1,UMOD,FGFR2,SERPINE2,PGF*
GO:0048513	*Animal organ development*	*0.19*	*0.0214*	*CD74,MYOC,RELT,TNFRSF1A,TGFBR3,HMOX1,PDGFRA,HS6ST1,CCN3,TNFRSF13B,CDH1,FLT4,LY9,NID1,IL11,NRP1,ERBB3,CDH2,CRH,EPHA4,THY1,CXADR,CSF1R,NBL1,INHBB,COL6A3,AXL,LY6D,PRL,COL3A1,CXCL10,CRELD1,CSF1,APOD,GFRA1,RET,EPHB4,EPHA2,TGFBR2,MFAP5,COL18A1,DLL1,CCN2,ROR1,HSPG2,TNFRSF1B,SDC1,TNFRSF19,ACVRL1,FOLR1,EFEMP1,SPP1,IL4R,UMOD,TF,FGFR2,IL10,SERPINE2,DKK3,SLAMF1,PGF,TNFRSF11A,B2M,CRYBB2*
GO:0048514	*Blood vessel morphogenesis*	*0.4*	*0.0214*	*TGFBR3,HMOX1,PDGFRA,HS6ST1,CCN3,FLT4,NRP1,CDH2,THY1,COL3A1,TNFRSF12A,APOD,EPHB4,EPHA2,TGFBR2,COL18A1,DLL1,CCN2,HSPG2,ACVRL1,FOLR1,FGFR2,PGF*

GO:0051239	*Regulation of multicellular organismal process*	*0.19*	*0.0214*	*CD74,TNFRSF1A,LAG3,TGFBR3,HMOX1,DKK4,PROC,TNN,BST2,PDGFRA,CCN3,FLT4,LY9,TNFRSF8,NRP1,GAL,CD27,ERBB3,CRH,EPHA4,THY1,CXADR,CSF1R,TFF2,INHBB,AXL,PRL,CXCL10,HAVCR2,CLU,CD276,CSF1,IL20RB,PDCD1,PLAUR,ICOSLG,APOD,RET,EPHA2,TGFBR2,DLL1,THBS2,IFNGR1,CCN2,PTGDS,HSPG2,BAMBI,TNFRSF1B,HLA-E,ACVRL1,EFEMP1,SPP1,IL4R,PDCD1LG2,CST3,JAM2,TF,IL1R1,PENK,FGFR2,IL10,SERPINE2,SLAMF1,PGF,TNFRSF11A,SPON2,B2M,IL19*
GO:0051716	*Cellular response to stimulus*	*0.12*	*0.0214*	*CD74,MYOC,TNFRSF17,TNFRSF1A,LAG3,TGFBR3,HMOX1,OLFM4,DKK4,TFPI2,IL17C,SLURP1,OMG,PDGFRA,CCN3,TNFRSF13B,CDH1,FLT4,TNFRSF8,IL11,NRP1,GAL,CD27,ERBB3,IGFBP4,IFNAR1,GFRA3,CRH,EPHA4,THY1,CXADR,CSF1R,NBL1,IL10RB,TFF2,TFF1,FGFR4,INHBB,IGFBP7,FOLR2,AXL,IGFBP6,PRL,COL3A1,CXCL10,RGMB,HAVCR2,CLU,CD276,CSF1,IL20RB,SELE,PLAUR,ICOSLG,SERPINA12,GFRAL,APOD,GFRA1,RET,EPHB4,EPHA2,TGFBR2,CXCL9,DLL1,IFNGR1,CCN2,FCRL1,ROR1,CCN5,BAMBI,ITGBL1,TNFRSF1B,TNFRSF4,SDC1,TNFRSF19,ACVRL1,FOLR1,EFEMP1,SPP1,IL4R,UMOD,PDCD1LG2,TF,IL1R1,PENK,MSR1,FGFR2,IL10,DKK3,IL18BP,SLAMF1,IL22,PGF,TCL1A,TNFRSF11A,CFD,MIA,SPON2,CCL15,B2M,FAS,CD59,IL19*
GO:0071310	*Cellular response to organic substance*	*0.22*	*0.0214*	*CD74,TNFRSF17,TNFRSF1A,TGFBR3,PDGFRA,CDH1,FLT4,NRP1,IFNAR1,GFRA3,EPHA4,CSF1R,IL10RB,TFF2,FGFR4,INHBB,IGFBP7,FOLR2,AXL,COL3A1,CXCL10,RGMB,HAVCR2,CSF1,IL20RB,ICOSLG,GFRAL,GFRA1,RET,TGFBR2,CXCL9,IFNGR1,CCN2,BAMBI,TNFRSF1B,TNFRSF4,TNFRSF19,ACVRL1,FOLR1,SPP1,IL4R,UMOD,PDCD1LG2,IL1R1,PENK,MSR1,FGFR2,IL10,IL18BP,PGF,TCL1A,TNFRSF11A,SPON2,CCL15,B2M,FAS*
GO:0035239	*Tube morphogenesis*	*0.35*	*0.0240*	*TGFBR3,HMOX1,PDGFRA,HS6ST1,CCN3,FLT4,NRP1,CDH2,EPHA4,THY1,CSF1R,COL3A1,TNFRSF12A,CSF1,APOD,RET,EPHB4,EPHA2,TGFBR2,COL18A1,DLL1,CCN2,HSPG2,ACVRL1,FOLR1,FGFR2,PGF*
GO:0032944	*Regulation of mononuclear cell proliferation*	*0.44*	*0.0255*	*CD74,CD22,TNFRSF13B,GAL,MZB1,HAVCR2,CD276,CSF1,IL20RB,ICOSLG,TGFBR2,CD300A,TNFRSF1B,HLA-E,TNFRSF4,PDCD1LG2,IL10,SLAMF1,TNFRSF9*
GO:0002697	*Regulation of immune effector process*	*0.4*	*0.0266*	*CD74,CD22,LAG3,HMOX1,CFP,BST2,FCER2,AXL,MZB1,HAVCR2,IL20RB,CD300A,TNFRSF1B,HLA-E,TNFRSF4,IL4R,IL1R1,IL10,SLAMF1,SPON2,B2M,CD59*
GO:0030155	*Regulation of cell adhesion*	*0.3*	*0.0266*	*CD74,MYOC,LAG3,OLFM4,TNN,CDH1,NID1,NRP1,CD27,ERBB3,EPHA4,THY1,HAVCR2,CD276,CSF1,IL20RB,SELE,PLAUR,ICOSLG,APOD,RET,EPHA2,TGFBR2,CD300A,DLL1,HLA-E,ACVRL1,IL4R,PDCD1LG2,FBLN2,IL10,SERPINE2,SLAMF1,B2M*
GO:0050789	*Regulation of biological process*	*0.07*	*0.0266*	*CD74,MYOC,TNFRSF17,CD22,TNFRSF1A,LAG3,TGFBR3,HMOX1,OLFM4,DKK4,TFPI2,PROC,TNN,PI3,IL17C,SLURP1,CFP,BST2,PDGFRA,CCN3,TNFRSF13B,CDH1,FLT4,TIMP2,LY9,TNFRSF8,FCER2,NID1,IL11,NRP1,LAMP3,GAL,CD27,ERBB3,CDH2,IGFBP4,IFNAR1,GFRA3,CRH,EPHA4,THY1,CXADR,CSF1R,NBL1,IL10RB,TFF2,TFF1,CSTB,FGFR4,INHBB,COL6A3,IGFBP7,FOLR2,AXL,IGFBP6,PRL,MZB1,COL3A1,CXCL10,RGMB,HAVCR2,CLU,CD276,TNFRSF12A,CSF1,IL20RB,SELE,PDCD1,PLAUR,ICOSLG,DLK1,SERPINA12,GFRAL,APOD,SIGLEC10,DNM3,GFRA1,RET,EPHB4,EPHA2,TGFBR2,COL18A1,CD300A,CXCL9,DLL1,THBS2,IFNGR1,CCN2,FCRL1,ROR1,PTGDS,WFDC2,WFDC12,CCN5,HSPG2,BAMBI,ITGBL1,TNFRSF1B,HLA-E,GPC5,TNFRSF4,SDC1,TNFRSF19,ACVRL1,FOLR1,EFEMP1,SPP1,IL4R,UMOD,PDCD1LG2,CST3,JAM2,FBLN2,TF,IL1R1,PENK,MSR1,FGFR2,IL10,SERPINE2,TFF3,DKK3,IL18BP,SLAMF1,IL22,PGF,TCL1A,TNFRSF11A,CFD,MIA,TNFRSF9,SPON2,CCL15,B2M,FAS,CD59,IL19*
GO:0023051	*Regulation of signaling*	*0.17*	*0.0283*	*CD74,MYOC,CD22,TNFRSF1A,TGFBR3,HMOX1,DKK4,TNN,BST2,PDGFRA,CCN3,CDH1,FLT4,NID1,IL11,NRP1,GAL,CD27,ERBB3,CDH2,IGFBP4,CRH,EPHA4,THY1,CSF1R,NBL1,IL10RB,FGFR4,INHBB,IGFBP7,AXL,IGFBP6,PRL,MZB1,COL3A1,HAVCR2,CLU,TNFRSF12A,CSF1,PLAUR,DLK1,SERPINA12,GFRAL,APOD,RET,EPHA2,TGFBR2,CD300A,DLL1,CCN2,ROR1,BAMBI,GPC5,TNFRSF19,ACVRL1,FOLR1,SPP1,IL1R1,PENK,FGFR2,IL10,SERPINE2,DKK3,IL18BP,SLAMF1,TNFRSF11A,CCL15,FAS,IL19*
GO:0009605	*Response to external stimulus*	*0.18*	*0.0305*	*CD74,TNFRSF1A,LAG3,HMOX1,PI3,CFP,BST2,PDGFRA,CCN3,CDH1,LY9,TNFRSF8,NRP1,CDH2,IFNAR1,GFRA3,EPHA4,CXADR,CSF1R,IL10RB,INHBB,FOLR2,AXL,PRL,CXCL10,HAVCR2,CLU,CSF1,SELE,PLAUR,ICOSLG,GFRAL,SIGLEC10,RET,EPHB4,EPHA2,TGFBR2,CXCL9,ULBP2,IFNGR1,WFDC2,WFDC12,TNFRSF1B,HLA-E,FOLR1,SPP1,IL4R,UMOD,PDCD1LG2,TF,PENK,FGFR2,IL10,SERPINE2,IL18BP,SLAMF1,PGF,TNFRSF11A,CFD,SPON2,CCL15,B2M,FAS*
GO:0033209	*Tumor necrosis factor-mediated signaling pathway*	*0.77*	*0.0339*	*TNFRSF17,TNFRSF1A,TNFRSF1B,TNFRSF4,TNFRSF19,UMOD,TNFRSF11A,FAS*
GO:0002718	*Regulation of cytokine production involved in immune response*	*0.61*	*0.0367*	*CD74,HMOX1,BST2,AXL,TNFRSF1B,HLA-E,IL1R1,IL10,SLAMF1,SPON2,B2M*
GO:0045321	*Leukocyte activation*	*0.33*	*0.0367*	*CD74,CD22,LAG3,BST2,LY9,IL11,CXADR,AXL,LY6D,HAVCR2,CLU,CD276,CSF1,IL20RB,ICOSLG,TGFBR2,DLL1,ULBP2,IFNGR1,FCRL1,PTGDS,HLA-E,TNFRSF4,IL4R,IL10,SLAMF1,B2M*
GO:0048522	*Positive regulation of cellular process*	*0.13*	*0.0367*	*CD74,MYOC,TNFRSF1A,LAG3,TGFBR3,HMOX1,OLFM4,PROC,TNN,CFP,BST2,PDGFRA,CCN3,CDH1,FLT4,TNFRSF8,FCER2,NID1,IL11,NRP1,GAL,CD27,ERBB3,CDH2,IGFBP4,IFNAR1,CRH,EPHA4,THY1,CSF1R,NBL1,IL10RB,FGFR4,INHBB,FOLR2,AXL,IGFBP6,PRL,MZB1,COL3A1,CXCL10,RGMB,HAVCR2,CLU,CD276,TNFRSF12A,CSF1,SELE,PDCD1,PLAUR,ICOSLG,SERPINA12,GFRAL,DNM3,GFRA1,RET,EPHB4,EPHA2,TGFBR2,CD300A,CXCL9,DLL1,THBS2,IFNGR1,CCN2,ROR1,BAMBI,TNFRSF1B,HLA-E,GPC5,TNFRSF4,SDC1,TNFRSF19,ACVRL1,SPP1,IL4R,PDCD1LG2,JAM2,FBLN2,TF,IL1R1,MSR1,FGFR2,IL10,SERPINE2,SLAMF1,PGF,TCL1A,TNFRSF11A,CCL15,B2M,FAS,IL19*
GO:0007169	*Transmembrane receptor protein tyrosine kinase signaling pathway*	*0.39*	*0.0400*	*MYOC,PDGFRA,FLT4,NRP1,ERBB3,GFRA3,EPHA4,CSF1R,FGFR4,AXL,CSF1,GFRAL,GFRA1,RET,EPHB4,EPHA2,CCN2,ROR1,EFEMP1,FGFR2,PGF*
GO:0050670	*Regulation of lymphocyte proliferation*	*0.43*	*0.0416*	*CD74,CD22,TNFRSF13B,GAL,MZB1,HAVCR2,CD276,IL20RB,ICOSLG,TGFBR2,CD300A,TNFRSF1B,HLA-E,TNFRSF4,PDCD1LG2,IL10,SLAMF1,TNFRSF9*
GO:0002698	*Negative regulation of immune effector process*	*0.56*	*0.0444*	*CD22,HMOX1,BST2,AXL,HAVCR2,IL20RB,CD300A,HLA-E,IL4R,IL10,SLAMF1,CD59*
GO:0051249	*Regulation of lymphocyte activation*	*0.35*	*0.0493*	*CD74,CD22,LAG3,TNFRSF13B,GAL,CD27,THY1,AXL,MZB1,HAVCR2,CD276,IL20RB,ICOSLG,TGFBR2,CD300A,TNFRSF1B,HLA-E,TNFRSF4,IL4R,PDCD1LG2,IL10,SLAMF1,TNFRSF9,B2M*

GO:0004888	Transmembrane signaling receptor activity	0.49	0.0000	CD74,TNFRSF1A,LAG3,TGFBR3,PDGFRA,FLT4,TNFRSF8,NRP1,GAL,CD27,ERBB3,IFNAR1,GFRA3,EPHA4,CSF1R,IL10RB,FGFR4,AXL,CD300LG,IL20RB,CD300C,SELE,GFRAL,GFRA1,RET,EPHB4,EPHA2,TGFBR2,CD300A,IFNGR1,FCRL1,ROR1,TNFRSF1B,TNFRSF4,TNFRSF19,ACVRL1,EFEMP1,IL4R,IL1R1,FGFR2,SLAMF1,TNFRSF11A,FAS
GO:0038023	Signaling receptor activity	0.42	0.0000	CD74,TNFRSF17,TNFRSF1A,LAG3,TGFBR3,PDGFRA,TNFRSF13B,FLT4,TNFRSF8,NRP1,GAL,CD27,ERBB3,IFNAR1,GFRA3,EPHA4,CSF1R,IL10RB,FGFR4,FOLR2,AXL,RGMB,CD300LG,IL20RB,CD300C,SELE,PLAUR,GFRAL,GFRA1,RET,EPHB4,EPHA2,TGFBR2,CD300A,IFNGR1,FCRL1,ROR1,TNFRSF1B,TNFRSF4,TNFRSF19,ACVRL1,FOLR1,EFEMP1,IL4R,IL1R1,FGFR2,SLAMF1,TNFRSF11A,TNFRSF9,FAS
GO:0019199	Transmembrane receptor protein kinase activity	0.83	0.0000	TGFBR3,PDGFRA,FLT4,NRP1,ERBB3,EPHA4,CSF1R,FGFR4,AXL,RET,EPHB4,EPHA2,TGFBR2,ROR1,ACVRL1,EFEMP1,FGFR2
GO:0005102	Signaling receptor binding	0.27	0.0001	CD74,MYOC,CD22,ICAM3,LAG3,TGFBR3,DKK4,TNN,IL17C,SLURP1,PDGFRA,CCN3,FCER2,IL11,GAL,ERBB3,IGFBP4,GFRA3,CRH,EPHA4,THY1,CXADR,NBL1,TFF2,TFF1,INHBB,IGFBP6,PRL,COL3A1,CXCL10,CLU,CD276,CSF1,PLAUR,ICOSLG,GFRAL,DNM3,GFRA1,TGFBR2,CXCL9,DLL1,ULBP2,CCN2,CCN5,HSPG2,BAMBI,ITGBL1,HLA-E,EFEMP1,SPP1,JAM2,TF,IL1R1,PENK,IL10,ICAM2,SERPINE2,DKK3,IL18BP,IL22,PGF,MIA,CCL15,IL19
GO:0004714	Transmembrane receptor protein tyrosine kinase activity	0.8	0.0001	PDGFRA,FLT4,NRP1,ERBB3,EPHA4,CSF1R,FGFR4,AXL,RET,EPHB4,EPHA2,ROR1,EFEMP1,FGFR2
GO:0019955	Cytokine binding	0.63	0.0010	CD74,TNFRSF1A,TGFBR3,NRP1,IFNAR1,CSF1R,NBL1,IL20RB,TGFBR2,IFNGR1,TNFRSF1B,ACVRL1,IL1R1,IL18BP,TNFRSF11A,TNFRSF9
GO:0019838	Growth factor binding	0.57	0.0036	TGFBR3,PDGFRA,FLT4,NRP1,ERBB3,IGFBP4,FGFR4,IGFBP7,IGFBP6,COL3A1,EPHA2,TGFBR2,CCN2,ACVRL1,IL1R1,FGFR2
GO:0005031	Tumor necrosis factor receptor activity	1.12	0.0065	TNFRSF1A,TNFRSF1B,TNFRSF4,TNFRSF19,TNFRSF11A,FAS
GO:0005201	Extracellular matrix structural constituent	0.61	0.0140	TFPI2,MATN2,NID1,VCAN,COL6A3,COL3A1,MFAP5,COL18A1,COL24A1,HSPG2,UMOD,FBLN2
GO:0005178	Integrin binding	0.5	0.0196	ICAM3,TNN,CCN3,FCER2,THY1,CXADR,COL3A1,GFRA1,CCN2,CCN5,HSPG2,ITGBL1,SPP1,JAM2,ICAM2
GO:0071944	Cell periphery	0.21	0.0000	CD74,MYOC,TNFRSF17,RELT,CD22,ICAM3,TNFRSF1A,LAG3,TGFBR3,HMOX1,OLFM4,TFPI2,TNN,PI3,CFP,OMG,BST2,MATN2,CBLIF,PDGFRA,HS6ST1,CCN3,TNFRSF13B,CDH1,FLT4,TIMP2,LY9,TNFRSF8,FCER2,NID1,VCAN,NRP1,LAMP3,CD27,ERBB3,CDH2,IFNAR1,GFRA3,EPHA4,THY1,CXADR,CSF1R,CDH15,IL10RB,CSTB,FGFR4,INHBB,COL6A3,IGFBP7,FOLR2,AXL,LY6D,COL3A1,CXCL10,RGMB,HAVCR2,CLU,CD276,CD300LG,CRELD1,TNFRSF12A,CSF1,IL20RB,CD300C,MCEMP1,SELE,PDCD1,PLAUR,ICOSLG,SERPINA12,GFRAL,SIGLEC10,DNM3,GFRA1,RET,EPHB4,EPHA2,TGFBR2,MFAP5,COL18A1,CD300A,CXCL9,DLL1,THBS2,ULBP2,IFNGR1,CCN2,FCRL1,COL24A1,ROR1,CCN5,HSPG2,BAMBI,ITGBL1,TNFRSF1B,HLA-E,GPC5,TNFRSF4,SDC1,TNFRSF19,ACVRL1,FOLR1,EFEMP1,IL4R,UMOD,PDCD1LG2,CST3,JAM2,FBLN2,TF,IL1R1,PENK,PDZD2,MSR1,FGFR2,ICAM2,SERPINE2,HEPH,SLAMF1,TNFRSF11A,TNFRSF9,SPON2,MUC2,B2M,FAS,CD59
GO:0009986	Cell surface	0.4	0.0000	CD74,CD22,TNFRSF1A,LAG3,TGFBR3,TNN,BST2,CBLIF,PDGFRA,LY9,FCER2,LAMP3,CD27,CDH2,GFRA3,EPHA4,THY1,CSF1R,FOLR2,AXL,LY6D,CXCL10,HAVCR2,CLU,CD276,SELE,PDCD1,PLAUR,ICOSLG,GFRAL,GFRA1,EPHA2,TGFBR2,CXCL9,ULBP2,FCRL1,ROR1,LAYN,HLA-E,GPC5,TNFRSF4,SDC1,ACVRL1,FOLR1,IL4R,UMOD,PDCD1LG2,JAM2,TF,IL1R1,MSR1,FGFR2,SERPINE2,SLAMF1,TNFRSF11A,TNFRSF9,B2M,FAS,CD59
GO:0009897	External side of plasma membrane	0.52	0.0000	CD74,CD22,LAG3,TGFBR3,CBLIF,PDGFRA,LY9,FCER2,CD27,GFRA3,THY1,FOLR2,CXCL10,CD276,SELE,PDCD1,ICOSLG,GFRAL,GFRA1,TGFBR2,CXCL9,ULBP2,HLA-E,TNFRSF4,SDC1,FOLR1,IL4R,PDCD1LG2,TF,IL1R1,MSR1,SERPINE2,SLAMF1,TNFRSF11A,TNFRSF9,B2M,FAS,CD59
GO:0031224	Intrinsic component of membrane	0.23	0.0000	CD74,TNFRSF17,RELT,CD22,ICAM3,TNFRSF1A,LAG3,TGFBR3,HMOX1,OMG,BST2,PDGFRA,HS6ST1,TNFRSF13B,CDH1,FLT4,LY9,TNFRSF8,FCER2,NRP1,LAMP3,CD27,ERBB3,CDH2,IFNAR1,GFRA3,EPHA4,THY1,CXADR,CSF1R,CDH15,IL10RB,FGFR4,FOLR2,AXL,LY6D,RGMB,HAVCR2,CD276,CD300LG,CRELD1,TNFRSF12A,CSF1,IL20RB,CD300C,MCEMP1,SELE,PDCD1,PLAUR,ICOSLG,DLK1,GFRAL,SIGLEC10,GFRA1,RET,EPHB4,EPHA2,TGFBR2,CD300A,DLL1,ULBP2,IFNGR1,FCRL1,ROR1,BAMBI,LAYN,TNFRSF1B,HLA-E,GPC5,TNFRSF4,SDC1,TNFRSF19,ACVRL1,FOLR1,IL4R,UMOD,PDCD1LG2,JAM2,IL1R1,PRRT3,MSR1,FGFR2,ICAM2,HS6ST2,HEPH,SLAMF1,TNFRSF11A,TNFRSF9,B2M,FAS,CD59
GO:0005886	Plasma membrane	0.19	0.0000	CD74,TNFRSF17,RELT,CD22,ICAM3,TNFRSF1A,LAG3,TGFBR3,HMOX1,OLFM4,PI3,CFP,OMG,BST2,CBLIF,PDGFRA,HS6ST1,TNFRSF13B,CDH1,FLT4,LY9,TNFRSF8,FCER2,NID1,NRP1,LAMP3,CD27,ERBB3,CDH2,IFNAR1,GFRA3,EPHA4,THY1,CXADR,CSF1R,CDH15,IL10RB,FGFR4,COL6A3,FOLR2,AXL,LY6D,CXCL10,RGMB,HAVCR2,CD276,CD300LG,TNFRSF12A,CSF1,IL20RB,CD300C,MCEMP1,SELE,PDCD1,PLAUR,ICOSLG,SERPINA12,GFRAL,SIGLEC10,DNM3,GFRA1,RET,EPHB4,EPHA2,TGFBR2,CD300A,CXCL9,DLL1,ULBP2,IFNGR1,CCN2,FCRL1,ROR1,HSPG2,BAMBI,ITGBL1,TNFRSF1B,HLA-E,GPC5,TNFRSF4,SDC1,TNFRSF19,ACVRL1,FOLR1,IL4R,UMOD,PDCD1LG2,CST3,JAM2,TF,IL1R1,PENK,PDZD2,MSR1,FGFR2,ICAM2,SERPINE2,HEPH,SLAMF1,TNFRSF11A,TNFRSF9,MUC2,B2M,FAS,CD59
GO:0098552	Side of membrane	0.43	0.0000	CD74,CD22,LAG3,TGFBR3,CBLIF,PDGFRA,CDH1,LY9,FCER2,CD27,GFRA3,THY1,FOLR2,CXCL10,CD276,SELE,PDCD1,ICOSLG,GFRAL,GFRA1,TGFBR2,CXCL9,ULBP2,HLA-E,TNFRSF4,SDC1,FOLR1,IL4R,PDCD1LG2,TF,IL1R1,MSR1,SERPINE2,SLAMF1,TNFRSF11A,TNFRSF9,B2M,FAS,CD59
GO:0005576	Extracellular region	0.16	0.0000	CD74,MYOC,CD22,ICAM3,TNFRSF1A,LAG3,TGFBR3,HMOX1,OLFM4,DKK4,TFPI2,PROC,TNN,PI3,IL17C,SLURP1,CFP,BST2,MATN2,CBLIF,CCN3,CDH1,FLT4,TIMP2,TNFRSF8,FCER2,NID1,IL11,VCAN,NRP1,GAL,CD27,ERBB3,IGFBP4,CRH,THY1,CXADR,CDH15,NBL1,TFF2,TFF1,CSTB,FGFR4,INHBB,COL6A3,IGFBP7,FOLR2,AXL,LY6D,IGFBP6,PRL,MZB1,COL3A1,CXCL10,CLU,CSF1,SELE,PLAUR,ICOSLG,DLK1,SERPINA12,APOD,SIGLEC10,DNM3,GFRA1,EPHB4,TGFBR2,MFAP5,COL18A1,CD300A,CXCL9,DLL1,THBS2,ULBP2,CCN2,COL24A1,PTGDS,WFDC2,WFDC12,CCN5,HSPG2,ITGBL1,TNFRSF1B,HLA-E,GPC5,SDC1,FOLR1,EFEMP1,SPP1,IL4R,UMOD,PDCD1LG2,CST3,FBLN2,TF,IL1R1,PENK,PDZD2,MSR1,FGFR2,IL10,SERPINE2,TFF3,DKK3,IL18BP,SLAMF1,IL22,PGF,CFD,MIA,SPON2,CCL15,MUC2,B2M,FAS,CD59,IL19
GO:0016021	Integral component of membrane	0.22	0.0000	CD74,TNFRSF17,RELT,CD22,ICAM3,TNFRSF1A,LAG3,TGFBR3,HMOX1,BST2,PDGFRA,HS6ST1,TNFRSF13B,CDH1,FLT4,LY9,TNFRSF8,FCER2,NRP1,LAMP3,CD27,ERBB3,CDH2,IFNAR1,EPHA4,THY1,CXADR,CSF1R,CDH15,IL10RB,FGFR4,AXL,HAVCR2,CD276,CD300LG,CRELD1,TNFRS

				F12A,CSF1,IL20RB,CD300C,MCEMP1,SELE,PDCD1,PLAUR,ICOSLG,DLK1,GFRAL,SIGLEC10,RET,EPHB4,EPHA2,TGFBR2,CD300A,DLL1,IFNGR1,FCRL1,ROR1,BAMBI,LAYN,TNFRSF1B,HLA-E,GPC5,TNFRSF4,SDC1,TNFRSF19,ACVRL1,FOLR1,IL4R,UMOD,PDCD1LG2,JAM2,IL1R1,PRRT3,MSR1,FGFR2,ICAM2,HS6ST2,HEPH,SLAMF1,TNFRSF11A,TNFRSF9,B2M,FAS
GO:0043235	Receptor complex	0.46	0.0004	CD74,TNFRSF1A,TGFBR3,PDGFRA,FLT4,NRP1,ERBB3,GFRA3,EPHA4,CSF1R,IL10RB,FGFR4,AXL,GFRAL,GFRA1,RET,EPHB4,EPHA2,TGFBR2,ROR1,TNFRSF1B,ACVRL1,IL4R,TF,FGFR2,B2M
GO:0031226	Intrinsic component of plasma membrane	0.29	0.0004	CD22,ICAM3,TNFRSF1A,TGFBR3,PDGFRA,HS6ST1,TNFRSF13B,FLT4,FCER2,NRP1,CD27,ERBB3,CDH2,IFNAR1,EPHA4,THY1,CXADR,CSF1R,IL10RB,FGFR4,FOLR2,AXL,RGMB,IL20RB,CD300C,SELE,PLAUR,RET,EPHB4,EPHA2,TGFBR2,DLL1,ULBP2,IFNGR1,FCRL1,ROR1,GPC5,TNFRSF4,SDC1,ACVRL1,FOLR1,IL4R,JAM2,IL1R1,MSR1,FGFR2,ICAM2,TNFRSF9,CD59
GO:0005615	Extracellular space	0.15	0.0015	CD74,MYOC,CD22,ICAM3,TNFRSF1A,TGFBR3,HMOX1,OLFM4,DKK4,TFPI2,PROC,TNN,PI3,IL17C,SLURP1,CFP,BST2,CBLIF,CDH1,TIMP2,TNFRSF8,FCER2,NID1,IL11,VCAN,NRP1,GAL,ERBB3,IGFBP4,CRH,THY1,CXADR,CDH15,NBL1,TFF2,TFF1,CSTB,INHBB,COL6A3,IGFBP7,AXL,IGFBP6,PRL,COL3A1,CXCL10,CLU,CSF1,SELE,ICOSLG,DLK1,SERPINA12,APOD,DNM3,GFRA1,EPHB4,TGFBR2,COL18A1,CD300A,CXCL9,ULBP2,CCN2,COL24A1,PTGDS,WFDC2,WFDC12,CCN5,HSPG2,HLA-E,GPC5,SDC1,FOLR1,EFEMP1,SPP1,IL4R,UMOD,CST3,TF,MSR1,IL10,SERPINE2,TFF3,DKK3,IL18BP,SLAMF1,IL22,PGF,CFD,MIA,SPON2,CCL15,MUC2,B2M,FAS,CD59,IL19
GO:0005887	Integral component of plasma membrane	0.27	0.0046	CD22,ICAM3,TNFRSF1A,TGFBR3,PDGFRA,HS6ST1,TNFRSF13B,FLT4,FCER2,NRP1,CD27,ERBB3,CDH2,IFNAR1,EPHA4,THY1,CXADR,CSF1R,IL10RB,FGFR4,AXL,IL20RB,CD300C,SELE,PLAUR,RET,EPHB4,EPHA2,TGFBR2,DLL1,IFNGR1,FCRL1,ROR1,TNFRSF4,SDC1,ACVRL1,IL4R,JAM2,IL1R1,MSR1,FGFR2,ICAM2,TNFRSF9
GO:0031012	Extracellular matrix	0.34	0.0057	MYOC,TGFBR3,TFPI2,TNN,PI3,MATN2,CCN3,TIMP2,NID1,VCAN,CDH2,CSTB,COL6A3,IGFBP7,COL3A1,CLU,CRELD1,MFAP5,COL18A1,THBS2,CCN2,COL24A1,CCN5,HSPG2,GPC5,EFEMP1,FBLN2,SERPINE2,SPON2,MUC2
GO:0016020	Membrane	0.09	0.0174	CD74,MYOC,TNFRSF17,RELT,CD22,ICAM3,TNFRSF1A,LAG3,TGFBR3,HMOX1,OLFM4,PI3,CFP,OMG,BST2,CBLIF,PDGFRA,HS6ST1,TNFRSF13B,CDH1,FLT4,LY9,TNFRSF8,FCER2,NID1,VCAN,NRP1,LAMP3,CD27,ERBB3,CDH2,IFNAR1,GFRA3,EPHA4,THY1,CXADR,CSF1R,CDH15,IL10RB,FGFR4,COL6A3,FOLR2,AXL,LY6D,CXCL10,RGMB,HAVCR2,CLU,CD276,CD300LG,CRELD1,TNFRSF12A,CSF1,IL20RB,CD300C,MCEMP1,SELE,PDCD1,PLAUR,ICOSLG,DLK1,SERPINA12,GFRAL,SIGLEC10,DNM3,GFRA1,RET,EPHB4,EPHA2,TGFBR2,CD300A,CXCL9,DLL1,ULBP2,IFNGR1,CCN2,FCRL1,ROR1,PTGDS,HSPG2,BAMBI,LAYN,ITGBL1,TNFRSF1B,HLA-E,GPC5,TNFRSF4,SDC1,TNFRSF19,ACVRL1,FOLR1,IL4R,UMOD,PDCD1LG2,CST3,JAM2,TF,IL1R1,PRRT3,PENK,PDZD2,MSR1,FGFR2,ICAM2,SERPINE2,HS6ST2,HEPH,SLAMF1,PGF,TNFRSF11A,TNFRSF9,MUC2,B2M,FAS,CD59
GO:0031225	Anchored component of membrane	0.49	0.0217	OMG,BST2,GFRA3,THY1,FOLR2,LY6D,RGMB,PLAUR,GFRA1,ULBP2,GPC5,FOLR1,UMOD,CD59
GO:0005788	Endoplasmic reticulum lumen	0.4	0.0232	PROC,CFP,VCAN,CDH2,IGFBP4,COL6A3,IGFBP7,MZB1,COL3A1,CLU,CSF1,PLAUR,COL18A1,COL24A1,SPP1,CST3,TF,PENK,B2M
GO:0062023	Collagen-containing extracellular matrix	0.33	0.0304	MYOC,TNN,MATN2,CCN3,TIMP2,NID1,VCAN,CDH2,CSTB,COL6A3,IGFBP7,COL3A1,CLU,CRELD1,MFAP5,COL18A1,THBS2,COL24A1,HSPG2,GPC5,EFEMP1,FBLN2,SERPINE2,MUC2
CL:15538	Mixed, incl. Adaptive immunity, and TNFs bind their physiological receptors	0.65	0.0030	CD22,ICAM3,LAG3,LY9,TNFRSF8,LAMP3,CD27,THY1,HAVCR2,CD276,PDCD1,ICOSLG,TNFRSF4,PDCD1LG2,ICAM2,SLAMF1,TNFRSF9
CL:15539	Mixed, incl. T cell modulation in pancreatic cancer, and non-Langerhans-cell histiocytosis	0.67	0.0030	CD22,ICAM3,LAG3,TNFRSF8,LAMP3,CD27,THY1,HAVCR2,CD276,PDCD1,ICOSLG,TNFRSF4,PDCD1LG2,ICAM2,TNFRSF9
CL:15535	Mixed, incl. Chemokine-mediated signaling pathway, and Adaptive immunity	0.49	0.0061	CD22,ICAM3,LAG3,LY9,TNFRSF8,LAMP3,CD27,THY1,CXCL10,HAVCR2,CD276,SELE,PDCD1,ICOSLG,CXCL9,TNFRSF4,PDCD1LG2,ICAM2,SLAMF1,TNFRSF9,CCL15
CL:15536	Mixed, incl. Chemokine-mediated signaling pathway, and Adaptive immunity	0.49	0.0078	CD22,ICAM3,LAG3,LY9,TNFRSF8,LAMP3,CD27,THY1,CXCL10,HAVCR2,CD276,PDCD1,ICOSLG,CXCL9,TNFRSF4,PDCD1LG2,ICAM2,SLAMF1,TNFRSF9,CCL15
CL:15620	T cell modulation in pancreatic cancer, and TNFs bind their physiological receptors	0.77	0.0107	LAG3,TNFRSF8,CD27,HAVCR2,CD276,PDCD1,ICOSLG,TNFRSF4,PDCD1LG2,TNFRSF9
CL:19457	Extracellular matrix organization	0.53	0.0305	TGFBR3,TNN,TIMP2,NID1,COL6A3,COL3A1,TGFBR2,MFAP5,COL18A1,THBS2,COL24A1,HSPG2,EFEMP1,SPP1,FBLN2
CL:15622	TNFs bind their physiological receptors, and T cell modulation in pancreatic cancer	0.81	0.0316	TNFRSF8,CD27,CD276,PDCD1,ICOSLG,TNFRSF4,PDCD1LG2,TNFRSF9
CL:15625	T cell modulation in pancreatic cancer, and Lymphoproliferative syndrome 2	0.89	0.0316	CD27,CD276,PDCD1,ICOSLG,TNFRSF4,PDCD1LG2,TNFRSF9

hsa04060	Cytokine-cytokine receptor interaction	0.52	0.0000	TNFRSF17,RELT,TNFRSF1A,IL17C,TNFRSF13B,TNFRSF8,IL11,CD27,IFNAR1,CSF1R,IL10RB,INHBB,PRL,CXCL10,TNFRSF12A,CSF1,IL20RB,TGFBR2,CXCL9,IFNGR1,TNFRSF1B,TNFRSF4,TNFRSF19,ACVRL1,IL4R,IL1R1,IL10,IL22,TNFRSF11A,TNFRSF9,CCL15,FAS,IL19
hsa04514	Cell adhesion molecules	0.52	0.0332	CD22,ICAM3,CDH1,VCAN,CDH2,CDH15,CD276,SELE,PDCD1,HLA-E,SDC1,PDCD1LG2,JAM2,ICAM2
HSA-1280215	Cytokine Signaling in Immune system	0.36	0.0150	TNFRSF17,TNFRSF1A,PSMD5,HMOX1,IL17C,BST2,TNFRSF13B,TNFRSF8,FCER2,IL11,CD27,IFNAR1,CSF1R,IL10RB,PRL,CXCL10,HAVCR2,TNFRSF12A,CSF1,IL20RB,IFNGR1,TNFRSF1B,HLA-E,TNFRSF4,SDC1,IL4R,IL1R1,IL10,IL18BP,IL22,TNFRSF11A,TNFRSF9,B2M,IL19
HSA-168256	Immune System	0.21	0.0291	CD74,TNFRSF17,CD22,ICAM3,TNFRSF1A,LAG3,PSMD5,HMOX1,OLFM4,PI3,IL17C,CFP,BST2,TNFRSF13B,CDH1,TIMP2,TNFRSF8,FCER2,IL11,CD27,IFNAR1,CXADR,CSF1R,IL10RB,CSTB,PRL,COL3A1,CXCL10,HAVCR2,CLU,CD300LG,TNFRSF12A,CSF1,IL20RB,CD300C,MCEMP1,PDCD1,PLAUR,SIGLEC10,DNM3,CD300A,IFNGR1,TNFRSF1B,HLA-E,TNFRSF4,SDC1,IL4R,PDCD1LG2,CST3,IL1R1,IL10,ICAM2,IL18BP,IL22,TNFRSF11A,CFD,TNFRSF9,MUC2,B2M,CD59,IL19
HSA-5668541	TNFR2 non-canonical NF-kB pathway	0.7	0.0291	TNFRSF17,TNFRSF1A,PSMD5,TNFRSF13B,TNFRSF8,CD27,TNFRSF12A,TNFRSF1B,TNFRSF4,TNFRSF11A,TNFRSF9
HSA-5669034	TNFs bind their physiological receptors	0.85	0.0298	TNFRSF17,TNFRSF1A,TNFRSF13B,TNFRSF8,CD27,TNFRSF1B,TNFRSF4,TNFRSF9
**Cluster 2**				
GO:0098982	GABA-ergic synapse	1.51	0.0083	CBLN4,NLGN2,SLITRK2,SLITRK1
GO:0008076	Voltage-gated potassium channel complex	1.83	0.0106	CNTNAP2,KCNIP4,DPP10
GO:0016021	Integral component of membrane	0.42	0.0106	STX1B,SYT1,NLGN2,NAGPA,SLITRK2,PODXL2,KIRREL1,CNTNAP2,SLITRK1,KIAA0319,KCNIP4,DPP10,CNTNAP4
GO:0030054	Cell junction	0.6	0.0106	CBLN4,STX1B,SYT1,NLGN2,SLITRK2,KIRREL1,CNTNAP2,SLITRK1,CNTNAP4,NEFL
GO:0042734	Presynaptic membrane	1.35	0.0106	STX1B,SYT1,NLGN2,CNTNAP4
GO:0045202	Synapse	0.76	0.0106	CBLN4,STX1B,SYT1,NLGN2,SLITRK2,SLITRK1,CNTNAP4,NEFL
GO:0097060	Synaptic membrane	1.11	0.0106	STX1B,SYT1,NLGN2,SLITRK1,CNTNAP4
GO:0098793	Presynapse	0.94	0.0190	STX1B,SYT1,NLGN2,CNTNAP4,NEFL
GO:0043005	Neuron projection	0.66	0.0375	STX1B,SYT1,NLGN2,KIRREL1,CNTNAP2,KCNIP4,NEFL
HSA-6794362	Protein-protein interactions at synapses	1.65	0.0050	SYT1,NLGN2,SLITRK2,SLITRK1
HSA-112316	Neuronal System	1.23	0.0087	SYT1,NLGN2,SLITRK2,SLITRK1,NEFL
**Cluster 3**				
GO:0006629	Lipid metabolic process	0.98	0.0487	ENPP6,GPD1,ADH1B,PLB1,PLA2G2A,ALDH3A1
GO:0046486	Glycerolipid metabolic process	1.4	0.0487	ENPP6,GPD1,PLB1,PLA2G2A
GO:0016616	Oxidoreductase activity, acting on the CH-OH group of donors, NAD or NADP as acceptor	1.75	0.0309	GPD1,ADH1B,ALDH3A1
hsa00565	Ether lipid metabolism	2.03	0.0013	ENPP6,PLB1,PLA2G2A
hsa00564	Glycerophospholipid metabolism	1.86	0.0018	GPD1,PLB1,PLA2G2A
hsa00591	Linoleic acid metabolism	2.16	0.0153	PLB1,PLA2G2A
hsa00592	alpha-Linolenic acid metabolism	2.01	0.0196	PLB1,PLA2G2A
hsa00010	Glycolysis / Gluconeogenesis	1.74	0.0239	ADH1B,ALDH3A1
hsa00350	Tyrosine metabolism	1.86	0.0239	ADH1B,ALDH3A1
hsa00590	Arachidonic acid metabolism	1.81	0.0239	PLB1,PLA2G2A

hsa00980	*Metabolism of xenobiotics by cytochrome P450*	*1.81*	*0.0239*	*ADH1B,ALDH3A1*
hsa00982	*Drug metabolism - cytochrome P450*	*1.9*	*0.0239*	*ADH1B,ALDH3A1*
hsa01100	*Metabolic pathways*	*0.79*	*0.0239*	*ADH1B,PHYKPL,PLB1,PLA2G2A,ALDH3A1*
hsa05204	*Chemical carcinogenesis*	*1.71*	*0.0239*	*ADH1B,ALDH3A1*
HSA-1483257	*Phospholipid metabolism*	*1.76*	*0.0010*	*ENPP6,GPD1,PLB1,PLA2G2A*
HSA-1430728	*Metabolism*	*0.79*	*0.0069*	*ENPP6,GPD1,ADH1B,PHYKPL,PLB1,PLA2G2A,ALDH3A1*
HSA-1483206	*Glycerophospholipid biosynthesis*	*1.8*	*0.0089*	*GPD1,PLB1,PLA2G2A*
Cluster 4				
Cluster 5				
GO:0042582	*Azurophil granule*	*1.53*	*0.0338*	*GM2A,LAMP2,NPC2*
GO:0043202	*Lysosomal lumen*	*1.57*	*0.0338*	*GM2A,LAMP2,NPC2*
Cluster 6				
CL:19320	*Mixed, incl. Serine proteases, trypsin family, histidine active site, and Carboxypeptidase activation peptide*	*2.18*	*0.0008*	*CPA1,CELA2A,GP2*
CL:19328	*Carboxypeptidase activation peptide, and Pancreatitis*	*2.38*	*0.0229*	*CPA1,CELA2A*
Cluster 7				
hsa05032	*Morphine addiction*	*2.5*	*0.0066*	*GNGT1,GRK5*
Cluster 8				
CL:38327	*Mixed, incl. Chordin-like protein 1/2, and Positive regulation of corticotropin-releasing hormone secretion*	*2.8*	*0.0153*	*CHRDL1,MAMDC2*
SM00137	*Domain in meprin, A5, receptor protein tyrosine phosphatase mu (and others)*	*2.28*	*0.0164*	*MAMDC2,PTPRM*
Cluster 9				
GO:0004089	*Carbonate dehydratase activity*	*2.34*	*0.0371*	*CA12,CA4*
CL:12701	*Reversible hydration of carbon dioxide, and Essential fructosuria*	*2.5*	*0.0281*	*CA12,CA4*
hsa00910	*Nitrogen metabolism*	*2.38*	*0.0072*	*CA12,CA4*
HSA-1475029	*Reversible hydration of carbon dioxide*	*2.42*	*0.0322*	*CA12,CA4*
IPR001148	*Alpha carbonic anhydrase domain*	*2.31*	*0.0317*	*CA12,CA4*
IPR018338	*Carbonic anhydrase, alpha-class, conserved site*	*2.42*	*0.0317*	*CA12,CA4*

IPR023561	*Carbonic anhydrase, alpha-class*	*2.34*	*0.0317*	*CA12,CA4*
IPR036398	*Alpha carbonic anhydrase domain superfamily*	*2.31*	*0.0317*	*CA12,CA4*
SM01057	*Eukaryotic-type carbonic anhydrase*	*2.31*	*0.0100*	*CA12,CA4*

Table S 4: Results from KEGG & GO pathway enrichment analysis in symptomatic vs. asymptomatic DS.

Pathway	ES	NES	P Value	FDR P Value	Enriched Proteins
**KEGG**					
KEGG_FC_GAMMA_R_MEDIATED_PHAGOCYTOSIS	-0.5921	-1.9173	0.0010	0.0276	WASF3/VASP/AKT3/VAV3/DNM1/CRKL/PLA2G4A/INPP5D/WAS/ARF6/AKT2/PIKFYVE/WASL
KEGG_CYTOKINE_CYTOKINE_RECEPTOR_INTERACTION	0.39777593	1.76691117	<0.0000	0.0020	EDA2R/IL11/IL6/IFNL2/CCL18/CNTF/CD27/CCL24/CCL27/INHBB/CCL11/TNFRSF11B/VEGFB/IL22RA1/IL5RA/NGFR/CCL7/IL4R/TNFRSF12A/TNFRSF10B/IL17RB/IL15RA/CXCL13/IL2/VEGFD/TNFRSF11A/HGF/TNFRSF10A/FAS/IL1RAP/VEGFA/TGFB1/TNFSF13/TNFRSF1A/CXCL14/CCL2/CCL23/IFNL1/PDGFRA/CCL20/CXCL11/CCL8/CXCL9/TNFRSF17/CXCL16/CCL16/TGFBR2/CCL19/CCL14/XCL1/TNFRSF6B/CCL25/KITLG/INHBC/IL18R1/LEP/IL2RB/LEPR/TNFRSF13B/LTBR/CCL3/PDGFRB/IL1A/CCL15/IFNGR1/RELT/CXCL8/ACVRL1/OSMR/LIFR/TNFRSF1B/CCL21/CX3CL1/CXCL12/CD70/CXCL10/TNFRSF10C/IL20RA/IL15/CSF3R/CCL13/TNFRSF14/PDGFB
KEGG_ANTIGEN_PROCESSING_AND_PRESENTATION	0.6126709	1.9152887	0.0009	0.0276	KIR2DL3/KIR2DL2/CTSB/HLA-DRA/KIR3DL2/TAP1/HLA-A/KLRD1/CTSL/CD74/CD4/PDIA3/CD8A/KLRC1/B2M/PSME1/KIR2DS4
KEGG_GRAFT_VERSUS_HOST_DISEASE	0.67350914	1.95448596	0.0003	0.0142	KIR2DL3/KIR2DL2/IL6/HLA-DRA/KIR3DL2/HLA-A/KLRD1/IL2/FAS
**GO**					
GOMF_EXTRACELLULAR_MATRIX_STRUCTURAL_CONSTITUENT	0.5463	2.1085	0.0000	0.0002	CHI3L1/ELN/CCN1/LTBP2/VWF/SRPX/GP2/CILP/SPON1/PCOLCE/EFEMP1/COMP/ASPN/OPTC/PRG2/OGN/CTHRC1/COL6A3/DPT/FBLN2/MFAP4/NID2/TFPI2/AGRN/DCN/VWA1/TINAGL1/COL4A4/MFAP5/VCAN/FGA/CD4/IGFBP7/TNC/HSPG2/PRELP/EDIL3/MATN2
GOMF_GLYCOSAMINOGLYCAN_BINDING	0.4772	2.0230	0.0000	0.0001	LPA/ADGRG1/CCN1/LTBP2/TREM2/SMOC1/AOC1/SFRP1/PCOLCE/REG1B/LXN/CCN5/FGFBP3/REG1A/VEGFB/SUSD5/COMP/ANGPTL3/RSPO1/REG3A/HMMR/REG3G/CCL7/THBS4/PRG2/CXCL13/LAYN/PTN/VIT/VEGFA/CCN3/ADA2/PGF/MDK/EGFLAM/NRP2/AGRN/DCN/CCL23/PGLYRP4/REG4/C1QBP/CXCL11/NELL1/CCL8/CCDC80/PTPRF/TGFBR2/SMOC2/VCAN/ADAMTS1/TGFBR3/LRPAP1/HRG/PGLYRP2/NRP1/CCL15/PRELP/CXCL8/LPL/CCN4/RSPO3/POSTN/CXCL10/SOST/FGF9/FGFBP1/PDCD5/FN1/FSTL1/BGN/ADAMTS15/ACAN
GOCC_EXTERNAL_ENCAPSULATING_STRUCTURE	0.4386	2.0206	0.0000	0.0000	CHI3L1/ELN/TGM2/CCN1/LTBP2/GDF15/VWF/TIMP3/CTSB/SRPX/PLAT/TIMP4/SMOC1/CILP/SPON1/SFRP1/PCOLCE/PRSS2/ANGPTL2/EFEMP1/ADAMTS16/MBL2/LEFTY2/TNFRSF11B/CCN5/FGFBP3/COMP/SSC5D/ANGPTL3/MMP3/CD248/CDH2/MMP7/ASPN/VWC2/THBS4/OPTC/PRG2/OGN/CTHRC1/COL6A3/CTSC/CTSL/DPT/FBLN2/MFAP4/NID2/LGALS1/CTSD/ADAMTS4/MMP1/CTSH/LGALS9/TFPI2/MYOC/SPON2/TIMP1/COLEC12/VIT/ADAMTSL2/VEGFA/CCN3/F3/PHOSPHO1/MDK/TGFB1/EGFLAM/AGRN/CSPG4/DCN/VWA1/TINAGL1/EGFL7/COL4A4/CCDC80/PI3/MFAP5/ANGPTL4/ACHE/SMOC2/HSP90B1/FGL1/VCAN/FGA/MMP10/LRIG3/ADAMTS1/TGFBR3/ANXA5/IGFBP7/TIMP2/HRG/RARRES2/CTSZ/TNC/HSPG2/PRELP/PSAP/EDIL3/MATN2/CLEC14A/CSTB/SPARCL1/NCAM1/CCN4/C1QA/MUC2/ITIH5/CXCL12/COL24A1/POSTN/BCAM/COL4A1/COL28A1/SOST/PTPRZ1/KAZALD1/PDGFB/MST1/COL18A1/FN1/AMBP/BGN/ADAMTS15/ACAN/FBN2
GOMF_STRUCTURAL_MOLECULE_ACTIVITY	0.4657	1.9946	0.0000	0.0002	NEFL/GFAP/CHI3L1/CRYBB2/ELN/CCN1/LTBP2/VWF/MYBPC1/SRPX/MYL3/GP2/CILP/SPON1/ACTN2/PCOLCE/HSPB6/EFEMP1/BGLAP/HMOX1/KRT14/COMP/MRPL24/CSRP3/ASPN/MYOM1/OPTC/PRG2/OGN/CTHRC1/COL6A3/CLTA/DPT/FBLN2/MFAP4/NID2/MYLPF
GOCC_COLLAGEN_CONTAINING_EXTRACELLULAR_MATRIX	0.4365	1.9769	0.0000	0.0000	ELN/TGM2/CCN1/LTBP2/GDF15/VWF/TIMP3/CTSB/SRPX/PLAT/SMOC1/CILP/SPON1/SFRP1/PCOLCE/ANGPTL2/EFEMP1/MBL2/LEFTY2/FGFBP3/COMP/SSC5D/ANGPTL3/CDH2/ASPN/VWC2/THBS4/PRG2/OGN/CTHRC1/COL6A3/CTSC/CTSL/DPT/FBLN2/MFAP4/NID2/LGALS1/CTSD/ADAMTS4/CTSH/LGALS9/MYOC/TIMP1/VIT/CCN3/F3/MDK/TGFB1/EGFLAM/AGRN/CSPG4/DCN/VWA1/TINAGL1/EGFL7/COL4A4/CCDC80/MFAP5/ANGPTL4/ACHE/SMOC2/HSP90B1/FGL1/VCAN/FGA/ADAMTS1/ANXA5/IGFBP7/TIMP2/HRG/RARRES2/CTSZ/TNC/HSPG2/PRELP/PSAP/EDIL3/MATN2/CLEC14A/CSTB/SPARCL1/NCAM1/C1QA/MUC2/ITIH5/CXCL12/COL24A1/POSTN/BCAM/COL4A1/COL28A1/SOST/PTPRZ1/KAZALD1/PDGFB/MST1/COL18A1/FN1/AMBP/BGN/ADAMTS15/ACAN/FBN2
GOBP_LYMPHOCYTE_CHEMOTAXIS	0.5886	1.9478	0.0004	0.0168	CCL18/CCL24/CCL27/CCL11/CCL7/CXCL13/CCL2/CCL23/CCL20/CXCL11/CCL8/CXCL16/CCL16/CCL19/CCL14/XCL1/CCL25/CCL3/CCL15/SLC12A2/CCL21/CX3CL1/CXCL10/CCL13/TNFSF14
GOBP_ADENYLATE_CYCLASE_MODULATING_G_PROTEIN_COUPLED_RECEPTOR_SIGNALING_PATHWAY	0.5513	1.9354	0.0004	0.0157	PTH/ADGRG1/CALCA/CHGA/TCP11/GLP1R/GPHA2/GIP/ADGRB3/GHRHR/ADM/GIPR/GRK5/ADGRD1/VIPR1/AKAP12/CXCL11/GPR101/CXCL9/GPR37/PALM/PSAP/ATP2B4/ADCYAP1R1/PDE4D/MARCO/CXCL10/ADGRE5
GOMF_CCR_CHEMOKINE_RECEPTOR_BINDING	0.6164	1.9124	0.0003	0.0132	CCL18/CCL24/CCL27/CCL11/CCL7/CXCL13/CCL2/CCL23/CCL20/CCL8/CCL16/CCL19/CCL14/XCL1/CCL25/CCL3/CCL15/CCL21/CX3CL1/CCL13
GOBP_ADENYLATE_CYCLASE_ACTIVATING_G_PROTEIN_COUPLED_RECEPTOR_SIGNALING_PATHWAY	0.5755	1.8815	0.0013	0.0375	PTH/ADGRG1/CALCA/CHGA/TCP11/GLP1R/GPHA2/GIP/ADGRB3/GHRHR/ADM/GIPR/ADGRD1/CXCL11/GPR101/CXCL9
GOBP_GLIAL_CELL_PROLIFERATION	0.6492	1.8757	0.0020	0.0488	GFAP/IL6/TREM2
GOMF_MHC_PROTEIN_COMPLEX_BINDING	0.7246	1.8674	0.0016	0.0419	HLA-DRA/KLRD1/LILRB2/CD74/ATP1B1/CD4/LILRB1/CD8A/KLRC1/B2M
GOBP_G_PROTEIN_COUPLED_RECEPTOR_SIGNALING_PATHWAY	0.4312	1.8630	0.0000	0.0003	NPPB/PTH/TGM2/ADGRG1/FSHB/PPY/CCL18/ACTN2/CALCA/CCL24/CNTN2/SAG/CCL11/CHGA/TCP11/GLP1R/GPHA2/CD3E/CCL7/CA2/GIP/ADGRB3/IL2/SORT1/GHRHR/SORCS2/PARD3/ADM/GIPR/GRK5/GPRC5C/ADGRV1/SLC39A14/AGRN/NPR1/ADGRD1/CCL2/CCL23/CCL20/VIPR1/AKAP12/NPY/CXCL11/GPR101/CCL8/CXCL9/CCL16/GRP/CCL19/CCL14/TAFA5/XCL1/GAST/

					CCL25/GPR37/PALM/CCL3/PDGFRB/CCL15/PSAP/F2R/ATP2B4/ADCYAP1R1/CXCL8/KLK14/GNGT1/RGS8/CCL21/CX3CL1/PDE4D/CXCL12/MARCO/CXCL10/ADGRE5
GOBP_RESPONSE_TO_ARSENIC_CONTAINING_SUBSTANCE	0.7530	1.8579	0.0021	0.0489	NEFL/HMOX1/TNFRSF11B/MAPK13
GOMF_HEPARIN_BINDING	0.4498	1.8286	0.0001	0.0061	LPA/ADGRG1/CCN1/LTBP2/SMOC1/AOC1/SFRP1/PCOLCE/LXN/CCN5/FGFBP3/VEGFB/COMP/ANGPTL3/RSPO1/CCL7/THBS4/PRG2/CXCL13/PTN/VEGFA/CCN3/ADA2/PGF/MDK/NRP2/CCL23/REG4/CXCL11/NELL1/CCL8/CCDC80/PTPRF/SMOC2/ADAMTS1/TGFBR3/LRPAP1/HRG/NRP1/CCL15/PRELP/CXCL8/LPL/CCN4/RSPO3/POSTN/CXCL10/SOST/FGF9/FGFBP1/PDCD5/FN1/FSTL1/ADAMTS15
GOBP_REGULATION_OF_SYSTEMIC_ARTERIAL_BLOOD_PRESSURE	0.5788	1.8211	0.0013	0.0390	NPPB/TNNI3/CALCA/ADAMTS16/NOS3
GOBP_BONE_REMODELING	0.5514	1.8114	0.0014	0.0398	PTH/FSHB/IL6/SFRP1/CALCA/BGLAP/TNFRSF11B/SPP1/CTHRC1/TNFRSF11A/PTN/EPHA2/SIGLEC15/ACP5/MDK/TGFB1
GOBP_REGULATION_OF_BLOOD_PRESSURE	0.5100	1.8015	0.0014	0.0398	NPPB/TNNI3/ACTA2/CALCA/ADAMTS16/NOS3/HMOX1/CHGA/GLP1R/NOS1
GOMF_G_PROTEIN_COUPLED_RECEPTOR_BINDING	0.4364	1.7790	0.0001	0.0076	MLN/PTH/PPY/CCL18/WNT9A/SFRP1/CALCA/CCL24/SAG/CCL27/CCL11/RSPO1/GPHA2/CCL7/GIP/CTHRC1/CXCL13/IL2/ADM/MYOC/ADA2/CXCL14/CCL2/CCL23/CCL20/C1QBP/NPY/CXCL11/CCL8/CXCL9/CXCL16/CCL16/CCL19/CCL14/TAFA5/XCL1/CCL25/PALM
GOBP_DETECTION_OF_STIMULUS	0.5069	1.7665	0.0016	0.0422	SYT1/SRPX/TREM2/CDHR2/CALCA/SAG/LXN/SSC5D/CDH2/LY96/HLA-A/NGFR/CSRP3/CST1/CRTAM/ADGRV1/PKD1/PGLYRP4
GOBP_TISSUE_REMODELING	0.4699	1.7620	0.0008	0.0262	PTH/FSHB/IL6/SFRP1/CALCA/NOS3/BGLAP/TNFRSF11B/DBH/SPP1/THBS4/CTHRC1/IL2/TNFRSF11A/DLL4/PTN/TIMP1/EPHA2/SIGLEC15/ACP5/MDK/TGFB1/CST3/CSPG4/LEP/LEPR/HRG/TP53/IL1A/F2R/CD38/TPP1/ACVRL1
GOBP_POSITIVE_REGULATION_OF_ERK1_AND_ERK2_CASCADE	0.4320	1.7612	0.0002	0.0086	CHI3L1/CXCL17/FGF21/ACTA2/TREM2/CCL18/CCL24/PLA2G2A/FGF19/HRAS/CCL11/DENND2B/CCL7/TNFRSF11A/LGALS9/CD74/TGFB1/CCL2/CCL23/HAVCR2/PDGFRA/CCL20/AKAP12/NPY/CCL8/CCL16/CCL19/FGA/CCL14/XCL1/CD4/CCL25/NTRK2/ROR1/CCL3/PDGFRB/IL1A/NRP1/CCL15/F2R
GOMF_SULFUR_COMPOUND_BINDING	0.4204	1.7602	0.0003	0.0132	LPA/ADGRG1/CCN1/LTBP2/SMOC1/AOC1/SFRP1/PCOLCE/LXN/CCN5/FGFBP3/VEGFB/COMP/ANGPTL3/RSPO1/GSTP1/CCL7/THBS4/PRG2/CXCL13/PTN/SCP2/VEGFA/CCN3/ADA2/PGF/MDK/NRP2/AGRN/CCL23/TPMT/REG4/CXCL11/NELL1/CCL8/CCDC80/PTPRF/SMOC2/ADAMTS1/TGFBR3/LRP1/LRPAP1/HRG/NRP1/CCL15/PRELP/CBS/CXCL8/LPL/CCN4/RSPO3/POSTN/CXCL10/SOST/FGF9/FGFBP1/PDCD5/FN1/FSTL1/ADAMTS15
GOMF_METALLOPEPTIDASE_ACTIVITY	0.4591	1.7413	0.0007	0.0249	AGBL2/CPA1/CPB1/PRSS2/ADAMTS16/MEP1A/XPNPEP2/MEP1B/MMP3/MMP7/LTA4H/PAPPA/ADAM12/ADAMTS4/MMP1/ADAMTSL2/CPA2
GOMF_GROWTH_FACTOR_BINDING	0.4437	1.7409	0.0006	0.0243	IGFBP2/CCN1/LTBP2/CCN5/FGFBP3/NGFR/IGFBP4/KLB/CXCL13/SORT1/GHRHR/EPHA2/FGFBP2/CCN3/IGFBPL1/NRP2/PDGFRA/TGFBR2/CRIM1/S100A13/NTRK2/FURIN/TGFBR3/LRP2/IL2RB/ESM1/IGFBP7/PDGFRB/IGFBP6/NRP1/ACVRL1/OSMR/IGF1R/CCN4/LIFR/COL4A1/FGFBP1/KAZALD1/PDGFB/FLT1/NTRK3/LTBP3/ERBB2
GOMF_GROWTH_FACTOR_ACTIVITY	0.4224	1.7253	0.0004	0.0185	FGF21/IL11/IL6/GDF15/CNTF/EFEMP1/FGF19/LEFTY2/INHBB/FGF5/REG1A/VEGFB/TFF1/ANGPTL3/THBS4/GDNF/OGN/IL2/VEGFD/HGF/PTN/TIMP1/VEGFA/CCN3/DKK1/CSPG5/ADA2/PGF/MDK/TGFB1
GOCC_CELL_SURFACE	0.3544	1.7092	0.0000	0.0002	ALPP/CTLA4/CTSB/HAVCR1/SRPX/PLAT/GP2/DUOX2/SFRP1/CD27/CNTN2/CD14/MSLN/HLA-DRA/FOLR3/MBL2/LAMP3/IL5RA/SLAMF7/ANGPTL3/CD248/ITGBL1/CDH2/HLA-A/NGFR/KLRD1/NOTCH3/IL13RA2/HMMR/CD3E/CNTNAP2/MXRA8/GPIHBP1/CEACAM5/IL4R/GFRAL/TNFRSF10B/GFRA1/TFPI/IL15RA/CD79B/MSR1/LAYN/SORT1/TNFRSF11A/GHRHR/CLEC5A/MOG/TNFRSF10A/FAS/EPHA2/LY9/VEGFA/ADGRV1/LILRB2/F3/CD74/CSPG5/LILRB4/CD302/TGFB1/TSPAN8/SLAMF8/SCARA5/TNFRSF1A/CSPG4/VAMP5/BTN3A2/PKD1/HAVCR2/ITGB5/PDGFRA/EGFL7/PLA2G1B/C1QBP/NLGN2/CFC1/CXCL9/LILRA5/FCRL5/ACHE/TGFBR2/PCSK9/MCAM/FGA/CLMP/NT5E/CLSTN2/CD59/CD4/NECTIN2/GPR37/LILRB1/CD55/FURIN/ROR1/TGFBR3/PDIA3/CDH17/LRP2/CD8A/IL2RB/FCRL6/LEPR/ANXA5/LRPAP1/HRG/KLRC1/B2M/PDGFRB/DCBLD2/BTN2A1/IL1A/DSG2/MRC1/ITGA5/F2R/THBD/IGLC2/CD38/CLEC14A/LY75/ADCYAP1R1/JAM2/ITGB6/CD276/SIRPA/FOLR1/NCAM1/ACVRL1/PLAUR/OSMR/LPL/LIFR/SIRPB1/SDC1/CLSTN3/CD83/CX3CL1/FOLR2/CXCL12/CXCL10/BCAM/PAM/HSPA2/TNFRSF10C/SLAMF6/CD207/BTN1A1/THY1/IL15/CSF3R/CD46/FGFBP1/PLXNB2/TNFRSF14/PDGFB/LBP/ACE2/AMBP/BGN/ADAMTS15/PLXNB3/ERP44/PLAU/ULBP2/IGSF3/FCGR2B/TLR2/AREG/LAG3/CLEC7A/ICOSLG/ADAM9/LDLR/ADAMTS13/ITGAV/ATP1B2/HEG1/FCRL2/IL17A/PDCD1LG2/NPTN/ITGB2/FGFR2/CD40/CD2/FCER1A/SEMA7A/APOH/IL6R/IL6ST/RTBDN/CD274/GP1BA/PTPRK/GFRA3/CLEC6A/ITGAM/IL1R1/LY6D/ICAM1/CD5L/CLEC10A/LGALS3/SELE/ISLR2/VASN/ENO2/CDH5/CSF2RA/NEO1/EPO/CD93/ASGR1/CD200/AOC3/SLAMF1/SLITRK6/APP/VCAM1/FCN1/CD36/CD34/CD3D/IGF2R/CD63/DPP4
GOMF_SIGNALING_RECEPTOR_REGULATOR_ACTIVITY	0.3675	1.7026	0.0000	0.0008	MLN/NPPB/PTH/FGF21/IL11/FSHB/IL6/GDF15/PPY/IFNL2/CCL18/CNTF/WNT9A/CALCA/EFEMP1/INSL4/CCL24/FGF19/CCL27/LEFTY2/INHBB/CCL11/TNFRSF11B/IL31/FGF5/REG1A/VEGFB/TFF1/ANGPTL3/GPHA2/DKK3/SPP1/CCL7/GIP/THBS4/SCGB3A1/GDNF/OGN/CXCL13/IL2/VEGFD/HGF/ADM/PTN/TIMP1/SEMA4C/VEGFA/CCN3/STC1/DKK1/CSPG5/ADA2/LILRB4/PGF/MDK/TGFB1/TNFSF13/CXCL14/CCL2/CCL23/IFNL1/CCL20/NPY/CXCL11/CCL8/CXCL9/CXCL16/FAM3C/CCL16/GRP/PCSK9/CCL19/CCL14/TAFA5/XCL1/FAM3D/GAST/CCL25/KITLG/INHBC
GOMF_MOLECULAR_TRANSDUCER_ACTIVITY	0.3566	1.6824	0.0000	0.0008	KIR2DL3/ADGRG1/EDA2R/EPHA10/TREM2/EFEMP1/CD27/IL1RL2/GABRA4/CD14/REG1B/HLA-DRA/FOLR3/TNFRSF11B/REG1A/IL22RA1/GLP1R/IL5RA/LY96/REG3A/NGFR/KLRD1/NOTCH3/IL13RA2/CD3E/REG3G/IL4R/GFRAL/TNFRSF10B/GFRA1/IL17RB/ADGRB3/M6PR/IL15RA/SIGLEC7/CD79B/SORT1/TNFRSF11A/GHRHR/SORCS2/CTSH/CD300E/GIPR/TNFRSF10A/FAS/COLEC12/EPHA2/GPRC5C/IL1RAP/ADGRV1/LILRB2/F3/CD74/SCARB2/CD302/SLAMF8/NRP2/TNFRSF1A/CSPG4/NPR1/ADGRD1/PKD1/PGLYRP4/GUCY2C/ITGB5/REG4/PDGFRA/VIPR1/NLGN2/NPY/GPR101/CD300LG/LILRA5/FCRL5/TNFRSF17/PTPRF/TGFBR2/CD300LF/CRIM1/CD4/TNFRSF6B/NECTIN2/GPR37/NTRK2/LILRB1/ROR1/TGFBR3/CD8A/IL18R1/IL2RB/FCRL6/LEPR/LRP1/CLEC1A/KLRC1/TNFRSF13B/PGLYRP2/PDGFRB/EFNA4/NRP1/MRC1/IFNGR1/F2R/PIGR/THBD/LY75/ADCYAP1R1/ITGB6/FOLR1/ACVRL1/SIGLEC8/PLAUR/OSMR/IGF1R/LIFR/TNFRSF1B/ERBB4/EPHA1/FOLR2/MARCO/ADGRE5/BCAM/TNFRSF10C/IL20RA/LILRA2/BTN1A1/CD300C/CSF3R/CD46/PTPRZ1/RGMB/PLXNB2/TNFRSF14

GOBP_EXTERNAL_ENCAPSULATING_STRUCTURE_ORGANIZATION	0.4012	1.6811	0.0008	0.0277	GFAP/KLK4/ELN/CCN1/IL6/SMOC1/PRSS2/ADAMTS16/TNFRSF11B/COMP/MMP3/MMP7/OPTC/SERPINB5/DPT/FBLN2/MFAP4/NID2/ADAMTS4/MMP1/VIT/ADAMTSL2/TGFB1/EGFLAM/TNFRSF1A/CST3/VWA1/PDGFRA/COL4A4/CCDC80/SMOC2/MMP10/FURIN/ADAMTS1
GOMF_CYTOKINE_ACTIVITY	0.3886	1.6509	0.0006	0.0240	IL11/IL6/GDF15/IFNL2/CCL18/CNTF/WNT9A/CCL24/CCL27/LEFTY2/INHBB/CCL11/TNFRSF11B/IL31/SPP1/CCL7/SCGB3A1/CXCL13/IL2/TIMP1/VEGFA/TGFB1/TNFSF13/CXCL14/CCL2/CCL23/IFNL1/CCL20/CXCL11/CCL8/CXCL9/CXCL16/FAM3C/CCL16/CCL19/CCL14/TAFA5/XCL1/FAM3D/CCL25/KITLG/INHBC/CCL3/IL1A/CCL15/IL36A/CXCL8/THPO/CCL21/CX3CL1/FAM3B/CXCL12/CD70/CXCL10/GDF2/IL15/CCL13/TSLP/TNFSF14/IL33/TNFSF10/IL17D/AREG/TNFSF12
GOMF_ENDOPEPTIDASE_ACTIVITY	0.3784	1.6112	0.0009	0.0307	LPA/KLK4/CTSB/PLAT/CELA2A/SFRP1/PRSS2/CTRB1/ADAMTS16/MEP1A/ESPL1/MEP1B/MMP3/MMP7/CTRL/PAPPA/CTSC/CTSL/ADAM12/CTSD/ADAMTS4/MMP1/HGF/CTSH/ADAMTSL2/CTSE/F3/CELA3A/GZMH/TPSD1/PCSK9/TMPRSS5/MMP10/FURIN/ADAMTS1/PDIA3/CASP4/CFD/CTRC/CTSZ/DDI2/KEL/USP47/TPP1/CAPN3/HGFAC/KLK14/MST1/ACE2/ATG4A/PRSS8/ADAMTS15/KLK1/PLAU/MASP1/CTSF/ADAM9/CASP8/ADAMTS13/KLK3/CTSO/F7/KLK6/USP28/C2
GOMF_PEPTIDASE_ACTIVITY	0.3455	1.5417	0.0014	0.0398	LPA/KLK4/TGM2/AGBL2/CTSB/PLAT/CPA1/CELA2A/CPB1/SFRP1/PRSS2/CTRB1/ADAMTS16/MEP1A/XPNPEP2/ESPL1/MEP1B/MMP3/MMP7/LTA4H/CTRL/GGT1/PAPPA/CTSC/CTSL/DPP6/ADAM12/CTSD/ADAMTS4/MMP1/HGF/CTSH/ADAMTSL2/CTSE/CPA2/F3/DPP7/CELA3A/GZMH/DPP10/TINAGL1/CPXM2/TPSD1/PCSK9/TMPRSS5/MMP10/FURIN/ADAMTS1/PDIA3/CASP4/CFD/CTRC/CTSZ/DPEP1/DDI2/KEL/USP47/TPP1/PRCP/LAP3/CAPN3/HGFAC/KLK14
GOCC_INTRINSIC_COMPONENT_OF_PLASMA_MEMBRANE	0.3139	1.5225	0.0001	0.0050	TGM2/KIR2DL3/ADGRG1/EDA2R/IL6/CTLA4/EPHA10/TREM2/GP2/DUOX2/CDHR2/CD82/CD27/IL1RL2/CNTN2/GABRA4/CDH4/VSIG2/CD14/HLA-DRA/FOLR3/KIR3DL2/MEP1A/IL22RA1/GLP1R/C1QTNF1/MEP1B/ITGBL1/CDH2/LY96/HLA-A/SLC16A1/NGFR/KLRD1/CD3E/VWC2/CNTNAP2/GPIHBP1/CEACAM5/IL4R/IL17RB/ADGRB3/M6PR/SIGLEC7/CD79B/SCN2A/DPP6/MSR1/SORCS2/CLEC5A/UPK3A/SLC39A5/BSND/EPHA2/GPRC5C/SEMA4C/IL1RAP/LILRB2/F3/CSPG5/MDGA1/LILRB4/SLC39A14/TSPAN8/SCARA5/NRP2/TNFRSF1A/KCNC4/CSPG4/VAMP5/XG/ADGRD1/PKD1/DPP10/SLC4A1/TSPAN1/ITGB5/IFNL1/PDGFRA/FCAR/VIPR1/NLGN2/GPR101/FCRL5/PTPRF/ATP1B1/TGFBR2/PCDH9/CLSTN2/CD59/CD4/GPR37/NTRK2/PALM/ROR1/TGFBR3/RYR1/CD8A/IL18R1/ICAM5/TRPV3/IL2RB/FCRL6/LRP1/CLEC1A/KIRREL1/SCN3B/CEMIP2/KLRC1/TNFRSF13B/PDGFRB/DCBLD2/BTN2A1/EFNA4/NRP1/KIR2DS4/MRC1/VWC2L/IFNGR1/ITGA5/F2R/PIGR/THBD/LY75/ATP2B4/ADCYAP1R1/ICAM3/SEMA3F/JAM2/ITGB6/SLC12A2/SIRPA/FOLR1/ACVRL1/C9/NFASC/PLAUR/OSMR/IGF1R/LIFR/SIRPB1/SDC1/CLSTN3/CD83/ERBB4/EPHA1/ICAM2/FOLR2/MARCO/CD70/ADGRE5/BCAM/IL20RA/LILRA2/BTN1A1/FUT1/THY1/CD300C/SCN3A/CSF3R/CD46/PILRB/PTPRZ1/EPGN/RGMB/PLXNB2/ATP1B3/CD99/PCDHB15/ENPP7/SCN4B/EVI2B/MILR1/FLT1/ICAM4/DDR1/PLXNB3/SCN2B/MERTK/NTRK3/ULBP2/EREG/TNFSF10/TNFRSF21/FCGR2B/CDHR5/TLR2/TNFSF12/CD160/ERBB2/GGT5/ADAM9/LDLR/ITGAV/ATP1B2/BSG/JAM3/FCRL2/NPTN/ITGB2/FGFR2/C8B/CD40/SEMA6C/IFNLR1/GPNMB/CD2
GOBP_CYTOSKELETON_ORGANIZATION	-0.2846	-1.4572	0.0006	0.0226	NUDC/ARHGAP25/CEP350/FTCD/DYNC1H1/MDM1/ABL1/CNP/ERBIN/PPP1R12A/CHMP6/SLC9A3R1/DNAJB6/ANXA1/PAK4/FKBP4/EPPK1/CEP85/IQGAP2/FZD10/MZT1/HCLS1/SDC4/PPP1R9B/EHBP1/MYOM2/PDLIM7/TNXB/TF/BAIAP2/RICTOR/PRC1/SSH3/CEP20/CHEK2/ATF2/KRT17/PXN/WASF3/AMOT/CETN3/PPM1F/SLK/VASP/ARFIP1/CCL26/NEXN/CEP152/BAG4/CDK1/NPHS1/CLIP2/DLGAP5/TWF2/NEDD9/VPS4B/SOX9/CSF3/DBNL/BRSK2/SPRY2/NAA80/FMNL1/TXNDC9/GIT1/LATS1/BCR/CENPJ/DMD/WAS/PHACTR2/BST1/GMFG/AXIN1/WASHC3/OFD1/ARF6/KIT/CXCL1/PSTPIP2/ADRA2A/PKD2/CORO1A/MICALL2/RHOC/FGD3/INPPL1/MYCBP2/TPR/MTSS1/LMOD2/TPPP2/PTPN1/EPB41L5/MYH9/CDC42BPB/DCTN6/PRKG1/NUBP1/TSC1/CSNK1D/NEK7/SRC/NPM1/SIRT1/PDLIM5/DYNLT1/SDCCAG8/OPHN1/DAAM1/ARHGEF10/TACC3/WASL/SMTN/CAMSAP1/MAPRE3/ODAM/AFAP1/AIF1
GOMF_PROTEIN_DIMERIZATION_ACTIVITY	-0.2906	-1.4589	0.0011	0.0336	NUDT16/HPGDS/GSTM4/CRYM/COMMD1/SCLY/ID4/CHEK2/ATF2/RPE/CEACAM6/SLC51B/ZHX2/STAT5B/SLK/GHR/S100A11/STC2/NECTIN1/CALCOCO2/SHMT1/GBP4/VPS4B/IRAK1/CASQ2/BNIP3L/SNX2/CEBPB/HHEX/AXIN1/MAX/TCOF1/RILPL2/RABEP1/H2AP/KIT/LRRFIP1/ADRA2A/AIFM1/ERP29/TYRP1/TERF1/PKD2/CORO1A/ARNT/TARBP2/UPB1/CACYBP/FMR1/BCL2L1/TPR/MYO9B/UXS1/MYH9/IKBKG/GBP1/THAP12/MSTN/RAB11FIP3/GRHPR/NPM1/RBPMS2/KIF20B/BAX/RBPMS/CDA/APPL2/CR2/PAFAH1B3/MAP3K5
GOMF_PROTEIN_HOMODIMERIZATION_ACTIVITY	-0.3047	-1.5074	0.0014	0.0398	NUDT16/HPGDS/GSTM4/CRYM/COMMD1/SCLY/CHEK2/ATF2/RPE/ZHX2/STAT5B/SLK/GHR/S100A11/STC2/NECTIN1/CALCOCO2/SHMT1/GBP4/VPS4B/IRAK1/CASQ2/BNIP3L/SNX2/CEBPB/HHEX/AXIN1/RABEP1/KIT/LRRFIP1/ADRA2A/ERP29/TYRP1/TERF1/PKD2/CORO1A/ARNT/TARBP2/UPB1/CACYBP/FMR1/BCL2L1/TPR/MYO9B/UXS1/MYH9/IKBKG/GBP1/MSTN/RAB11FIP3/GRHPR/NPM1/RBPMS2/KIF20B/BAX/RBPMS/CDA/APPL2/CR2/PAFAH1B3/MAP3K5
GOBP_REGULATION_OF_ORGANELLE_ORGANIZATION	-0.2991	-1.5097	0.0003	0.0127	BAIAP2/HDAC8/RICTOR/RUVBL1/SSH3/PXN/WASF3/BECN1/PPM1F/VASP/EGF/CALCOCO2/ARFIP1/CCL26/NEXN/BAG4/NPHS1/LIF/DLGAP5/STAM/TWF2/VPS4B/ENTR1/CSF3/DBNL/BNIP3L/NAA80/NMT1/RNF5/GIT1/LATS1/CENPJ/WAS/BST1/GMFG/MKI67/ARF6/BTC/TERF1/SMPD3/DDHD2/CORO1A/STAT2/RHOC/PARP1/STX5/EVI5/MYCBP2/BCL2L1/TPR/MTSS1/LMOD2/EHD3/FEN1/NBN/AKT2/NUBP1/TSC1/NEK7/SRC/NPM1/PIKFYVE/TBC1D5/KIF20B/SDCCAG8/BAX/SEPTIN7/ARHGEF10/TACC3/YY1/MAVS/WASL/CAMSAP1/DOC2B/MAPRE3/ODAM/AFAP1
GOBP_PROTEIN_LOCALIZATION_TO_ORGANELLE	-0.3228	-1.5175	0.0019	0.0470	RAB10/EGF/LZTFL1/IL10RA/TRIM40/BAG4/CDK1/LIF/STAM/SRP14/ENTR1/SOX9/GGA1/MORC3/BNIP3L/DLG4/NMT1/LATS1/HHEX/RABEP1/ARF6/AIFM1/TERF1/MICALL2/ING1/PARP1/RAP1A/TPR/TOR1AIP1/GBP1/CSNK1D/RAB11FIP3/SRC/PIKFYVE/KIF20B/CAMLG/BAX/HDGF/MAVS/APPL2/MAPRE3/EIF2AK3
GOCC_SUPRAMOLECULAR_COMPLEX	-0.3083	-1.5388	0.0009	0.0294	SEPTIN9/DYNC1H1/MDM1/COL2A1/PPP1R12A/MYBPC2/CHMP6/DNAJB6/ANXA1/ITGB1BP2/FKBP4/LMNB1/EPPK1/BLNK/HCLS1/SDC4/EHBP1/CIRBP/TBCC/MYOM2/PDLIM7/EIF4E/TBCA/BAIAP2/PRC1/PPP1CC/KRT17/AMOT/GHR/SUGT1/TBCB/EIF4G1/DNM1/PSMC3/NEXN/CSDE1/STAU1/CDK1/CLIP2/TWF2/YES1/CASQ2/XIAP/DBNL/SPRY2/TP53BP1/CENPJ/DMD/TPT1/WAS/LARP1/DYNLT3/DCDC2C/PSTPIP2/CORO1A/CASC3/LDLRAP1/FKBP1B/GIGYF2/FMR1/TPR/MYO9B/LMOD2/TPPP2/TOP1/MYH9/DCTN6/CEP170/SERPINB1/TSC1/CSNK1D/NEK7/PPP2R5A/SRC/SNRPB2/PDLIM5/DYNLT1/KIF20B/SEPTIN7/RBPMS/TRIM25/TDRKH/REEP4/WASL/SMTN/PPP1R12B/CAMSAP1/RASSF2/MAPRE3/ODAM/MYL6B/AIF1/SLC1A4
GOMF_PROTEIN_DOMAIN_SPECIFIC_BINDING	-0.3240	-1.5402	0.0016	0.0424	LAMP2/CRX/TBL1X/TADA3/ABL1/IL1B/SLC9A3R1/SH3BGRL2/ITGB1BP2/CASP2/BLNK/HCLS1/ZBTB16/IST1/RNF41/XRCC4/BAIAP2/PPP1CC/ATF2/GHR/VASP/ICA1/CASP1/PAG1/ARFIP1/ARHGAP1/DBNL/DLG4/INPP5D/CENPJ/WAS/AXIN1/MITD1/CASP10/PTPN6/RABEP1/KIT/SKAP1/PDZK1/TERF1/PKD2/CACYBP/INPPL1/LBR/CASP9/BCL2L1/PRKAR2A/GATA3/EPB41L5/TOP1/MYH9/NFE2/IKBKG/DDC/RAB27B/SRC/SIRT1/KIF20B/BAX/CALCOCO1/SYNGAP1/MAVS/AIDA/MAP3K5/AFAP1
GOBP_POSITIVE_REGULATION_OF_CELLULAR_BIOSYNTHETIC_PROCESS	-0.2975	-1.5478	0.0001	0.0045	ID4/RUVBL1/FOS/CHEK2/ATF2/KRT17/TLR3/BIRC2/PCBD1/SLC51B/SNX5/STAT5B/EGF/EIF4G1/PF4/GCG/PRKD2/WWP2/PSMC3/CSDE1/TRIM24/FOXJ3/CDK1/LIF/YES1/ITGA2/MYDGF/JUN/SOX9/CSF3/MMP8/TP53BP1/TBR1/CENPJ/PRL/CEBPB/HHEX/NFKB1/WAS/AXIN1/LARP1/MAX/NFATC3/TP73/GLYR1/MARS1/SKAP1/PCBP2/TYRP1/KDM3A/MTDH/PKD2/STAT2/APEX1/ARNT/

					TARBP2/FOXO1/ING1/PARP1/TNFSF11/FMR1/BACH1/GATA3/IL4/NFE2/ASS1/IKBKG/NFATC1/AKT2/MSTN/NEK7/TNIP1/TXK/EDF1/NFYA/SRC/NPM1/SIRT1/RBPMS/TRIM25/CALCOCO1/FGFR4/HDGF/YY1/MAVS/WASL/RET/MAPRE3/EIF2AK3/MAP3K5/FOXO3/NFAT5/AIF1/ZP3
GOCC_CATALYTIC_COMPLEX	-0.3312	-1.5956	0.0007	0.0258	TBL1X/TADA3/DYNC1H1/PPP1R12A/CCND2/PSMA1/CDC27/CBX2/GMPR2/CASP2/CHM/BCL7A/ZCCHC8/PPP1R9B/RALY/HDAC8/COMMD1/RFC4/PPP1CC/SCLY/RUVBL1/ATF2/BECN1/SLA2/SUGT1/RNF168/WWP2/PSMC3/TRIM40/CDK1/YJU2/ATG16L1/CWC15/BCL7B/GNAS/TRAF2/MAX/CASP10/DYNLT3/DCUN1D1/HDAC9/FARSA/KDM3A/NDUFB7/CASC3/TARBP2/CACYBP/MORF4L1/CASP9/CDKN2D/PRKAR2A/TPR/UXS1/DECR1/IKBKG/PRKAG3/BRAP/DDA1/GRHPR/PPP2R5A/SNRPB2/DYNLT1/YY1/SART1/PAFAH1B3/MAP3K5/NCF2
GOBP_MICROTUBULE_BASED_PROCESS	-0.3438	-1.5970	0.0015	0.0398	NUDC/CEP350/DYNC1H1/MDM1/ABL1/CNP/PPP1R12A/CHMP6/SLC9A3R1/FKBP4/CEP85/MZT1/PRC1/CEP20/CHEK2/CETN3/SLK/CELSR2/CEP152/CDK1/CLIP2/DLGAP5/VPS4B/BRSK2/SPRY2/TXNDC9/GIT1/CENPJ/AXIN1/DYNLT3/OFD1/PKD2/SORD/TPR/BLOC1S2/TPPP2/MYH9/DCTN6/NUBP1/CSNK1D/RAB27B/NEK7/NPM1/SIRT1/DYNLT1/KIF20B/SDCCAG8/ARHGEF10/TACC3/CAMSAP1/RPGR/MAPRE3
GOBP_POSITIVE_REGULATION_OF_RNA_METABOLIC_PROCESS	-0.3133	-1.5972	0.0001	0.0041	ID4/RUVBL1/FOS/CHEK2/ATF2/TLR3/BIRC2/PCBD1/SNX5/STAT5B/EGF/PF4/PRKD2/WWP2/PSMC3/CSDE1/TRIM24/FOXJ3/LIF/YES1/MYDGF/JUN/SOX9/CSF3/TP53BP1/TBR1/CENPJ/PRL/CEBPB/HHEX/NFKB1/WAS/AXIN1/MAX/NFATC3/TP73/GLYR1/MARS1/SKAP1/PCBP2/KDM3A/MTDH/PKD2/STAT2/APEX1/ARNT/FOXO1/ING1/PARP1/TNFSF11/GIGYF2/BACH1/GATA3/IL4/NFE2/IKBKG/NFATC1/MSTN/TNIP1/TXK/EDF1/NFYA/SRC/NPM1/SIRT1/RBPMS/TRIM25/CALCOCO1/HDGF/YY1/MAVS/WASL/RET/MAPRE3/EIF2AK3/MAP3K5/FOXO3/NFAT5/ZP3
GOBP_POSITIVE_REGULATION_OF_NUCLEOBASE_CONTAINING_COMPOUND_METABOLIC_PROCESS	-0.3129	-1.6069	0.0000	0.0019	ID4/RUVBL1/FOS/CHEK2/ATF2/TLR3/BIRC2/PCBD1/SNX5/STAT5B/EGF/RNF168/PF4/PRKD2/WWP2/PSMC3/CSDE1/TRIM24/FOXJ3/LIF/YES1/MYDGF/JUN/SOX9/CSF3/TP53BP1/TBR1/CENPJ/PRL/CEBPB/HHEX/NFKB1/WAS/AXIN1/ANXA3/MAX/NFATC3/TP73/GLYR1/MARS1/SKAP1/PCBP2/KDM3A/MTDH/PKD2/STAT2/APEX1/ARNT/FOXO1/ING1/PARP1/TNFSF11/MORF4L1/GIGYF2/BACH1/GATA3/HDGFL2/IL4/NFE2/NBN/IKBKG/NFATC1/MSTN/NEK7/TNIP1/TXK/EDF1/NFYA/SRC/NPM1/SIRT1/MGMT/BAX/RBPMS/TRIM25/CALCOCO1/FGFR4/HDGF/YY1/MAVS/WASL/RET/MAPRE3/EIF2AK3/MAP3K5/FOXO3/NFAT5/ZP3
GOBP_MICROTUBULE_CYTOSKELETON_ORGANIZATION	-0.3726	-1.6270	0.0019	0.0465	NUDC/CEP350/DYNC1H1/MDM1/ABL1/CNP/PPP1R12A/CHMP6/FKBP4/CEP85/MZT1/PRC1/CEP20/CHEK2/CETN3/SLK/CEP152/CDK1/CLIP2/DLGAP5/VPS4B/BRSK2/SPRY2/TXNDC9/GIT1/CENPJ/AXIN1/OFD1/PKD2/TPR/TPPP2/MYH9/DCTN6/NUBP1/CSNK1D/NEK7/NPM1/SIRT1/DYNLT1/SDCCAG8/ARHGEF10/TACC3/CAMSAP1/MAPRE3
GOCC_ACTIN_CYTOSKELETON	-0.3563	-1.6284	0.0012	0.0369	SEPTIN9/ABL1/PPP1R12A/SLC9A3R1/ANXA1/DDX58/ZNF174/FYB1/IQGAP2/HCLS1/PPP1R9B/EHBP1/PDLIM7/BAIAP2/PXN/AMOT/MTSS2/VASP/LSP1/NEXN/TWF2/YES1/DBNL/SPRY2/DMD/WAS/PSTPIP2/CORO1A/MICALL2/MTSS1/MYO9B/LMOD2/CGN/MYH9/CDC42BPB/GBP1/DCTN6/TSC1/BIN2/SRC/PDLIM5/SEPTIN7/FGR/OPHN1/DAAM1/WASL/SMTN/AFAP1/MYL6B/AIF1
GOBP_PROTEIN_MODIFICATION_BY_SMALL_PROTEIN_CONJUGATION_OR_REMOVAL	-0.3714	-1.6468	0.0021	0.0489	CDC26/RNF43/CHP1/OTUD7B/TRIM5/TADA3/ABL1/CDC27/ZBTB16/RNF41/HDAC8/COMMD1/BIRC2/TRAF3/RNF168/WWP2/TRIM40/USP25/TRIM24/CDK1/OTUD6B/NUB1/XIAP/SPRY2/RNF5/TRAF2/AXIN1/DCUN1D1/NFX1/ARNT/UBE2Z/MYCBP2/BRAP/DDA1/TNIP1/SIRT1/CAMLG/TRIM25/MINDY1
GOBP_CELLULAR_MACROMOLECULE_BIOSYNTHETIC_PROCESS	-0.3312	-1.6487	0.0001	0.0078	NPPC/S100A11/SRPK2/EGF/EIF4G1/PRKD2/CSDE1/SHMT1/CDK1/PAIP2B/ITGA2/DUT/ATG16L1/NMT1/LARP1/TCOF1/MRPL58/GTPBP2/MARS1/EIF1AX/ADRA2A/FARSA/TERF1/SMPD3/CASC3/OGA/TARBP2/EXTL1/GIGYF2/CDKN2D/FMR1/PPM1B/EIF2S2/TPR/FEN1/GYS1/EEF1D/TOP1/TK1/NBN/PMM2/PRKAG3/AKT2/TSC1/DTD1/NEK7/TNIP1/SRC/NPM1/SIRT1/FGFR4/YY1/EIF2AK2/BANK1/EIF4G3/EIF2AK3/FOXO3
GOBP_ENDOMEMBRANE_SYSTEM_ORGANIZATION	-0.3730	-1.6498	0.0016	0.0418	MTSS2/TJAP1/RAB10/GORASP2/DNM1/CDK1/STAM/SEC31A/VPS4B/LYSMD3/SNAP23/STX5/STX6/STX16/MTSS1/BLOC1S2/EHD3/FABP9/SERPINE2/MYH9/TOR1AIP1/SYTL4/AKT2/ESYT2/CSNK1D/BIN2/CAMLG/REEP4/WASL/EIF2AK3
GOBP_VESICLE_ORGANIZATION	-0.4107	-1.6841	0.0015	0.0415	VAV3/DNM1/STAM/SEC31A/VPS4B/DLG4/SNAP23/VAMP8/TYRP1/CORO1A/STX8/STX7/STX5/STX6/STX16/BLOC1S2/FABP9/SERPINE2/AKT2/CSNK1D/PIKFYVE/WASL/IRAG2/DOC2B
GOMF_KINASE_BINDING	-0.3410	-1.6868	0.0001	0.0050	HCLS1/SDC4/PPP1R9B/IL31RA/MYOM2/RNF41/IRAK4/RICTOR/PRC1/PPP1CC/SPRED2/CHEK2/ATF2/BECN1/GHR/TRAF3/CASP1/DNM1/CRKL/PRKD2/CEP152/TWF2/PTPRR/NEDD9/IRAK1/SOX9/XIAP/BRSK2/SPRY2/DLG4/TBR1/LATS1/CENPJ/CEBPB/TRAF2/WAS/AXIN1/TAX1BP1/PTPN6/TP73/SV2A/IL12RB2/SKAP1/ADRA2A/HDAC9/RHOC/SH2B3/PARP1/CASP9/MAPKAPK2/CDKN2D/BCL2L1/TCL1A/GP6/PRKAR2A/TPR/MAP2K6/GYS1/PTPN1/ITGAX/NFATC1/PRKAG3/PFKFB2/TNIP1/PPP2R5A/SRC/NPM1/SIRT1/PDLIM5/FGR/MAVS/TCL1B/PPP1R12B/BANK1/MAPRE3/MAP3K5/FOXO3
GOBP_POSITIVE_REGULATION_OF_CELL_CYCLE	-0.4022	-1.6953	0.0010	0.0327	DYNC1H1/ABL1/IL1B/CCND2/ANXA1/CDC27/NUDT16/EIF4E/CHEK2/BECN1/STAT5B/SRPK2/EGF/EIF4G1/CDK1/DLGAP5/VPS4B/CRNN/CENPJ/DYNLT3/BTC/SMPD3/PKD2/APEX1/TPR/FEN1/SRC/NPM1/KIF20B/MAPRE3/AIF1
GOBP_REGULATION_OF_MITOTIC_CELL_CYCLE	-0.3834	-1.6997	0.0010	0.0317	ABL1/IL1B/CCND2/SLC9A3R1/ANXA1/CDC27/CASP2/PPP1R9B/EIF4E/CHEK2/ATF2/STAT5B/EGF/EIF4G1/CDK1/DLGAP5/VPS4B/CENPJ/PTPN6/DYNLT3/MKI67/TP73/BTC/SMPD3/PKD2/APEX1/GIGYF2/CDKN2D/TPR/NBN/NEK7/SIRT1/KIF20B/ASAH2/APPL2/AIF1
GOBP_DNA_METABOLIC_PROCESS	-0.3655	-1.7053	0.0002	0.0115	XRCC4/HDAC8/COMMD1/RFC4/LONP1/RUVBL1/FOS/CHEK2/NPPC/SRPK2/RNF168/PRKD2/CDK1/TP53BP1/WAS/ANXA3/TP73/TDP1/AIFM1/TERF1/SMPD3/APEX1/FH/PARP1/MORF4L1/CDKN2D/BACH1/GATA3/FEN1/HDGFL2/TOP1/TK1/IL4/NBN/NEK7/SRC/NPM1/SIRT1/PMS1/MGMT/BAX/TDRKH/FGFR4/YY1
GOBP_MITOTIC_CELL_CYCLE_PROCESS	-0.3662	-1.7056	0.0003	0.0129	IL1B/CCND2/CHMP6/ANXA1/CDC27/CASP2/CEP85/MZT1/PPP1R9B/EIF4E/PRC1/ID4/CHEK2/ATF2/BECN1/EGF/EIF4G1/CDK1/DLGAP5/VPS4B/BRSK2/SPRY2/LATS1/CENPJ/MITD1/PTPN6/MKI67/OFD1/BTC/SMPD3/PKD2/RHOC/APEX1/GIGYF2/CDKN2D/BACH1/TPR/NBN/DCTN6/DYNLT1/KIF20B/ARHGEF10/TACC3/REEP4/APPL2/AIF1
GOBP_REGULATION_OF_CELL_CYCLE_PROCESS	-0.3703	-1.7175	0.0005	0.0216	DYNC1H1/MDM1/IL1B/CCND2/CHMP6/ANXA1/CDC27/CASP2/CEP85/PPP1R9B/NUDT16/HDAC8/PRC1/CHEK2/ATF2/BECN1/EGF/EIF4G1/CDK1/LIF/DLGAP5/VPS4B/ENTR1/SOX9/CRNN/TP53BP1/GIT1/CENPJ/PTPN6/MKI67/BTC/TERF1/SMPD3/PKD2/APEX1/GIGYF2/CDKN2D/BCL2L1/TPR/FEN1/NBN/NUBP1/RAB11FIP3/NPM1/SIRT1/KIF20B/TACC3/APPL2/AIF1
GOBP_POSTTRANSCRIPTIONAL_REGULATION_OF_GENE_EXPRESSION	-0.4028	-1.7302	0.0010	0.0314	IGF2BP3/EIF5/CIRBP/EIF4E/KRT17/EIF4G1/CSDE1/SHMT1/PAIP2B/ITGA2/TRAF2/LARP1/TCOF1/CASC3/APEX1/TARBP2/GIGYF2/MAPKAPK2/FMR1/TRDMT1/TPR/EPB41L5/AKT2/TSC1/NPM1/EIF2AK2/BANK1/EIF4G3/EIF2AK3/FOXO3
GOBP_REGULATION_OF_DNA_METABOLIC_PROCESS	-0.4153	-1.7319	0.0016	0.0415	HDAC8/RFC4/RUVBL1/NPPC/RNF168/PRKD2/TP53BP1/WAS/ANXA3/AIFM1/TERF1/PARP1/MORF4L1/GATA3/HDGFL2/IL4/NBN/NEK7/SRC/NPM1/SIRT1/MGMT/BAX/FGFR4/YY1
GOMF_PROTEIN_KINASE_ACTIVITY	-0.3793	-1.7326	0.0004	0.0157	SLK/SRPK2/AKT3/PRKD2/TRIM24/CDK1/FLT3/YES1/IRAK1/BRD2/BRSK2/LATS1/BCR/MAP4K5/ARAF/KIT/DAPK2/MAPKAPK2/MAP2K6/TOP1/CDC42BPB/PRKG1/PRKAG3/AKT2/CSNK1D/NEK7/TXK/SRC/PIKFYVE/RIPK4/FGR/FGFR4/RASSF2/RET/EIF2AK2/EIF2AK3/MAP3K5

GOMF_KINASE_ACTIVITY	-0.3696	-1.7383	0.0002	0.0082	SLK/SRPK2/AKT3/PRKD2/TRIM24/CDK1/FLT3/DGKZ/YES1/IRAK1/PMVK/DGKA/BRD2/BRSK2/LATS1/BCR/MAP4K5/KHK/ARAF/KIT/DAPK2/AK2/MAPKAPK2/NADK/MAP2K6/TOP1/TK1/CDC42BPB/PRKG1/PRKAG3/AKT2/CSNK1D/PFKFB2/NEK7/TXK/SRC/PIKFYVE/RIPK4/FGR/FGFR4/RASSF2/RET/EIF2AK2/EIF2AK3/MAP3K5
GOMF_ADENYL_NUCLEOTIDE_BINDING	-0.3544	-1.7557	0.0000	0.0018	SLK/SRPK2/EIF4G1/AKT3/PRKD2/PSMC3/BAG4/CDK1/FLT3/DGKZ/TWF2/YES1/VPS4B/IRAK1/PMVK/DGKA/BRSK2/NAA80/LATS1/BCR/MAP4K5/SULT1A1/KHK/ARAF/MKI67/MARS1/KIT/TTF2/FARSA/DAPK2/AK2/UBE2Z/MAPKAPK2/PRKAR2A/NADK/MAP2K6/MYO9B/EHD3/TOP1/TK1/MYH9/CDC42BPB/ASS1/PRKG1/PRKAG3/AKT2/NUBP1/CSNK1D/PFKFB2/NEK7/TXK/SRC/PMS1/PIKFYVE/RIPK4/KIF20B/FGR/FGFR4/RET/EIF2AK2/EIF2AK3/NMNAT1/MAP3K5
GOMF_RIBONUCLEOTIDE_BINDING	-0.3484	-1.7622	0.0000	0.0003	RFC4/RAB2B/LONP1/RUVBL1/CHEK2/CNGB3/RAB37/SLK/SRPK2/RAB10/EIF4G1/AKT3/DNM1/PRKD2/PSMC3/CDK1/FLT3/GBP4/DGKZ/TWF2/YES1/VPS4B/IRAK1/PMVK/DGKA/BRSK2/NAA80/GNAS/LATS1/BCR/MAP4K5/SULT1A1/KHK/ARAF/MKI67/GTPBP2/MARS1/ARF6/KIT/TTF2/FARSA/DAPK2/RHOC/AK2/UBE2Z/GIMAP8/RAP1A/MAPKAPK2/PRKAR2A/NADK/PDE5A/MAP2K6/MYO9B/EHD3/TOP1/TK1/MYH9/CDC42BPB/ASS1/GBP1/PRKG1/PRKAG3/AKT2/NUBP1/CSNK1D/RAB27B/PFKFB2/NEK7/CRACR2A/TXK/SRC/PMS1/PIKFYVE/RIPK4/KIF20B/SEPTIN7/FGR/FGFR4/RET/EIF2AK2/EIF2AK3/NMNAT1/MAP3K5/RAB44
GOCC_NUCLEOLUS	-0.3964	-1.7723	0.0002	0.0118	REXO2/STK24/ABL1/PPP1R12A/CCND2/ERI1/CEBPA/CEP85/ZCCHC8/NFKBIE/AGER/NUDT16/TBCA/PPP1CC/CETN3/SRPK2/NUB1/DNAJC21/GOLGA3/TCOF1/PTPN6/PNMA1/MKI67/MARS1/KIT/TERF1/NFX1/MTDH/APEX1/PARP1/FMR1/PPM1B/FEN1/EEF1D/TOP1/NBN/EDF1/NPM1/SIRT1/SNRPB2/KIF20B/WDR46/MNDA
GOMF_TRANSCRIPTION_COREGULATOR_ACTIVITY	-0.4414	-1.7748	0.0011	0.0353	IL31RA/MAGED1/RALY/CRYM/RUVBL1/PXN/BIRC2/PCBD1/MED18/TRIM24/TP53BP1/CENPJ/HDAC9/PSIP1/KDM3A/MTDH/APEX1/HDGFL2/EDF1/NPM1/SIRT1/RBPMS/TRIM25/CALCOCO1/HDGF
GOMF_CYTOSKELETAL_PROTEIN_BINDING	-0.3677	-1.7815	0.0000	0.0019	FXYD5/FKBP4/IQGAP2/HCLS1/PPP1R9B/TBCC/MYOM2/ANXA2/PDLIM7/S100A4/TBCA/PRC1/GC/SSH3/PXN/WASF3/CETN3/MTSS2/SNX5/VASP/RAB10/DNM1/LSP1/NEXN/NPHS1/CLIP2/DLGAP5/TWF2/DBNL/VASH1/BRSK2/BCL7B/FMNL1/DLG4/GIT1/CENPJ/DMD/NFKB1/WAS/PHACTR2/GMFG/OFD1/PSTPIP2/TERF1/PKD2/CORO1A/MICALL2/CACYBP/INPPL1/FMR1/TPR/MTSS1/MYO9B/LMOD2/BLOC1S2/TPPP2/CGN/EPB41L5/MYH9/TOR1AIP1/GBP1/RAB27B/PDLIM5/KIF20B/OPHN1/DAAM1/ARHGEF10/REEP4/WASL/SMTN/CAMSAP1/IRAG2/MAPRE3/AFAP1/AIF1
GOBP_MITOTIC_CELL_CYCLE	-0.3789	-1.7919	0.0000	0.0018	TADA3/DYNC1H1/ABL1/PPP1R12A/IL1B/CCND2/CHMP6/SLC9A3R1/ANXA1/CDC27/CASP2/CEP85/MZT1/PPP1R9B/EIF4E/PRC1/ID4/CHEK2/ATF2/ZNRD2/BECN1/STAT5B/EGF/SUGT1/EIF4G1/CDK1/DLGAP5/VPS4B/BRSK2/SPRY2/LATS1/CENPJ/MITD1/PTPN6/DYNLT3/MKI67/TP73/OFD1/BTC/SMPD3/PKD2/RHOC/APEX1/GIGYF2/CDKN2D/BACH1/TPR/NBN/DCTN6/NEK7/SIRT1/DYNLT1/KIF20B/ARHGEF10/TACC3/ASAH2/REEP4/AZI2/APPL2/AIF1
GOBP_AMIDE_BIOSYNTHETIC_PROCESS	-0.3939	-1.7951	0.0002	0.0082	DMD/LARP1/TCOF1/MRPL58/GTPBP2/MARS1/EIF1AX/FARSA/CASC3/TARBP2/PPT1/GIGYF2/FMR1/EIF2S2/TPR/EEF1D/ASS1/AKT2/TSC1/TNIP1/NPM1/ASAH2/EIF2AK2/BANK1/EIF4G3/EIF2AK3/FOXO3/PM20D1
GOMF_TRANSFERASE_ACTIVITY_TRANSFERRING_PHOSPHORUS_CONTAINING_GROUPS	-0.3799	-1.7962	0.0000	0.0032	SLK/SRPK2/AKT3/PRKD2/TRIM24/CDK1/FLT3/DGKZ/YES1/IRAK1/PMVK/DGKA/BRD2/BRSK2/LATS1/BCR/MAP4K5/KHK/ARAF/KIT/TERF1/DAPK2/AK2/MAPKAPK2/NADK/MAP2K6/GYS1/TOP1/TK1/CDC42BPB/PRKG1/PRKAG3/AKT2/CSNK1D/PFKFB2/NEK7/TXK/SRC/PIKFYVE/RIPK4/FGR/FGFR4/RASSF2/RET/EIF2AK2/EIF2AK3/NMNAT1/MAP3K5
GOBP_GERM_CELL_DEVELOPMENT	-0.4782	-1.8039	0.0021	0.0488	KIT/KDM3A/TARBP2/BCL2L1/PDE5A/FABP9/SRC/BAX/TDRKH/FOXO3/ZP3/PAEP
GOCC_RUFFLE_MEMBRANE	-0.5410	-1.8101	0.0018	0.0460	EPB41L5/PLEKHO1/AKT2/ARHGAP45/SRC/FGR/APPL2/AIF1
GOBP_REGULATION_OF_CYCLIN_DEPENDENT_PROTEIN_KINASE_ACTIVITY	-0.5755	-1.8140	0.0020	0.0488	CCNE1/RGCC/PSRC1/EGFR/CDKN1A/CCND2/CEBPA/LATS1/HHEX/CASP3/PKD2/CDKN2D/HEXIM1/SRC/MAPRE3
GOCC_MICROTUBULE_ORGANIZING_CENTER	-0.3877	-1.8200	0.0000	0.0032	CEP152/CDK1/DLGAP5/YES1/NIT2/VPS4B/ENTR1/BRSK2/TXNDC9/GIT1/LATS1/CENPJ/PDZD2/RILPL2/OFD1/DCDC2C/DDHD2/PKD2/APEX1/EVI5/MAPKAPK2/BCL2L1/PRKAR2A/BLOC1S2/DCTN6/CEP170/NUBP1/CSNK1D/RAB11FIP3/NEK7/CRACR2A/PPP2R5A/NPM1/KIF20B/SDCCAG8/DAAM1/ARHGEF10/TACC3/SMTN/RPGR/IRAG2/MAPRE3/SLC1A4
GOBP_NEGATIVE_REGULATION_OF_CELLULAR_MACROMOLECULE_BIOSYNTHETIC_PROCESS	-0.4894	-1.8202	0.0021	0.0488	IGF2BP3/CIRBP/EIF4E/NPPC/S100A11/EIF4G1/CSDE1/SHMT1/PAIP2B/LARP1/TERF1/GIGYF2/FMR1/TPR/TSC1/SRC/EIF2AK2/BANK1/EIF2AK3
GOMF_GTPASE_ACTIVATOR_ACTIVITY	-0.4920	-1.8248	0.0009	0.0304	BNIP2/CHM/IQGAP2/MTSS2/ARHGEF12/VAV3/RGS10/JUN/ARHGAP1/GIT1/BCR/RABEP1/ARHGEF1/EVI5/MYO9B/ARHGAP45/FAM13A/TBC1D5/OPHN1/SYNGAP1
GOBP_CELL_CYCLE_PROCESS	-0.3714	-1.8277	0.0000	0.0005	SEPTIN9/DYNC1H1/MDM1/PPP1R12A/IL1B/CCND2/ZBTB17/CHMP6/ANXA1/CDC27/CASP2/CEP85/MZT1/IST1/PPP1R9B/NUDT16/EIF4E/HDAC8/PRC1/ID4/CHEK2/ATF2/BECN1/CETN3/EGF/EIF4G1/CEP152/FOXJ3/CDK1/LIF/DLGAP5/VPS4B/ENTR1/SOX9/CRNN/BRSK2/SPRY2/TP53BP1/GIT1/LATS1/CENPJ/MITD1/PTPN6/MKI67/OFD1/BTC/TERF1/SMPD3/PKD2/RHOC/APEX1/GIGYF2/CDKN2D/BCL2L1/BACH1/TPR/FEN1/MYH9/NBN/DCTN6/NUBP1/CSNK1D/RAB11FIP3/NEK7/NPM1/SIRT1/DYNLT1/KIF20B/SDCCAG8/SEPTIN7/TDRKH/ARHGEF10/TACC3/REEP4/WASL/APPL2/MAPRE3/AIF1
GOBP_PEPTIDE_BIOSYNTHETIC_PROCESS	-0.4134	-1.8328	0.0001	0.0077	MCTS1/IGF2BP3/EIF5/IGHMBP2/CIRBP/EIF4E/KRT17/EIF4G1/CSDE1/SHMT1/PAIP2B/ITGA2/DMD/LARP1/TCOF1/MRPL58/GTPBP2/MARS1/EIF1AX/FARSA/CASC3/TARBP2/GIGYF2/FMR1/EIF2S2/TPR/EEF1D/AKT2/TSC1/TNIP1/NPM1/EIF2AK2/BANK1/EIF4G3/EIF2AK3/FOXO3
GOBP_METHYLATION	-0.5633	-1.8405	0.0013	0.0375	COMT/KDM3A/PARP1/TRDMT1/GATA3/MTR/NFYA/SIRT1/MGMT/TDRKH
GOBP_CHROMATIN_ASSEMBLY_OR_DISASSEMBLY	-0.6141	-1.8496	0.0020	0.0488	HDAC8/FAM172A/SOX9/BRD2/AXIN1/PADI4/TPR/NFE2/NPM1/SIRT1
GOBP_CHROMOSOME_SEGREGATION	-0.5005	-1.8561	0.0007	0.0250	CCNE1/TOP2B/PSRC1/DCTN2/CHMP1A/CENPF/PIBF1/CDC26/BAG6/NUDC/DYNC1H1/CHMP6/CDC27/CEP85/HDAC8/PRC1/BECN1/DLGAP5/VPS4B/LATS1/MKI67/TERF1/TPR/FEN1/TOP1/TACC3
GOCC_CONDENSED_CHROMOSOME	-0.5547	-1.8562	0.0012	0.0367	DCTN1/MAD1L1/SETMAR/DCTN2/CHMP1A/CENPF/PPP1R12A/CHMP6/XRCC4/PPP1CC/SUGT1/TP53BP1/CEBPB/DYNLT3/MKI67/TPR/DCTN6/SEPTIN7/RASSF2
GOMF_TUBULIN_BINDING	-0.4650	-1.8587	0.0005	0.0192	TBCC/TBCA/PRC1/CETN3/DNM1/CLIP2/DLGAP5/GIT1/CENPJ/OFD1/TERF1/CACYBP/FMR1/TPR/BLOC1S2/TPPP2/KIF20B/REEP4/CAMSAP1/IRAG2/MAPRE3
GOCC_MIDBODY	-0.5159	-1.8647	0.0017	0.0437	NUDC/CHMP6/IL16/IST1/ANXA2/PRC1/PPP1CC/CDK1/VPS4B/ENTR1/TXNDC9/LATS1/MITD1/ARF6/SPART/RAB11FIP3/KIF20B/SEPTIN7/MAPRE3
GOBP_ORGANELLE_FUSION	-0.5303	-1.8701	0.0007	0.0250	VAV3/CDK1/SNAP23/VAMP8/CORO1A/STX8/STX7/STX5/STX6/STX16/AKT2/PIKFYVE/BAX/IRAG2/DOC2B

GOBP_ORGANELLE_MEMBRANE_FUSION	-0.5598	-1.8731	0.0010	0.0323	SNAP23/VAMP8/CORO1A/STX8/STX7/STX5/STX6/STX16/AKT2/PIKFYVE/IRAG2/DOC2B
GOCC_MICROTUBULE_CYTOSKELETON	-0.3785	-1.8754	0.0000	0.0002	CEP152/STAU1/CDK1/CLIP2/DLGAP5/YES1/NIT2/NEDD9/VPS4B/ENTR1/XIAP/ACOT13/BRSK2/SPRY2/TXNDC9/GIT1/LATS1/CENPJ/TPT1/AXIN1/PDZD2/RILPL2/DYNLT3/OFD1/DCDC2C/TERF1/DDHD2/PKD2/APEX1/EVI5/MAPKAPK2/MYCBP2/BCL2L1/PRKAR2A/TPR/BLOC1S2/TPPP2/MYH9/IKBKG/DCTN6/CEP170/NUBP1/CSNK1D/RAB11FIP3/NEK7/CRACR2A/PPP2R5A/NPM1/DYNLT1/KIF20B/SDCCAG8/SEPTIN7/DAAM1/ARHGEF10/TACC3/REEP4/SMTN/CAMSAP1/RPGR/IRAG2/MAPRE3/ODAM/SLC1A4
GOBP_SPINDLE_ORGANIZATION	-0.5344	-1.8779	0.0014	0.0396	PSRC1/DCTN2/CHMP1A/PIBF1/NUDC/DYNC1H1/CHMP6/MZT1/PRC1/CHEK2/DLGAP5/VPS4B/CENPJ/OFD1/TPR/MYH9/DCTN6/CSNK1D/NEK7/ARHGEF10/TACC3/MAPRE3
GOBP_REGULATION_OF_ENDOPLASMIC_RETICULUM_STRESS_INDUCED_INTRINSIC_APOPTOTIC_SIGNALING_PATHWAY	-0.7380	-1.8859	0.0021	0.0489	ERP29/BCL2L1/PTPN1/SIRT1/EIF2AK3
GOBP_REGULATION_OF_RESPONSE_TO_ENDOPLASMIC_RETICULUM_STRESS	-0.6115	-1.8938	0.0018	0.0465	USP25/ERP29/BCL2L1/MANF/PTPN1/SIRT1/BAX/EIF2AK3
GOBP_CELL_CYCLE	-0.3728	-1.9009	0.0000	0.0000	SEPTIN9/TADA3/DYNC1H1/MDM1/ABL1/PPP1R12A/IL1B/CCND2/DUSP13/ZBTB17/CHMP6/SLC9A3R1/ANXA1/CDC27/PAK4/CEBPA/CASP2/CEP85/MCTS1/MZT1/IST1/PPP1R9B/NUDT16/EIF4E/HDAC8/PRC1/PPP1CC/ID4/RUVBL1/CHEK2/ATF2/ZNRD2/BIRC2/BECN1/CETN3/STAT5B/NPPC/SRPK2/EGF/SUGT1/EIF4G1/CEP152/FOXJ3/CDK1/LIF/DLGAP5/NEDD9/VPS4B/ENTR1/JUN/SOX9/XIAP/VASH1/CRNN/BRSK2/SPRY2/TP53BP1/GIT1/LATS1/BCR/CENPJ/HHEX/MITD1/PTPN6/DYNLT3/MKI67/TP73/OFD1/ARF6/CASP3/BTC/TERF1/SMPD3/PKD2/RHOC/APEX1/ING1/MORF4L1/GIGYF2/EVI5/CDKN2D/BCL2L1/BACH1/HEXIM1/GATA3/TPR/MAP2K6/FEN1/MYH9/NBN/DCTN6/AKT2/NUBP1/TSC1/CSNK1D/RAB11FIP3/NEK7/SRC/NPM1/SIRT1/DYNLT1/KIF20B/SDCCAG8/BAX/SEPTIN7/TDRKH/ARHGEF10/TACC3/YY1/ASAH2/REEP4/WASL/AZI2/RASSF2/APPL2/MAPRE3/AIF1
GOBP_CELLULAR_RESPONSE_TO_DNA_DAMAGE_STIMULUS	-0.4174	-1.9067	0.0000	0.0029	XRCC4/COMMD1/RFC4/SPRED2/RUVBL1/CHEK2/ATF2/VAV3/RNF168/CDK1/XIAP/YJU2/TP53BP1/TPT1/WAS/TP73/TDP1/CASP3/APEX1/FOXO1/FH/PARP1/MORF4L1/GIGYF2/CASP9/MAPKAPK2/CDKN2D/FMR1/BCL2L1/BACH1/MAP2K6/FEN1/HDGFL2/NBN/IKBKG/NPM1/SIRT1/PMS1/MGMT/BAX/YY1/FOXO3/MNDA
GOBP_DNA_PACKAGING	-0.6176	-1.9129	0.0014	0.0398	FAM172A/CDK1/SOX9/BRD2/AXIN1/AIFM1/PADI4/TPR/NPM1/SIRT1
GOMF_NUCLEOSIDE_TRIPHOSPHATASE_REGULATOR_ACTIVITY	-0.4664	-1.9186	0.0002	0.0086	BNIP2/CHM/IQGAP2/EIF5/DNMBP/MTSS2/EGF/ARHGEF12/VAV3/RGS10/JUN/ARHGAP1/RGL2/GIT1/BCR/WAS/RABEP1/ARHGEF1/FGD3/EVI5/RAP1A/MYCBP2/MYO9B/EEF1D/ARHGAP45/FAM13A/TBC1D5/OPHN1/SYNGAP1/ARHGEF10/WASL/RPGR
GOBP_INTRINSIC_APOPTOTIC_SIGNALING_PATHWAY_IN_RESPONSE_TO_ENDOPLASMIC_RETICULUM_STRESS	-0.5885	-1.9195	0.0015	0.0399	BRSK2/CEBPB/TRAF2/AIFM1/ERP29/BCL2L1/PTPN1/SIRT1/BAX/EIF2AK3/MAP3K5
GOMF_CYTOSKELETAL_MOTOR_ACTIVITY	-0.7326	-1.9252	0.0019	0.0465	MYO9B/MYH9/KIF20B/MYL6B
GOMF_DNA_BINDING_TRANSCRIPTION_REPRESSOR_ACTIVITY	-0.5755	-1.9259	0.0006	0.0226	JUN/CEBPB/HHEX/NFKB1/MAX/NFATC3/LRRFIP1/NFX1/FOXO1/BACH1/GATA3/NACC1/HDGF/YY1/FOXO3
GOBP_CHROMOSOME_ORGANIZATION	-0.4282	-1.9319	0.0000	0.0020	TOP1MT/CENPF/PIBF1/CDC26/BRD1/BAG6/NUDC/ABL1/CHMP6/ANXA1/CDC27/BRD3/CBX2/IGHMBP2/HDAC8/PRC1/RFC4/RUVBL1/BECN1/FAM172A/SATB1/RNF168/CDK1/DLGAP5/VPS4B/SOX9/BRD2/LATS1/HHEX/AXIN1/MKI67/AIFM1/HDAC9/TERF1/KDM3A/APEX1/PARP1/PADI4/GATA3/TPR/FEN1/TOP1/NFE2/NBN/NEK7/SRC/NPM1/SIRT1/TACC3/YY1/ITPA
GOMF_RNA_POLYMERASE_II_SPECIFIC_DNA_BINDING_TRANSCRIPTION_FACTOR_BINDING	-0.4846	-1.9362	0.0002	0.0082	CRX/STK4/TADA3/FKBP4/CEBPA/HCLS1/ID4/FOS/ATF2/STAT5B/ANXA4/CRKL/WWP2/TRIM24/FLT3/JUN/TP53BP1/LATS1/CEBPB/TP73/HDAC9/KDM3A/MTDH/APEX1/ARNT/PARP1/GATA3/NFATC1/SRC/NPM1/SIRT1
GOCC_CONDENSED_CHROMOSOME_CENTROMERIC_REGION	-0.6341	-1.9411	0.0012	0.0373	DCTN2/CHMP1A/CENPF/PPP1R12A/CHMP6/PPP1CC/SUGT1/TP53BP1/CEBPB/DYNLT3/TPR/DCTN6/SEPTIN7/RASSF2
GOBP_CHROMATIN_ORGANIZATION	-0.5065	-1.9419	0.0006	0.0237	BRD3/CBX2/HDAC8/RUVBL1/FAM172A/SATB1/RNF168/SOX9/BRD2/AXIN1/MKI67/HDAC9/KDM3A/PADI4/GATA3/TPR/TOP1/NFE2/NPM1/SIRT1/YY1
GOCC_RIBONUCLEOPROTEIN_COMPLEX	-0.4834	-1.9458	0.0001	0.0055	ERI1/PSMA1/DDX58/ZCCHC8/IGHMBP2/CIRBP/RALY/EIF4E/RUVBL1/RRP15/EIF4G1/SUGP1/SRP14/YJU2/CWC15/LARP1/MRPL58/TTF2/CASC3/TARBP2/FMR1/HEXIM1/NPM1/SNRPB2/WDR46/SMNDC1/SART1/LSM8
GOBP_RECOMBINATIONAL_REPAIR	-0.6874	-1.9519	0.0010	0.0319	RUVBL1/TP53BP1/WAS/PARP1/MORF4L1/FEN1/HDGFL2/NBN/YY1
GOBP_REGULATION_OF_VIRAL_GENOME_REPLICATION	-0.6540	-1.9623	0.0020	0.0488	SRPK2/STAU1/LARP1/TARBP2/TNIP1/MAVS/EIF2AK2
GOCC_CYTOPLASMIC_STRESS_GRANULE	-0.6421	-1.9655	0.0009	0.0299	CIRBP/EIF4E/EIF4G1/CSDE1/STAU1/LARP1/CASC3/GIGYF2/FMR1/RBPMS/TRIM25
GOBP_SPINDLE_ASSEMBLY	-0.6375	-1.9742	0.0007	0.0246	CHMP1A/PIBF1/DYNC1H1/CHMP6/MZT1/PRC1/CHEK2/VPS4B/OFD1/TPR/CSNK1D/NEK7/ARHGEF10/MAPRE3
GOCC_SPLICEOSOMAL_COMPLEX	-0.6317	-1.9755	0.0007	0.0246	ZCCHC8/CIRBP/RALY/SUGP1/YJU2/CWC15/TTF2/CASC3/SNRPB2/SMNDC1/SART1/LSM8
GOBP_REGULATION_OF_TRANSLATIONAL_INITIATION	-0.6146	-1.9770	0.0004	0.0186	EIF4G1/CSDE1/PAIP2B/LARP1/FMR1/TPR/NPM1/EIF2AK2/BANK1/EIF4G3/EIF2AK3
GOMF_TRANSLATION_FACTOR_ACTIVITY_RNA_BINDING	-0.7024	-1.9946	0.0005	0.0216	MCTS1/EIF5/EIF4E/EIF4G1/MRPL58/GTPBP2/EIF1AX/EIF2S2/EEF1D/EIF4G3
GOBP_REGULATION_OF_CELL_CYCLE	-0.4070	-1.9948	0.0000	0.0000	TADA3/DYNC1H1/MDM1/ABL1/IL1B/CCND2/ZBTB17/CHMP6/SLC9A3R1/ANXA1/CDC27/CEBPA/CASP2/CEP85/PPP1R9B/NUDT16/EIF4E/HDAC8/PRC1/RUVBL1/CHEK2/ATF2/BIRC2/BECN1/STAT5B/NPPC/SRPK2/EGF/EIF4G1/CDK1/LIF/DLGAP5/VPS4B/ENTR1/JUN/SOX9/XIAP/CRNN/TP53BP1/GIT1/LATS1/BCR/CENPJ/HHEX/PTPN6/DYNLT3/MKI67/TP73/CASP3/BTC/TERF1/SMPD3/PKD2/APEX1/MORF4L1/GIGYF2/CDKN2D/BCL2L1/HEXIM1/GATA3/TPR/MAP2K6/FEN1/NBN/AKT2/NUBP1/TSC1/RAB11FIP3/NEK7/SRC/NPM1/SIRT1/KIF20B/BAX/TACC3/YY1/ASAH2/APPL2/MAPRE3/AIF1
GOMF_PROTEIN_SERINE_THREONINE_TYROSINE_KINASE_ACTIVITY	-0.4688	-2.0060	0.0000	0.0018	SLK/SRPK2/AKT3/PRKD2/CDK1/FLT3/YES1/IRAK1/BRSK2/LATS1/BCR/MAP4K5/ARAF/KIT/DAPK2/MAPKAPK2/MAP2K6/CDC42BPB/AKT2/CSNK1D/NEK7/TXK/SRC/PIKFYVE/RIPK4/FGR/FGFR4/RET/EIF2AK2/EIF2AK3/MAP3K5

GOCC_NUCLEAR_BODY	-0.4540	-2.0085	0.0000	0.0008	ZCCHC8/MOCS2/IL16/ZBTB16/IGHMBP2/PPP1CC/CHEK2/SRPK2/SATB1/CALCOCO2/DGKZ/SAFB2/BRD2/MORC3/BNIP3L/CWC15/TP53BP1/MAX/TREML1/MKI67/TERF1/MTDH/CASC3/APEX1/ARNT/TARBP2/PARP1/INPPL1/MORF4L1/FMR1/NFE2/NBN/KAZN/NFATC1/LRCH4/SIRT1/SNRPB2/TRIM25/SMNDC1/MINDY1/SART1/NMNAT1
GOBP_ENDOSOMAL_TRANSPORT	-0.5292	-2.0144	0.0002	0.0093	SNX5/VPS53/RAB10/ARFIP1/STAM/VPS4B/ENTR1/GGA1/ARHGAP1/SNX2/TBC1D23/WAS/ARF6/MICALL2/STX5/STX6/EVI5/STX16/BLOC1S2/EHD3/RAB11FIP3/PIKFYVE/TBC1D5
GOCC_CHROMOSOME_CENTROMERIC_REGION	-0.6315	-2.0313	0.0002	0.0114	DCTN2/CHMP1A/CENPF/PPP1R12A/CHMP6/PPP1CC/SUGT1/TP53BP1/CEBPB/DYNLT3/FMR1/TPR/DCTN6/PPP2R5A/SEPTIN7/RASSF2
GOMF_CHROMATIN_BINDING	-0.5098	-2.0528	0.0000	0.0013	FOS/ATF2/STAT5B/RNF168/TRIM24/CDK1/FABP1/JUN/SOX9/BRD2/CEBPB/NFKB1/GLYR1/KDM3A/APEX1/FOXO1/MORF4L1/FMR1/TPR/TOP1/NPM1/SIRT1/CALCOCO1/YY1/FOXO3
GOMF_DNA_BINDING_TRANSCRIPTION_ACTIVATOR_ACTIVITY	-0.5539	-2.0544	0.0001	0.0036	SMAD1/CRX/ZBTB17/CEBPA/ZBTB16/FOS/ATF2/STAT5B/FOXJ3/JUN/SOX9/CEBPB/HHEX/NFKB1/NFATC3/TP73/FOXO1/BACH1/GATA3/NFATC1/YY1/FOXO3/NFAT5
GOBP_TRANSLATIONAL_INITIATION	-0.6037	-2.0603	0.0001	0.0076	EIF4G1/CSDE1/PAIP2B/LARP1/EIF1AX/FMR1/EIF2S2/TPR/NPM1/EIF2AK2/BANK1/EIF4G3/EIF2AK3
GOMF_DNA_BINDING_TRANSCRIPTION_FACTOR_BINDING	-0.4857	-2.0615	0.0000	0.0012	CRX/STK4/TBL1X/TADA3/ZBTB17/FKBP4/CEBPA/HCLS1/EIF4E/HDAC8/ID4/FOS/ATF2/STAT5B/ANXA4/CRKL/WWP2/TRIM24/FLT3/JUN/SOX9/TP53BP1/LATS1/CEBPB/HHEX/TPT1/MAX/TP73/HDAC9/KDM3A/MTDH/APEX1/ARNT/PARP1/GATA3/NBN/NFATC1/SRC/NPM1/SIRT1/YY1
GOCC_MICROTUBULE	-0.5167	-2.0654	0.0000	0.0020	NUDC/SEPTIN9/DYNC1H1/MDM1/CHMP6/FKBP4/TBCC/TBCA/BAIAP2/PRC1/TBCB/DNM1/CDK1/CLIP2/XIAP/SPRY2/CENPJ/TPT1/DYNLT3/DCDC2C/TPPP2/CEP170/CSNK1D/NEK7/DYNLT1/KIF20B/REEP4/CAMSAP1/MAPRE3
GOCC_PROTEIN_DNA_COMPLEX	-0.6913	-2.0821	0.0001	0.0080	RUVBL1/FOS/HHEX/MAX/GLYR1/TERF1/PARP1/MORF4L1/TOP1/NFE2/NFYA/NPM1
GOMF_TRANSCRIPTION_FACTOR_BINDING	-0.4723	-2.0893	0.0000	0.0002	CRX/STK4/TBL1X/TADA3/ZBTB17/FKBP4/CEBPA/HCLS1/ZBTB16/IGHMBP2/EIF4E/BAIAP2/HDAC8/ID4/RUVBL1/FOS/ATF2/STAT5B/ANXA4/CRKL/WWP2/TRIM24/FLT3/JUN/SOX9/TP53BP1/LATS1/CEBPB/HHEX/TPT1/MAX/NFATC3/TP73/HDAC9/KDM3A/MTDH/APEX1/ARNT/PARP1/GATA3/NBN/NFATC1/EDF1/SRC/NPM1/SIRT1/HDGF/YY1/FOXO3/NFAT5
GOBP_RETROGRADE_TRANSPORT_ENDOSOME_TO_GOLGI	-0.6957	-2.0955	0.0001	0.0076	SNX5/VPS53/ARFIP1/GGA1/SNX2/TBC1D23/STX5/STX6/EVI5/STX16/EHD3/PIKFYVE/TBC1D5
GOCC_CHROMATIN	-0.4710	-2.0991	0.0000	0.0002	ZNF174/BRD3/CBX2/CEBPA/BCL7A/IST1/MAGED1/RUVBL1/FOS/ATF2/ZHX2/STAT5B/SRPK2/SATB1/TRIM24/FOXJ3/JUN/SOX9/BRD2/BCL7B/CEBPB/HHEX/NFKB1/MAX/NFATC3/TP73/GLYR1/PSIP1/NFX1/KDM3A/STAT2/ARNT/FOXO1/MORF4L1/BACH1/GATA3/NFE2/NFATC1/NFYA/SIRT1/CALCOCO1/YY1/FOXO3/NFAT5
GOBP_CYTOSOLIC_TRANSPORT	-0.6021	-2.1046	0.0001	0.0063	BECN1/SNX5/VPS53/ARFIP1/GGA1/SNX2/TBC1D23/CORO1A/STX8/STX5/STX6/EVI5/STX16/EHD3/SRC/PIKFYVE/TBC1D5
GOCC_SPINDLE	-0.5156	-2.1142	0.0000	0.0004	CDK1/DLGAP5/NEDD9/VPS4B/XIAP/ACOT13/GIT1/LATS1/TPT1/DYNLT3/TERF1/PKD2/EVI5/TPR/MYH9/IKBKG/CEP170/CSNK1D/NEK7/NPM1/DYNLT1/KIF20B/SEPTIN7/TACC3/IRAG2/MAPRE3/ODAM
GOMF_SEQUENCE_SPECIFIC_DNA_BINDING	-0.4816	-2.1352	0.0000	0.0002	CRX/TBL1X/ABL1/ZBTB17/IKZF2/ZNF174/LMNB1/CEBPA/ZBTB16/LONP1/FOS/ATF2/STAT5B/SATB1/TRIM24/FOXJ3/SAFB2/JUN/SOX9/TP53BP1/TBR1/CEBPB/HHEX/NFKB1/MAX/NFATC3/TP73/LRRFIP1/TERF1/NFX1/STAT2/APEX1/ARNT/FOXO1/BACH1/GATA3/ZNF75D/TOP1/NFE2/NFATC1/NACC1/NFYA/NPM1/SIRT1/CALCOCO1/HDGF/YY1/FOXO3/NFAT5
GOMF_TRANSLATION_REGULATOR_ACTIVITY_NUCLEIC_ACID_BINDING	-0.7149	-2.1451	0.0001	0.0079	MCTS1/EIF5/EIF4E/EIF4G1/SHMT1/PAIP2B/LARP1/MRPL58/GTPBP2/EIF1AX/EIF2S2/EEF1D/EIF4G3
GOMF_DNA_BINDING_TRANSCRIPTION_FACTOR_ACTIVITY	-0.5291	-2.1498	0.0000	0.0008	ZBTB17/IKZF2/ZNF174/CEBPA/ZBTB16/FOS/ATF2/ZHX2/STAT5B/SATB1/FOXJ3/JUN/SOX9/TBR1/CEBPB/HHEX/NFKB1/MAX/NFATC3/TP73/LRRFIP1/NFX1/STAT2/ARNT/FOXO1/BACH1/GATA3/ZNF75D/NFE2/NFATC1/NACC1/NFYA/HDGF/YY1/FOXO3/NFAT5
GOCC_NUCLEAR_PROTEIN_CONTAINING_COMPLEX	-0.4840	-2.1818	0.0000	0.0001	CHMP1A/PAGR1/PPIE/CREB3/POLR2A/CDKN1A/CDC26/BRD1/GPKOW/SMAD1/LAT/CRX/TBL1X/TADA3/CHMP6/ERI1/CDC27/CBX2/CEBPA/BCL7A/ZCCHC8/CIRBP/RALY/XRCC4/HDAC8/RUVBL1/FOS/ATF2/CETN3/SUGP1/MED18/JUN/YJU2/CWC15/BCL7B/CEBPB/MAX/TTF2/HDAC9/TERF1/KDM3A/STAT2/CASC3/ELOA/ARNT/MORF4L1/BACH1/HEXIM1/TPR/NFE2/NBN/NFYA/SIRT1/SNRPB2/YY1/SMNDC1/SART1/LSM8/FOXO3
GOMF_MODIFICATION_DEPENDENT_PROTEIN_BINDING	-0.6475	-2.1843	0.0000	0.0018	RNF168/TRIM24/BRD2/MORC3/TP53BP1/PTPN6/GLYR1/ING1/FMR1/UFD1/HDGFL2/IKBKG/TAB2/MINDY1
GOMF_PROTEIN_SERINE_THREONINE_KINASE_ACTIVITY	-0.5471	-2.1871	0.0000	0.0007	IRAK4/CHEK2/SLK/SRPK2/AKT3/PRKD2/CDK1/IRAK1/BRD2/BRSK2/LATS1/BCR/MAP4K5/ARAF/DAPK2/MAPKAPK2/MAP2K6/TOP1/CDC42BPB/PRKG1/PRKAG3/AKT2/CSNK1D/NEK7/PIKFYVE/RIPK4/EIF2AK2/EIF2AK3/MAP3K5
GOCC_TRANSCRIPTION_REGULATOR_COMPLEX	-0.5684	-2.2084	0.0000	0.0012	SMAD1/CRX/TBL1X/TADA3/CEBPA/HCLS1/ZBTB16/FOS/ATF2/MED18/JUN/SOX9/CEBPB/MAX/NFATC3/HDAC9/STAT2/APEX1/ARNT/PARP1/BACH1/NFE2/NFATC1/NFYA/HDGF/YY1/FOXO3/NFAT5
GOCC_SPINDLE_POLE	-0.7051	-2.2225	0.0000	0.0010	CEP85/PRC1/DLGAP5/NEDD9/VPS4B/GIT1/LATS1/TPT1/IKBKG/NEK7/NPM1/KIF20B/TACC3/IRAG2
GOCC_CHROMOSOMAL_REGION	-0.6052	-2.2331	0.0000	0.0015	PPP1CC/CHEK2/SUGT1/CDK1/TP53BP1/CEBPB/DYNLT3/TERF1/APEX1/PARP1/FMR1/TPR/FEN1/NBN/DCTN6/PPP2R5A/SEPTIN7/RASSF2
GOMF_TRANSLATION_REGULATOR_ACTIVITY	-0.6868	-2.2401	0.0000	0.0020	MCTS1/IGF2BP3/EIF5/CIRBP/EIF4E/EIF4G1/SHMT1/PAIP2B/LARP1/MRPL58/GTPBP2/EIF1AX/FMR1/EIF2S2/EEF1D/EIF2AK2/EIF4G3/EIF2AK3
GOMF_CIS_REGULATORY_REGION_SEQUENCE_SPECIFIC_DNA_BINDING	-0.5446	-2.2402	0.0000	0.0002	FOS/ATF2/STAT5B/SATB1/TRIM24/FOXJ3/JUN/SOX9/TBR1/CEBPB/HHEX/NFKB1/MAX/NFATC3/TP73/LRRFIP1/STAT2/ARNT/FOXO1/BACH1/GATA3/ZNF75D/TOP1/NFE2/NFATC1/NACC1/NFYA/SIRT1/CALCOCO1/HDGF/YY1/FOXO3/NFAT5
GOMF_TRANSCRIPTION_REGULATOR_ACTIVITY	-0.4895	-2.2582	0.0000	0.0000	SMAD1/TRIM5/CRX/TBL1X/TADA3/ABL1/ZBTB17/IKZF2/ZNF174/CEBPA/ZBTB16/IL31RA/MAGED1/RALY/CRYM/ID4/RUVBL1/FOS/ATF2/PXN/BIRC2/PCBD1/ZHX2/STAT5B/SATB1/MED18/TRIM24/FOXJ3/JUN/SOX9/TP53BP1/TBR1/CENPJ/CEBPB/HHEX/NFKB1/MAX/NFATC3/TP73/LRRFIP1/HDAC9/PSIP1/NFX1/KDM3A/MTDH/STAT2/APEX1/ARNT/FOXO1/BACH1/GATA3/ZNF75D/HDGFL2/NFE2/NFATC1/NACC1/EDF1/NFYA/NPM1/SIRT1/RBPMS/TRIM25/CALCOCO1/HDGF/YY1/FOXO3/NFAT5
GOMF_PROTEIN_SERINE_KINASE_ACTIVITY	-0.6246	-2.3562	0.0000	0.0002	STK4/STK24/PKN3/PAK4/IRAK4/CHEK2/SLK/SRPK2/AKT3/PRKD2/CDK1/IRAK1/BRSK2/LATS1/BCR/MAP4K5/ARAF/DAPK2/MAPKAPK2/MAP2K6/CDC42BPB/AKT2/CSNK1D/NEK7/PIKFYVE/RIPK4/EIF2AK2/EIF2AK3/MAP3K5
GOMF_RNA_BINDING	-0.4743	-2.3582	0.0000	0.0000	ERI1/PSMA1/DDX58/DXO/FKBP4/EPPK1/MCTS1/ZCCHC8/IGF2BP3/EIF5/IGHMBP2/CIRBP/ANXA2/S100A4/NUDT16/RALY/EIF4E/TBCA/PPP1CC/LONP1/TLR3/TPD52L2/SRPK2/FAM172A/EIF4G1/DNM1/CRKL/SUGP1/PDAP1/CSDE1/SHMT1/STAU1/BAG4/ENDOU/SRP14/TWF2/DNAJC21/SAFB2/PRDX1/JUN/SOX9/DUT/MORC3/CWC15/TPT1/LARP1/TCOF1/MRPL58/MKI67/GTPBP2/MARS1/EIF1AX/PCBP2/ZBP1/LRRFIP1/FARSA/PSIP1/NFX1/ARHGEF1/MTDH/CORO1A/CASC3/APEX1/TARBP2/C7orf50/PARP1/

					LBR/GIGYF2/FMR1/EIF2S2/HEXIM1/TRDMT1/TPR/MANF/LRRC59/EEF1D/PTPN1/TOP1/MYH9/ASS1/DTD1/EDF1/NPM1/SNRPB2/RBPMS2/WDR46/RBPMS/TRIM25/TDRKH/HDGF/SERPINH1/YY1/SMNDC1/RPGR/EIF2AK2/SART1/EIF4G3/LSM8
GOCC_CHROMOSOME	-0.5026	-2.3875	0.0000	0.0000	PPP1R12A/CCND2/CHMP6/ZNF174/BRD3/CBX2/CEBPA/BCL7A/IST1/MAGED1/XRCC4/HDAC8/PRC1/RFC4/PPP1CC/RUVBL1/FOS/CHEK2/ATF2/BIRC2/ZHX2/STAT5B/SRPK2/SUGT1/SATB1/RNF168/TRIM24/FOXJ3/CDK1/JUN/SOX9/BRD2/TP53BP1/BCL7B/CEBPB/HHEX/NFKB1/WAS/MAX/DYNLT3/NFATC3/MKI67/TP73/GLYR1/PSIP1/TERF1/NFX1/KDM3A/STAT2/ELOA/APEX1/ARNT/FOXO1/FH/PARP1/MORF4L1/FMR1/BACH1/GATA3/TPR/FEN1/TOP1/NFE2/NBN/NFATC1/DCTN6/PPP2R5A/NFYA/SIRT1/SEPTIN7/CALCOCO1/YY1/RASSF2/IRAG2/FOXO3/NFAT5

Table S 5: Results from STRING protein-protein-interaction analysis in symptomatic vs. asymptomatic DS.

Term ID	Term	Strength	FDR P value	matching proteins in the network
**Cluster 1**				
GO:0009605	Response to external stimulus	0.32	0.0075	CD4,TNFRSF1A,HMOX1,TNFRSF10A,CCL2,IGFBP2,GDF15,CHI3L1,NOTCH3,IL1RL2,CDH2,TNFRSF10B,DSC2,LY96,TIMP4,KIT,CXCL16,TNFRSF11B,RNASE6,CCL11,KLRD1,RET,EPHA2,CR2,CD55,WFDC2,TREM2,VSIG4,SPP1,LGALS9,CD14,IL6,GDNF,ADM,CLEC5A,CXCL17,NOS1,FAS
GO:0023052	Signaling	0.23	0.0075	CD4,TNFRSF1A,HMOX1,TIMP1,PLAT,TNFRSF10A,COMP,CCL2,IGFBP2,GDF15,CHI3L1,SYT1,NOTCH3,IL1RL2,CD27,OSMR,TNFRSF10B,DSC2,LY96,TIMP4,KIT,CXCL16,TNFRSF11B,CCL11,FGF5,CTHRC1,F3,KLRD1,TNFSF13,GFRA1,RET,EPHA2,CR2,SNCG,TREM2,ANGPTL2,SPP1,LGALS9,EDA2R,CD14,IL6,GDNF,FSHB,ADM,SMAD5,CLEC5A,NOS1,FAS,CD59
GO:0048518	Positive regulation of biological process	0.2	0.0075	CD4,TNFRSF1A,HMOX1,TIMP1,PLAT,TNFRSF10A,COMP,PCOLCE,CCL2,IGFBP2,CTSD,GDF15,GFAP,CHI3L1,MMP7,SYT1,NOTCH3,IL1RL2,CD27,CDH2,OSMR,TNFRSF10B,LY96,KIT,CXCL16,CCL11,FGF5,TNFRSF12A,CTHRC1,F3,KLRD1,TNFSF13,GFRA1,RET,EPHA2,CR2,CD55,TREM2,FABP3,VSIG4,SPP1,LGALS9,EDA2R,CD14,IL6,GDNF,FSHB,ADM,SMAD5,CLEC5A,CXCL17,NOS1,NEFL,HAVCR1,FAS
GO:0007154	Cell communication	0.22	0.0092	CD4,TNFRSF1A,HMOX1,TIMP1,PLAT,TNFRSF10A,COMP,CCL2,IGFBP2,GDF15,CHI3L1,SYT1,NOTCH3,IL1RL2,CD27,OSMR,TNFRSF10B,DSC2,LY96,TIMP4,KIT,CXCL16,TNFRSF11B,CCL11,FGF5,CTHRC1,F3,KLRD1,TNFSF13,GFRA1,RET,EPHA2,CR2,SNCG,TREM2,ANGPTL2,SPP1,LGALS9,EDA2R,CD14,IL6,GDNF,FSHB,ADM,SMAD5,CLEC5A,NOS1,FAS,CD59
GO:0048522	Positive regulation of cellular process	0.21	0.0105	CD4,TNFRSF1A,HMOX1,TIMP1,TNFRSF10A,COMP,CCL2,IGFBP2,CTSD,GDF15,GFAP,CHI3L1,MMP7,SYT1,NOTCH3,IL1RL2,CD27,CDH2,OSMR,TNFRSF10B,LY96,KIT,CXCL16,CCL11,FGF5,TNFRSF12A,CTHRC1,F3,KLRD1,TNFSF13,GFRA1,RET,EPHA2,CD55,TREM2,FABP3,SPP1,LGALS9,EDA2R,CD14,IL6,GDNF,FSHB,ADM,SMAD5,CXCL17,NOS1,NEFL,HAVCR1,FAS
GO:0006952	Defense response	0.36	0.0145	CD4,TNFRSF1A,HMOX1,TIMP1,CCL2,CHI3L1,IL1RL2,TNFRSF10B,LY96,KIT,CXCL16,RNASE6,CCL11,F3,KLRD1,EPHA2,CR2,CD55,WFDC2,TREM2,VSIG4,SPP1,LGALS9,CST3,CD14,IL6,ADM,CLEC5A
GO:0007165	Signal transduction	0.22	0.0269	CD4,TNFRSF1A,HMOX1,TIMP1,PLAT,TNFRSF10A,COMP,CCL2,IGFBP2,GDF15,CHI3L1,NOTCH3,IL1RL2,CD27,OSMR,TNFRSF10B,LY96,TIMP4,KIT,CXCL16,TNFRSF11B,CCL11,FGF5,CTHRC1,F3,KLRD1,TNFSF13,GFRA1,RET,EPHA2,CR2,TREM2,SPP1,LGALS9,EDA2R,CD14,IL6,GDNF,FSHB,ADM,SMAD5,CLEC5A,NOS1,FAS,CD59
GO:0010033	Response to organic substance	0.27	0.0269	CD4,TNFRSF1A,HMOX1,TIMP1,PLAT,COMP,CCL2,IGFBP2,GDF15,CHI3L1,IL1RL2,OSMR,LY96,TIMP4,KIT,CXCL16,TNFRSF11B,CCL11,FGF5,F3,CTSB,GFRA1,RET,EPHA2,TREM2,SPP1,LGALS9,EDA2R,CD14,IL6,FSHB,ADM,SMAD5,NOS1,NEFL,FAS
GO:0050896	Response to stimulus	0.14	0.0269	CD4,TNFRSF1A,HMOX1,TIMP1,PLAT,TNFRSF10A,COMP,CCL2,IGFBP2,GDF15,GFAP,CHI3L1,CTSV,SYT1,NOTCH3,IL1RL2,CD27,CDH2,OSMR,TNFRSF10B,DSC2,LY96,TIMP4,KIT,CXCL16,TNFRSF11B,RNASE6,CCL11,FGF5,CTHRC1,F3,KLRD1,KIR2DL3,TNFSF13,CTSB,GFRA1,RET,EPHA2,CR2,CD55,WFDC2,TREM2,VSIG4,LAIR1,SPP1,LGALS9,EDA2R,CST3,CD14,IL6,GDNF,FSHB,ADM,SMAD5,CLEC5A,CXCL17,NOS1,NEFL,FAS,CD59
GO:0006954	Inflammatory response	0.47	0.0462	TNFRSF1A,HMOX1,TIMP1,CCL2,CHI3L1,IL1RL2,LY96,KIT,CCL11,F3,EPHA2,TREM2,SPP1,LGALS9,CD14,IL6,ADM
GO:0060255	Regulation of macromolecule metabolic process	0.21	0.0470	CD4,TNFRSF1A,HMOX1,TIMP1,PLAT,TNFRSF10A,COMP,PCOLCE,CCL2,CTSD,GDF15,GFAP,CHI3L1,NOTCH3,IL1RL2,CD27,TNFRSF10B,LY96,TIMP4,KIT,FGF5,F3,TNFSF13,CTSB,GFRA1,RET,EPHA2,CD55,WFDC2,TREM2,VSIG4,SPP1,LGALS9,EDA2R,CST3,CD14,IL6,GDNF,FSHB,SMAD5,CLEC5A,CXCL17,NOS1,FAS
GO:0007166	Cell surface receptor signaling pathway	0.28	0.0498	CD4,TNFRSF1A,PLAT,TNFRSF10A,COMP,CCL2,GDF15,NOTCH3,IL1RL2,CD27,OSMR,TNFRSF10B,LY96,TIMP4,KIT,CCL11,FGF5,CTHRC1,F3,KLRD1,GFRA1,RET,EPHA2,CR2,TREM2,EDA2R,CD14,IL6,FSHB,SMAD5,FAS,CD59
GO:0050793	Regulation of developmental process	0.29	0.0498	CD4,TNFRSF1A,HMOX1,COMP,CCL2,GDF15,GFAP,CHI3L1,SYT1,NOTCH3,IL1RL2,CD27,CDH2,KIT,TNFRSF11B,CCL11,CTHRC1,F3,TNFSF13,RET,EPHA2,TREM2,SPP1,LGALS9,CST3,IL6,GDNF,FSHB,ADM,SMAD5,NEFL
GO:0051716	Cellular response to stimulus	0.17	0.0498	CD4,TNFRSF1A,HMOX1,TIMP1,PLAT,TNFRSF10A,COMP,CCL2,IGFBP2,GDF15,GFAP,CHI3L1,SYT1,NOTCH3,IL1RL2,CD27,OSMR,TNFRSF10B,DSC2,LY96,TIMP4,KIT,CXCL16,TNFRSF11B,CCL11,FGF5,CTHRC1,F3,KLRD1,TNFSF13,CTSB,GFRA1,RET,EPHA2,CR2,TREM2,SPP1,LGALS9,EDA2R,CD14,IL6,GDNF,FSHB,ADM,SMAD5,CLEC5A,CXCL17,NOS1,NEFL,FAS,CD59
GO:0038023	Signaling receptor activity	0.44	0.0092	CD4,TNFRSF1A,TNFRSF10A,NOTCH3,IL1RL2,CD27,OSMR,TNFRSF10B,LY96,KIT,TNFRSF11B,SIGLEC7,F3,KLRD1,KIR2DL3,GFRA1,RET,EPHA2,CR2,TREM2,EDA2R,CD14,FAS
GO:0005035	Death receptor activity	1.26	0.0132	TNFRSF1A,TNFRSF10A,TNFRSF10B,EDA2R,FAS
GO:0004888	Transmembrane signaling receptor activity	0.46	0.0159	CD4,TNFRSF1A,TNFRSF10A,IL1RL2,CD27,OSMR,TNFRSF10B,KIT,F3,KLRD1,GFRA1,RET,EPHA2,CR2,TREM2,EDA2R,CD14,FAS
GO:0005615	Extracellular space	0.19	0.0340	TNFRSF1A,HMOX1,TIMP1,PLAT,COMP,PCOLCE,CCL2,IGFBP2,CTSD,GDF15,CHI3L1,CTSV,MMP7,TINAGL1,DSC2,LY96,TIMP4,KIT,CXCL16,TNFRSF11B,RNASE6,CCL11,FGF5,CTHRC1,F3,TNFSF13,CTSB,GFRA1,CR2,CD55,SNCG,WFDC2,ANGPTL2,FABP3,MSLN,SPP1,LGALS9,CST3,CD14,IL6,GDNF,FSHB,ADM,CXCL17,FAS,CD59
GO:0043235	Receptor complex	0.54	0.0340	CD4,TNFRSF1A,NOTCH3,OSMR,LY96,KIT,TNFRSF11B,KLRD1,GFRA1,RET,EPHA2,CR2,CD14,IL6
GO:0045121	Membrane raft	0.63	0.0340	CD4,TNFRSF1A,HMOX1,TNFRSF10A,CTSD,CDH2,RET,CD55,TREM2,CD14,NOS1,FAS
hsa04060	Cytokine-cytokine receptor interaction	0.58	0.0008	CD4,TNFRSF1A,TNFRSF10A,CCL2,GDF15,IL1RL2,CD27,OSMR,TNFRSF10B,CXCL16,TNFRSF11B,CCL11,TNFRSF12A,TNFSF13,IL6,CXCL17,FAS
HSA-140534	Caspase activation via Death Receptors in the presence of ligand	1.26	0.0377	TNFRSF10A,TNFRSF10B,LY96,CD14,FAS
HSA-168256	Immune System	0.3	0.0377	CD4,TNFRSF1A,HMOX1,TIMP1,CCL2,CTSD,CHI3L1,CTSV,IL1RL2,CD27,OSMR,LY96,TNFRSF11B,RNASE6,CCL11,SIGLEC7,TNFRSF12A,KLRD1,KIR2DL3,TNFSF13,CTSB,CR2,CD55,TREM2,LAIR1,LGALS9,EDA2R,CST3,CD14,IL6,CLEC5A,NOS1,CD59

GOCC:0005576	Extracellular region	0.29	0.0000	CD4,TNFRSF1A,HMOX1,TIMP1,PLAT,COMP,PCOLCE,CCL2,IGFBP2,CTSD,GDF15,CHI3L1,CTSV,MMP7,CD27,CDH2,TINAGL1,LY96,TIMP4,KIT,CXCL16,TNFRSF11B,RNASE6,CCL11,FGF5,KLK4,CTHRC1,F3,CTSB,GFRA1,CR2,CD55,WFDC2,TREM2,ANGPTL2,FABP3,MSLN,SPP1,LGALS9,CST3,CD14,IL6,GDNF,FSHB,ADM,CXCL17,FAS,CD59
GOCC:0005615	Extracellular space	0.33	0.0200	CD4,TNFRSF1A,HMOX1,TIMP1,PLAT,CCL2,IGFBP2,CTSD,GDF15,CHI3L1,MMP7,KIT,CXCL16,RNASE6,CTHRC1,F3,CTSB,CR2,CD55,FABP3,SPP1,LGALS9,CST3,IL6,FSHB,ADM,FAS,CD59
GOCC:0043235	Receptor complex	0.51	0.0455	CD4,TNFRSF1A,NOTCH3,CD27,OSMR,LY96,TNFRSF11B,KLRD1,RET,CR2,SPP1,CD14,IL6,ADM
KW-0325	Glycoprotein	0.25	0.0000	CD4,TNFRSF1A,TIMP1,PLAT,TNFRSF10A,COMP,PCOLCE,CCL2,IGFBP2,CTSD,GDF15,CHI3L1,CTSV,SYT1,NOTCH3,IL1RL2,CD27,CDH2,TINAGL1,OSMR,DSC2,LY96,KIT,CXCL16,TNFRSF11B,RNASE6,CCL11,FGF5,SIGLEC7,KLK4,CTHRC1,F3,KLRD1,KIR2DL3,TNFSF13,CTSB,GFRA1,RET,EPHA2,CR2,CD55,WFDC2,TREM2,ANGPTL2,MSLN,LAIR1,SPP1,EDA2R,CST3,CD14,IL6,GDNF,FSHB,CLEC5A,NEFL,HAVCR1,FAS,CD59
KW-0732	Signal	0.24	0.0000	CD4,TNFRSF1A,TIMP1,PLAT,TNFRSF10A,COMP,PCOLCE,CCL2,IGFBP2,CTSD,GDF15,CHI3L1,CTSV,MMP7,NOTCH3,IL1RL2,CD27,CDH2,TINAGL1,OSMR,TNFRSF10B,DSC2,LY96,TIMP4,KIT,CXCL16,TNFRSF11B,RNASE6,CCL11,FGF5,SIGLEC7,KLK4,TNFRSF12A,CTHRC1,F3,KIR2DL3,CTSB,GFRA1,RET,EPHA2,CR2,CD55,WFDC2,TREM2,ANGPTL2,VSIG4,MSLN,LAIR1,SPP1,CST3,CD14,IL6,GDNF,FSHB,ADM,CXCL17,HAVCR1,FAS,CD59
KW-1015	Disulfide bond	0.27	0.0000	CD4,TNFRSF1A,TIMP1,PLAT,TNFRSF10A,COMP,PCOLCE,CCL2,IGFBP2,CTSD,GDF15,CHI3L1,CTSV,NOTCH3,IL1RL2,CD27,TINAGL1,OSMR,TNFRSF10B,LY96,TIMP4,KIT,CXCL16,TNFRSF11B,RNASE6,CCL11,SIGLEC7,KLK4,TNFRSF12A,F3,KLRD1,KIR2DL3,TNFSF13,CTSB,GFRA1,RET,EPHA2,CR2,CD55,WFDC2,TREM2,ANGPTL2,VSIG4,MSLN,LAIR1,EDA2R,CST3,CD14,IL6,GDNF,FSHB,ADM,CLEC5A,CXCL17,HAVCR1,FAS,CD59
KW-0675	Receptor	0.44	0.0006	CD4,TNFRSF1A,TNFRSF10A,NOTCH3,IL1RL2,CD27,OSMR,TNFRSF10B,KIT,TNFRSF11B,TNFRSF12A,KLRD1,KIR2DL3,GFRA1,EPHA2,CR2,CD55,TREM2,LAIR1,EDA2R,CLEC5A,HAVCR1,FAS
KW-0964	Secreted	0.27	0.0007	TNFRSF1A,TIMP1,PLAT,COMP,PCOLCE,CCL2,IGFBP2,CTSD,GDF15,CHI3L1,MMP7,TINAGL1,LY96,TIMP4,CXCL16,TNFRSF11B,RNASE6,CCL11,FGF5,KLK4,CTHRC1,F3,TNFSF13,CTSB,CD55,WFDC2,TREM2,ANGPTL2,MSLN,SPP1,LGALS9,CST3,CD14,IL6,GDNF,FSHB,ADM,CXCL17,FAS,CD59
IPR001368	TNFR/NGFR cysteine-rich region	1.14	0.0040	TNFRSF1A,TNFRSF10A,CD27,TNFRSF10B,TNFRSF11B,EDA2R,FAS
IPR000488	Death domain	1.18	0.0429	TNFRSF1A,TNFRSF10A,TNFRSF10B,TNFRSF11B,FAS
SM00208	Tumor necrosis factor receptor / nerve growth factor receptor repeats.	1.1	0.0014	TNFRSF1A,TNFRSF10A,CD27,TNFRSF10B,TNFRSF11B,EDA2R,FAS
SM00005	DEATH domain, found in proteins involved in cell death (apoptosis).	1.22	0.0065	TNFRSF1A,TNFRSF10A,TNFRSF10B,TNFRSF11B,FAS
**Cluster 2**				
GO:0005201	Extracellular matrix structural constituent	1.44	0.0009	DCN,MATN2,ELN,DPT,FBLN2
GO:0050840	Extracellular matrix binding	1.49	0.0051	DCN,CD248,ELN,FBLN2
GO:0031012	Extracellular matrix	1.02	0.0000	DCN,MATN2,LTBP2,CD248,ELN,DPT,EFEMP1,MFAP4,FBLN2
GO:0062023	Collagen-containing extracellular matrix	1.06	0.0000	DCN,MATN2,LTBP2,ELN,DPT,EFEMP1,MFAP4,FBLN2
GO:0071953	Elastic fiber	2.46	0.0153	ELN,MFAP4
CL:19697	Elastic fibre formation, and Transforming growth factor beta receptor complex assembly	2.09	0.0000	DCN,LTBP2,ELN,EFEMP1,MFAP4,FBLN2
CL:19699	Elastic fibre formation	2.31	0.0000	LTBP2,ELN,EFEMP1,MFAP4,FBLN2
HSA-2129379	Molecules associated with elastic fibres	1.9	0.0000	LTBP2,ELN,EFEMP1,MFAP4,FBLN2
HSA-1474244	Extracellular matrix organization	1.11	0.0012	DCN,LTBP2,ELN,EFEMP1,MFAP4,FBLN2
GOCC:0030312	External encapsulating structure	1.28	0.0000	DCN,MATN2,LTBP2,CD248,ELN,EFEMP1,MFAP4,FBLN2
GOCC:0031012	Extracellular matrix	1.23	0.0000	DCN,MATN2,LTBP2,CD248,ELN,EFEMP1,MFAP4,FBLN2
GOCC:0062023	Collagen-containing extracellular matrix	1.12	0.0071	DCN,MATN2,ELN,MFAP4,FBLN2
GOCC:0071953	Elastic fiber	2.46	0.0179	ELN,MFAP4
GOCC:0001527	Microfibril	2.06	0.0497	ELN,MFAP4

KW-0272	Extracellular matrix	1.24	0.0000	DCN,LTBP2,ELN,DPT,EFEMP1,MFAP4,FBLN2
KW-0245	EGF-like domain	1.19	0.0002	MATN2,LTBP2,CD248,ITGBL1,EFEMP1,FBLN2
KW-0964	Secreted	0.48	0.0245	DCN,MATN2,LTBP2,ELN,DPT,ITGBL1,EFEMP1,MFAP4,FBLN2
IPR000742	EGF-like domain	1.2	0.0011	MATN2,LTBP2,CD248,ITGBL1,EFEMP1,FBLN2
IPR001881	EGF-like calcium-binding domain	1.38	0.0011	MATN2,LTBP2,CD248,EFEMP1,FBLN2
IPR009030	Growth factor receptor cysteine-rich domain superfamily	1.31	0.0013	MATN2,LTBP2,CD248,EFEMP1,FBLN2
IPR000152	EGF-type aspartate/asparagine hydroxylation site	1.4	0.0066	MATN2,LTBP2,EFEMP1,FBLN2
IPR018097	EGF-like calcium-binding, conserved site	1.38	0.0066	LTBP2,CD248,EFEMP1,FBLN2
IPR026823	Complement Clr-like EGF domain	1.79	0.0066	MATN2,EFEMP1,FBLN2
SM00179	Calcium-binding EGF-like domain	1.46	0.0002	MATN2,LTBP2,CD248,EFEMP1,FBLN2
SM00181	Epidermal growth factor-like domain.	1.16	0.0020	MATN2,LTBP2,CD248,ITGBL1,FBLN2
**Cluster 3**				
GO:0008233	Peptidase activity	1.11	0.0102	CPA1,CELA2A,CTRB1,CPB1,PRSS2
GO:0016787	Hydrolase activity	0.77	0.0188	CPA1,CELA2A,CTRB1,RNASE1,CPB1,PRSS2
CL:19325	Mixed, incl. Pancreatitis, and Carboxypeptidase activation peptide	2.38	0.0000	CPA1,CELA2A,CTRB1,CPB1,PRSS2
CL:19328	Carboxypeptidase activation peptide, and Pancreatitis	2.38	0.0000	CPA1,CELA2A,CTRB1,CPB1
CL:19331	Carboxypeptidase activation peptide, and Digestion	2.55	0.0001	CPA1,CTRB1,CPB1
hsa04972	Pancreatic secretion	1.79	0.0001	CPA1,CELA2A,CTRB1,CPB1
hsa04974	Protein digestion and absorption	1.75	0.0001	CPA1,CELA2A,CTRB1,CPB1
BTO:0000988	Pancreas	1.05	0.0182	CPA1,CELA2A,CTRB1,RNASE1,CPB1
KW-0378	Hydrolase	0.86	0.0018	CPA1,CELA2A,CTRB1,RNASE1,CPB1,PRSS2
KW-0645	Protease	1.17	0.0018	CPA1,CELA2A,CTRB1,CPB1,PRSS2
KW-0865	Zymogen	1.26	0.0046	CPA1,CELA2A,CTRB1,PRSS2
KW-0222	Digestion	2.38	0.0070	CTRB1,PRSS2
KW-0720	Serine protease	1.36	0.0216	CELA2A,CTRB1,PRSS2
PF02244	Carboxypeptidase activation peptide	2.28	0.0247	CPA1,CPB1
**Cluster 4**				
GO:0016055	Wnt signaling pathway	1.6	0.0078	SFRP1,WNT9A,RSPO1,DKK3
CL:21053	ncRNAs involved in Wnt signaling in hepatocellular carcinoma, and Regulation of FZD by ubiquitination	2.18	0.0000	SFRP1,WNT9A,RSPO1,DKK3
CL:21056	Wnt signaling in kidney disease, and Negative	2.24	0.0002	SFRP1,WNT9A,DKK3

	regulation of TCF-dependent signaling by WNT ligand antagonists			
CL:21060	Wnt signaling in kidney disease, and Negative regulation of non-canonical Wnt signaling pathway	2.28	0.0158	SFRP1,WNT9A
hsa04310	Wnt signaling pathway	1.7	0.0063	SFRP1,WNT9A,RSPO1
HSA-201681	TCF dependent signaling in response to WNT	1.8	0.0173	SFRP1,WNT9A,RSPO1
HSA-3772470	Negative regulation of TCF-dependent signaling by WNT ligand antagonists	2.28	0.0383	SFRP1,WNT9A
KW-0879	Wnt signaling pathway	1.78	0.0001	SFRP1,WNT9A,RSPO1,DKK3
**Cluster 5**				
KW-0722	Serine protease inhibitor	1.66	0.0101	AMBP,SERPINA11,ITIH3
**Cluster 6**				
CL:38327	Mixed, incl. Chordin-like protein 1/2, and Positive regulation of corticotropin-releasing hormone secretion	2.8	0.0153	CHRDL1,MAMDC2
KW-0892	Osteogenesis	2.28	0.0246	FSTL3,CHRDL1
**Cluster 7**				
GO:0038084	Vascular endothelial growth factor signaling pathway	2.31	0.0014	VEGFB,NRP2,PGF
GO:0001938	Positive regulation of endothelial cell proliferation	1.8	0.0118	VEGFB,NRP2,PGF
GO:0060754	Positive regulation of mast cell chemotaxis	2.58	0.0247	VEGFB,PGF
GO:0005172	Vascular endothelial growth factor receptor binding	2.44	0.0381	VEGFB,PGF
GO:0008201	Heparin binding	1.46	0.0381	VEGFB,NRP2,PGF
CL:17400	VEGF ligand-receptor interactions	2.5	0.0428	VEGFB,PGF
HSA-194138	Signaling by VEGF	1.94	0.0031	VEGFB,NRP2,PGF
HSA-195399	VEGF binds to VEGFR leading to receptor dimerization	2.44	0.0223	VEGFB,PGF
DOID:1727	Retinal vein occlusion	2.98	0.0075	VEGFB,PGF
DOID:4449	Macular retinal edema	2.98	0.0075	VEGFB,PGF
DOID:4692	Endophthalmitis	2.98	0.0075	VEGFB,PGF
DOID:9191	Diabetic macular edema	2.98	0.0075	VEGFB,PGF
KW-0358	Heparin-binding	1.68	0.0052	VEGFB,NRP2,PGF
IPR000072	PDGF/VEGF domain	2.44	0.0341	VEGFB,PGF
IPR023581	Platelet-derived growth factor, conserved site	2.5	0.0341	VEGFB,PGF
SM00141	Platelet-derived and vascular endothelial growth factors (PDGF, VEGF) family	2.44	0.0089	VEGFB,PGF

**Cluster 8**				
**Cluster 9**				
GO:0044278	Cell wall disruption in another organism	2.86	0.0294	REG1A,REG1B
GO:0042834	Peptidoglycan binding	2.55	0.0159	REG1A,REG1B
GO:0070492	Oligosaccharide binding	2.5	0.0159	REG1A,REG1B
CL:31688	Cell wall disruption in another organism, and Alpha-defensin	2.76	0.0107	REG1A,REG1B
BTO:0000650	Endocrine pancreas	2.25	0.0486	REG1A,REG1B
**Cluster 10**				
CL:31852	Mixed, incl. Lipid-binding serum glycoprotein, N-terminal, and Secretoglobin	2.46	0.0337	BPIFB1,SCGB3A1
**Cluster 11**				
PF10591	Secreted protein acidic and rich in cysteine Ca binding region	2.86	0.0012	SMOC1,SPOCK1
IPR019577	SPARC/Testican, calcium-binding domain	2.76	0.0085	SMOC1,SPOCK1
IPR000716	Thyroglobulin type-1	2.31	0.0244	SMOC1,SPOCK1
IPR036857	Thyroglobulin type-1 superfamily	2.31	0.0244	SMOC1,SPOCK1
IPR002350	Kazal domain	2.11	0.0280	SMOC1,SPOCK1
IPR036058	Kazal domain superfamily	2.1	0.0280	SMOC1,SPOCK1
SM00211	Thyroglobulin type I repeats.	2.31	0.0100	SMOC1,SPOCK1
SM00280	Kazal type serine protease inhibitors	2.11	0.0115	SMOC1,SPOCK1
**Cluster 12**				
CL:20160	Mixed, incl. Adamantinoma, and AJAP1/PANP, C-terminal	2.68	0.0143	NPDC1,COLEC12
**Cluster 13**				
GO:0042599	Lamellar body	2.55	0.0103	SFTPA2,CKAP4
GO:0005791	Rough endoplasmic reticulum	2.18	0.0240	SFTPA2,CKAP4
GOCC:0042599	Lamellar body	2.46	0.0176	SFTPA2,CKAP4

Table S 6: Results from LASSO feature selection with corresponding mean NPX levels and FDR corrected P value from initial t-test analysis.

Proteins	sDS	aDS	FDR P value from t test
GFAP	1.268	-0.205	0.000
NFL	1.283	-0.193	0.000
IGFBP2	0.625	-0.441	0.000
EDA2R	0.580	-0.136	0.000
SPON1	0.365	-0.169	0.000
CXCL17	0.697	-0.182	0.000
CBLN4	-0.402	0.008	0.001
CD14	0.370	-0.080	0.010
SEPTIN3	0.810	-0.133	0.027
ASAH2	-0.438	0.067	0.041
SCGB3A1	0.242	-0.096	0.041
ANGPTL2	0.416	-0.080	0.044
KIR2DL2	0.578	-0.088	0.064
FLT3	-0.209	0.004	0.296
CGN	-0.113	0.250	0.331

Figure S 1: PPI STRING network of DEP between DS and HC colored according to MCL clustering. Each node represents one DEP while each edge represents functional interaction between two DEP with the thickness of the edge increasing depending on the confidence (score 0 – 1) of the proposed shared function.

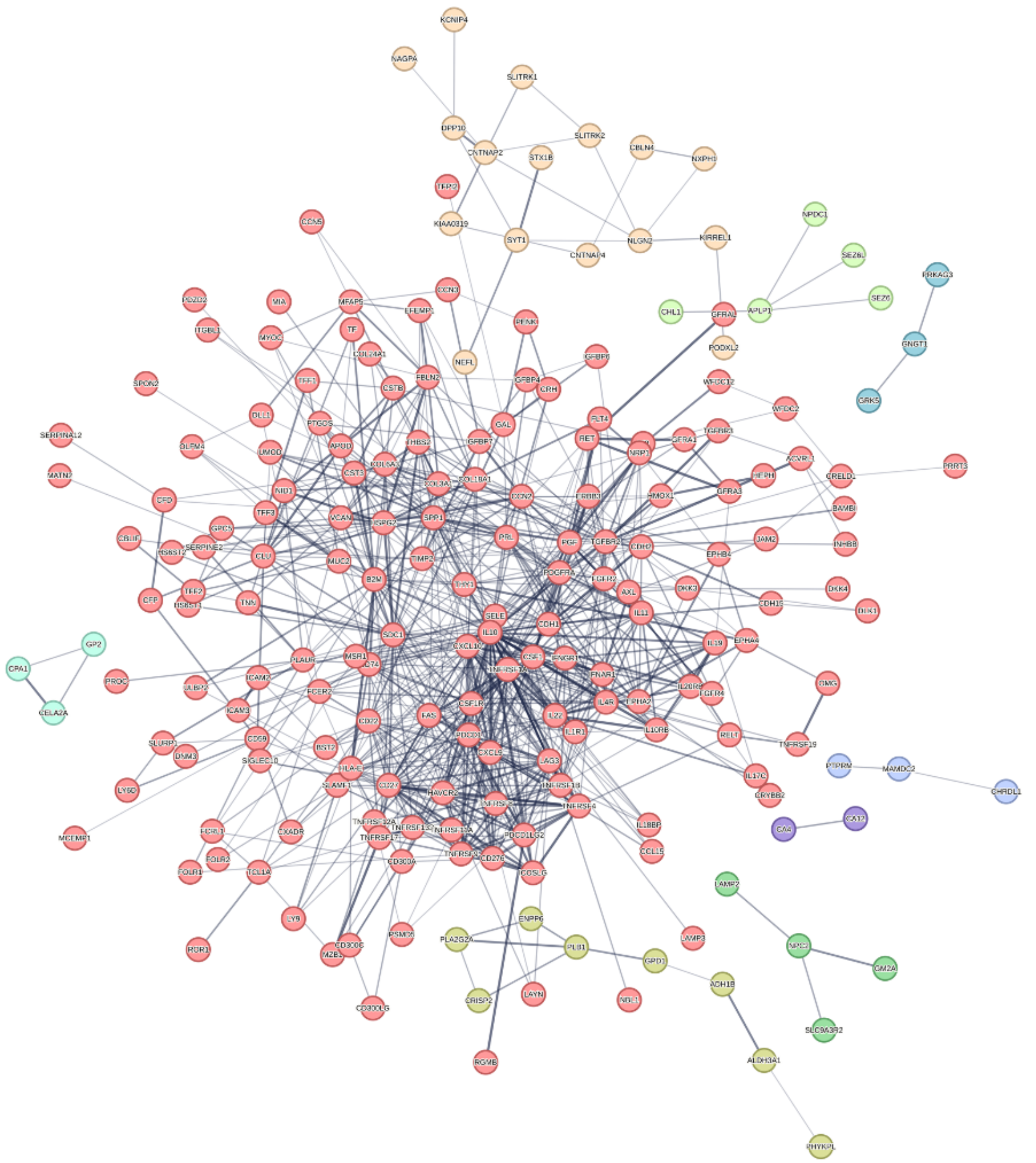

Figure S 2: Visualization of LASSO Feature Selection in symptomatic vs. asymptomatic DS.

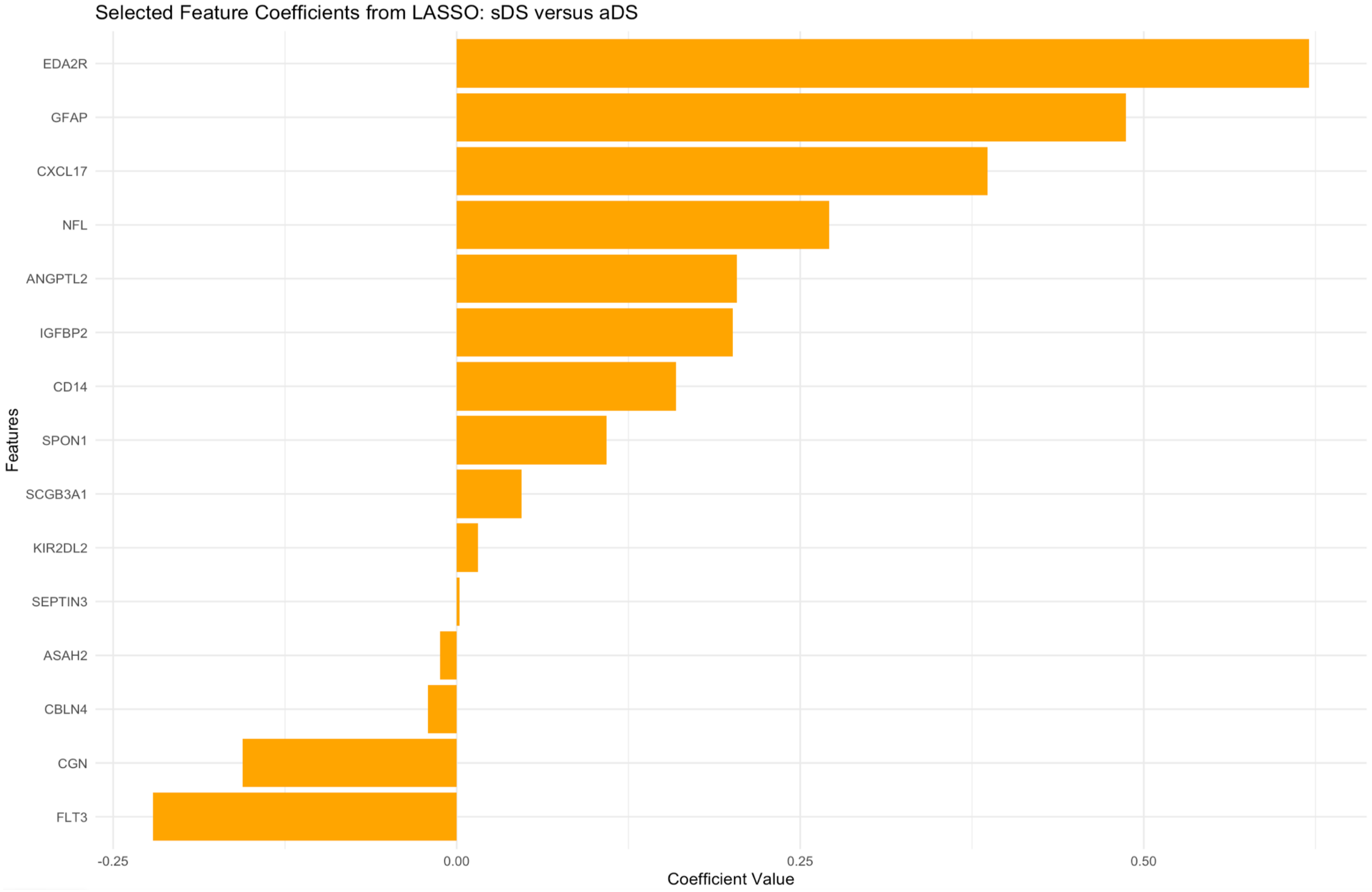

Supplement: Supplementary file 2 — Supporting Information [file ALZ-21-e70040-s002.pdf]
